# Differentiation of Symbiotic Cells and Endosymbionts in *Medicago truncatula* Nodulation Are Coupled to Two Transcriptome-Switches

**DOI:** 10.1371/journal.pone.0009519

**Published:** 2010-03-04

**Authors:** Nicolas Maunoury, Miguel Redondo-Nieto, Marie Bourcy, Willem Van de Velde, Benoit Alunni, Philippe Laporte, Patricia Durand, Nicolas Agier, Laetitia Marisa, Danièle Vaubert, Hervé Delacroix, Gérard Duc, Pascal Ratet, Lawrence Aggerbeck, Eva Kondorosi, Peter Mergaert

**Affiliations:** 1 Institut des Sciences du Végétal, Centre National de la Recherche Scientifique, Unité Propre de Recherche 2355, Gif-sur-Yvette, France; 2 Centre de Génétique Moléculaire, Centre National de la Recherche Scientifique, Formation de Recherche en Evolution 3144 and Gif/Orsay DNA MicroArray Platform (GODMAP), Gif-sur-Yvette, France; 3 Université Paris-Sud 11, Orsay, France; 4 Génétique et Ecophysiologie des Légumineuses à Graines, Institut National de la Recherche Agronomique, Dijon, France; 5 Bay Zoltan Foundation for Applied Research, Institute of Plant Genomics, Human Biotechnology and Bioenergy, Szeged, Hungary; Ecole Normale Superieure, France

## Abstract

The legume plant *Medicago truncatula* establishes a symbiosis with the nitrogen-fixing bacterium *Sinorhizobium meliloti* which takes place in root nodules. The formation of nodules employs a complex developmental program involving organogenesis, specific cellular differentiation of the host cells and the endosymbiotic bacteria, called bacteroids, as well as the specific activation of a large number of plant genes. By using a collection of plant and bacterial mutants inducing non-functional, Fix^−^ nodules, we studied the differentiation processes of the symbiotic partners together with the nodule transcriptome, with the aim of unravelling links between cell differentiation and transcriptome activation. Two waves of transcriptional reprogramming involving the repression and the massive induction of hundreds of genes were observed during wild-type nodule formation. The dominant features of this “nodule-specific transcriptome” were the repression of plant defense-related genes, the transient activation of cell cycle and protein synthesis genes at the early stage of nodule development and the activation of the secretory pathway along with a large number of transmembrane and secretory proteins or peptides throughout organogenesis. The fifteen plant and bacterial mutants that were analyzed fell into four major categories. Members of the first category of mutants formed non-functional nodules although they had differentiated nodule cells and bacteroids. This group passed the two transcriptome switch-points similarly to the wild type. The second category, which formed nodules in which the plant cells were differentiated and infected but the bacteroids did not differentiate, passed the first transcriptome switch but not the second one. Nodules in the third category contained infection threads but were devoid of differentiated symbiotic cells and displayed a root-like transcriptome. Nodules in the fourth category were free of bacteria, devoid of differentiated symbiotic cells and also displayed a root-like transcriptome. A correlation thus exists between the differentiation of symbiotic nodule cells and the first wave of nodule specific gene activation and between differentiation of rhizobia to bacteroids and the second transcriptome wave in nodules. The differentiation of symbiotic cells and of bacteroids may therefore constitute signals for the execution of these transcriptome-switches.

## Introduction

Legumes establish a nitrogen-fixing symbiosis with bacteria belonging to the family of *Rhizobiaceae* (rhizobia). This symbiosis takes place in *de novo* induced organs, the root nodules, and is based on nutrient exchange. The bacterium provides ammonium from nitrogen fixation to the plant which, in turn, supplies the bacterium with carbohydrates derived from photosynthesis. The symbiosis allows these plants to grow on nitrogen poor soils and to produce high protein-containing leaves and seeds.

**Figure 1:**
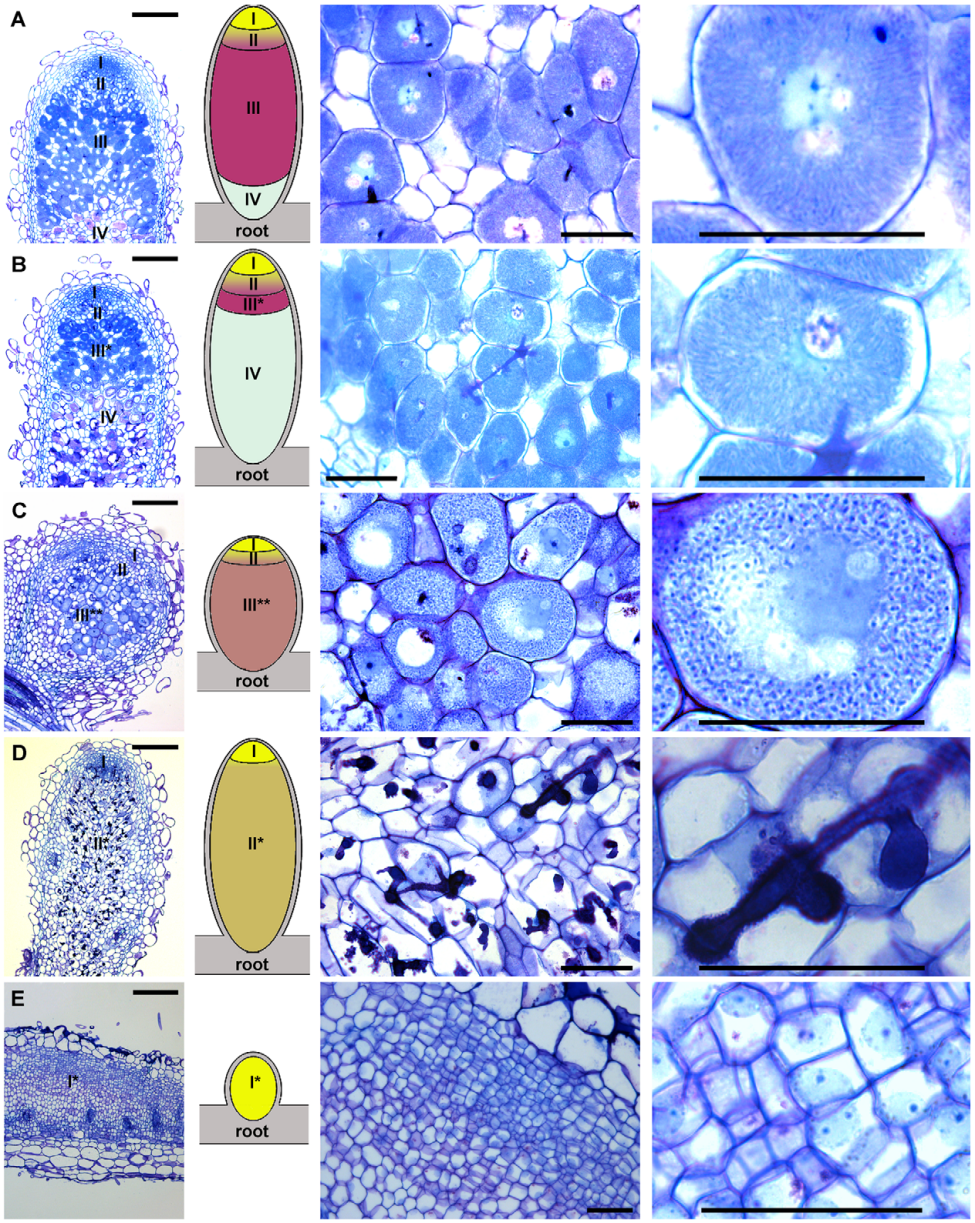
Nodule structure and infection in wild type and Fix^−^ mutants. Semi-thin longitudinal nodule sections were stained with toluidine blue and observed by light microscopy. (A) J5-Sm1021 Wild type; (B) TRV43-Sm1021; (C) J5-Sm1021*bacA*; (D) TR3-Sm1021; (E) V1-Sm1021. (Left panels) Tissue organisation of nodules including a corresponding schematic illustration. Tissues distinguishable in the nodule are I, meristem; I* in (E), primordium-like structure with dividing cells; II, infection and differentiation zone; II* in (D), zone of infection without cellular differentiation; III, nitrogen fixation zone; III* in (B), zone with fully differentiated cells that, however, do not fix nitrogen; III** in (C), zone with differentiated plant cells in which bacteroids are not differentiated; IV, zone of symbiotic cell senescence; root and nodule cortical tissues are in grey. Bars equal 200 µm. (Middle panels) Enlargement of the central area of the nodules showing the presence or absence of differentiated symbiotic cells. Bars equal 50 µm. (Right panels) Images of symbiotic cells showing the structure of the intracellular bacteria (A–C) or the absence of differentiation and bacteria in the cells in the central area of the nodule (D,E). Bars equal 50 µm.

The development of nodules relies on a continuous molecular dialogue between the two symbionts and successive steps lead to the completion of nodule formation [1], [2]. The steps in nodule development and the final histological organization of the organ may differ considerably among legumes. In the *Medicago truncatula*-*Sinorhizobium meliloti* interaction, the initial exchange of signals, which involves plant-derived flavonoids and *Rhizobium*-derived Nod factors, induces cell division in the inner root cortex thus leading to the formation of a nodule primordium. These signals also permit the rhizobia to infect the host plant via tubular structures called infection threads. The infection threads are initially formed in root hairs and then progress and ramify to the cortex and to the cells of the incipient organ. The rhizobia in infection threads are incorporated into differentiating cells by endocytosis. Internalized rhizobia, which are encapsulated by a plant-derived membrane, differentiate into nitrogen-fixing bacteroids, forming organelle-like structures called symbiosomes. At the distal end of the incipient nodule, a group of uninfected cells constitutes a meristem, the continuous growth of which results in a mature nodule with several central histological zones surrounded by peripheral tissues (Figure 1A). The apical meristem is zone I. Zone II is the infection zone, in which post-mitotic cells differentiate and where bacteria are taken up by plant cells from continuously growing infection threads. In zone III, the symbiotic cells are mature and fix nitrogen. Older nodules also may have a root proximal senescence zone (zone IV) composed of degenerating symbiotic cells [3].

Symbiotic cells originate from progenitor cells in the meristem. During their differentiation, the cells exit the cell division cycle, which is converted into an endoreduplication cycle. These post-mitotic cells are infected and gradually become filled with increasing numbers of symbiosomes. In parallel, they increase their size considerably by successive endoreduplication cycles. Mature symbiotic cells are about 80-fold larger in size and have endoploidy levels up to 64C as compared to diploid 2C cells in Zone I [4], [5]. The cytosolic space of these cells is entirely packed with symbiosomes. Further, the bacteroids in the symbiosomes undergo a remarkable, irreversible or terminal differentiation which involves cell elongation, genome amplification and the loss of reproductive capability [6], [7].

It has been shown that, during the nodulation process, there are major changes in plant gene expression [8]–[14]. Several hundreds of genes (the “nodule-specific transcriptome”) have been identified that are specifically and strongly up- or down-regulated during the nodulation process. The large majority of these genes are activated at later stages of organogenesis as deduced from the observation that mutants which are affected in the early signalling between the symbiotic partners fail to activate this “nodule-specific transcriptome” [8]. However, the link between the transcriptome activation and the key events of nodule formation - the establishment of a primordium, plant cell infection and cell differentiation of host and endosymbiont - remains unknown. An analysis of gene expression in *Medicago* nodules induced by bacterial mutants or formed by plant mutants suggested that nodule development is defined by a discrete number of transcriptional stages [15], [16]. However, these studies were conducted with a small number of genes and the developmental stages that were affected by these mutants were not precisely defined.

The objective of our study was to investigate the links between gene expression, the consecutive steps of nodule development and the differentiation of the prokaryotic and eukaryotic partners. To this end, nodule invasion and symbiotic plant cell and bacteroid differentiation were studied in nodules impaired in their development as a result of bacterial or plant mutations at different loci. The transcriptome of the mutant nodules was compared to a reference transcriptome characteristic of wild type symbiosis. The phenotypic characterization of the nodules along with the analysis of gene expression in the mutants demonstrated that the differentiation of the symbiotic cells and of the bacteroids constitute two key events in the activation of genes during nodulation and the establishment of the “nodule-specific transcriptome”.

## Results

### Histological Characterization of Nodulation Mutants

Since several of the mutants that we studied (Table S1) have been described only partially or not at all, we decided to establish a phenotypic description for all the mutants in order to confer a common framework for the interpretation of the transcriptome results. The tissue organization (zonation) and the bacterial infection of the nodules were analyzed by histochemical staining and light microscopy. Semi-thin longitudinal sections stained with toluidine blue (Figure 1 and S1) and staining of rhizobia in thick longitudinal sections using a constitutively expressed *lacZ* marker gene (Figure S2 and S3) demonstrated four classes of mutant nodules.

The majority of the mutants (the TR36, TR183, TRV36 and TRV43 plant mutants in the Jemalong J5 background and the *S. meliloti* Sm2011 mutants in the *nifH*, *nifA*, *fixG*, *fixJ*, *fixK* and *lpsB* genes) gave rise to nodules of the first class. The nodules were elongated, similarly to wild type nodules, with zonation and invasion by bacteria (Figure 1A, 1B, S1, S2, S3A and S3B). Endocytotic uptake of the rhizobia occurred normally and typical, large symbiotic cells, fully packed with bacteroids, were present (Figure 1B and S1). However, only two weeks after inoculation, the nodules of these mutants already exhibited a senescence zone. In comparison, in wild type nodules, this senescent zone generally appears two weeks later. In these mutants, the senescence zone expanded very rapidly and, in 4 week old nodules, the fixation zone was reduced to a few cell layers. In nodules of the TRV36 mutant, the senescence zone also contained a large number of brown-coloured necrotic cells (Figure S2C). All of the class 1 mutants contained a visible infection thread network in the senescence zone (Figure S2D, S3A and S3B) as well as saprophytic rhizobia invading senescent plant cells (Figure S2C and S2E) similar to what has been described for aging-induced senescence in wild type nodules [17]. Our observations are in agreement with previous descriptions of the *lpsB* [18], [19], *nifH* [20] and *fixJ* and *fixG* [6] mutants.

Nodules of the second class, induced by the *S. meliloti* Sm1021*bacA* mutant, were white, either remained small or became elongated and contained differentiated and infected symbiotic cells (Figure 1C and S3C) [21].

The third class consists of the *M. truncatula* allelic mutants TR3 and TE7 [22]. In these mutants, the nodules were white and frequently elongated, indicating the formation of an active meristem. The nodules were infected but the bacteria were confined to an extended infection thread network and the central region was free of nitrogen fixing symbiotic cells (Figure 1D and S3D) [22].

The fourth class comprised the *M. truncatula* R108 mutant V1. This mutant produces small white nodules without zonation and without any infection threads or bacteria (Figure 1E and S3E). This pattern is similar to that observed in the nodules induced by the *S. meliloti* Sm2011*exoY* mutant [23], [24].

**Figure 2:**
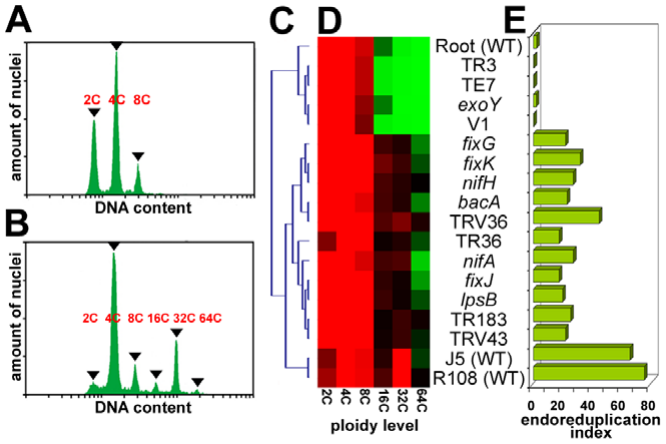
Ploidy levels in nodule cells as determined by flow cytometry. (A,B) Measurement of the DNA content in the nuclei of roots (A) and in wild type nodules (B). The x-axis is DAPI fluorescence (DNA content) and the y-axis is the number of counts (amount of nuclei). The peaks at 2C, 4C, 8C, 16C, 32C and 64C are indicated with arrowheads. (C) Arborescence of the hierarchical cluster analysis of the data in (D). (D) Heat map for the hierarchical cluster analysis of the ploidy levels in the nodules of the mutants used in this study. The columns correspond to the type of nuclei at 2C, 4C, 8C, 16C, 32C and 64C. The colour code is red, high, black, intermediate and green, low relative amounts of nuclei expressed in %. Each row corresponds to a nodule or a root sample. The identity of the samples is indicated at the right of the heat map. (E) Endoreduplication index expressing the relative amounts of 16C, 32C and 64C nuclei in nodules. The identity of the samples is as in (D).

The microscopic analysis of nodules clearly demonstrated the presence of large symbiotic cells, about 50 µm in diameter, in the first two mutant types (Figure 1B and 1C), similarly to the wild type (Figure 1A) whereas such large cells were absent in nodules of the third and fourth mutant types (Figure 1D and 1E). The formation of large symbiotic cells is driven by endoreduplication [4], [5]. The measurement by flow cytometry of ploidy levels in *M. truncatula* roots showed the presence of nuclei up to 8C (Figure 2A) whereas wild type nodules contained nuclei with a DNA content up to 64C (Figure 2B). The highly endoreduplicated nuclei with DNA contents of 16C, 32C and 64C originate from differentiating or mature symbiotic cells [4], [5], [25]. We measured the ploidy levels in all of the mutant nodules and we used the presence of highly polyploid cells with DNA contents of 16C, 32C and 64C as a marker for symbiotic cell differentiation. Two types of mutants could be distinguished by cluster analysis of the nuclear ploidy measurements (Figure 2C). The *M. truncatula* mutants TR3, TE7 and V1 and the *S. meliloti* mutant *exoY* formed nodules with a ploidy pattern similar to that of the root with a low (<2) endoreduplication index (Figure 2D and 2E). This indicates the absence of symbiotic cell differentiation in these nodules and is in agreement with the histological analysis. In the other mutants (the *M. truncatula* TR36, TR183, TRV36 and TRV43 mutants and the *S. meliloti nifH*, *nifA*, *fixG*, *fixJ*, *fixK*, *lpsB* and *bacA* mutants), the nodules had a ploidy profile similar to that of the wild type (Figure 2D) along with a high endoreduplication index (>15, Figure 2E). These results indicate the formation of differentiated symbiotic cells and confirm the histological analysis.

**Figure 3:**
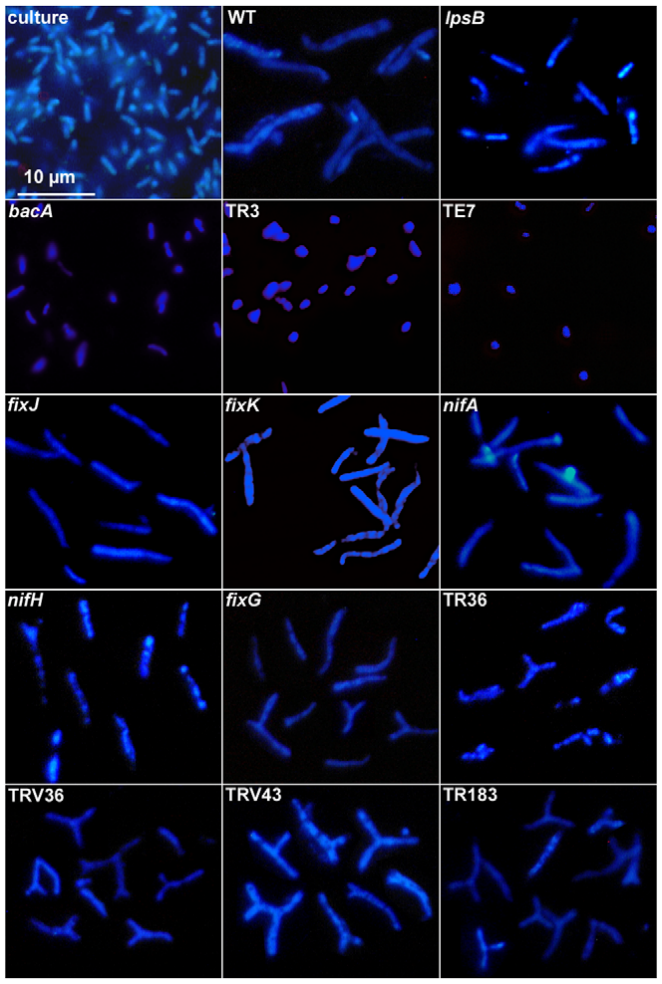
Differentiation of bacteroids in nodules. Bacteroids were isolated from wild type and mutant nodules, stained with DAPI and visualized with fluorescence microscopy. The scale bar is the same for all panels.

Microscopic examination at higher magnification of symbiotic cells in mutant nodules of the first type revealed the presence of elongated bacteria, visible as filamentous structures in the cells (Figure 1B and S1) suggesting that bacteroids differentiated similarly to those in wild type nodules (Figure 1A). In contrast, the bacteria in the symbiotic cells of the second type of mutant nodule were not elongated (Figure 1C). To confirm whether or not terminal bacteroid differentiation has taken place, we analyzed, by microscopy, the morphology of bacteria isolated from the different types of mutant nodules (Figure 3). Cultured *S. meliloti* bacteria were small, 1–2 µm long rod shaped cells whereas fully differentiated bacteroids present in the wild type nodules were 4–5 fold longer (Figure 3) [6], [7]. The nodules of the *M. truncatula* TR36, TR183, TRV36 and TRV43 mutants as well as nodules induced by the *S. meliloti nifH*, *nifA*, *fixG*, *fixJ*, *fixK*, and *lpsB* mutants contained elongated bacteroids similar to those observed in wild type nodules which indicates that terminal differentiation of the bacteroids has occurred in these mutants (Figure 3). In contrast, the bacteria isolated from the nodules of the mutants TR3, TE7 and *bacA* showed no signs of differentiation by elongation and the rhizobia isolated from these nodules were morphologically similar to cultured rhizobia (Figure 3). Finally, the nodules of the V1 and *exoY* mutants were devoid of any bacteria (data not shown) in agreement with the results of histochemical staining.

### Analysis of Gene Expression in Developing, Wild Type Nodules (the “Nodule-Specific Transcriptome”)

**Figure 4:**
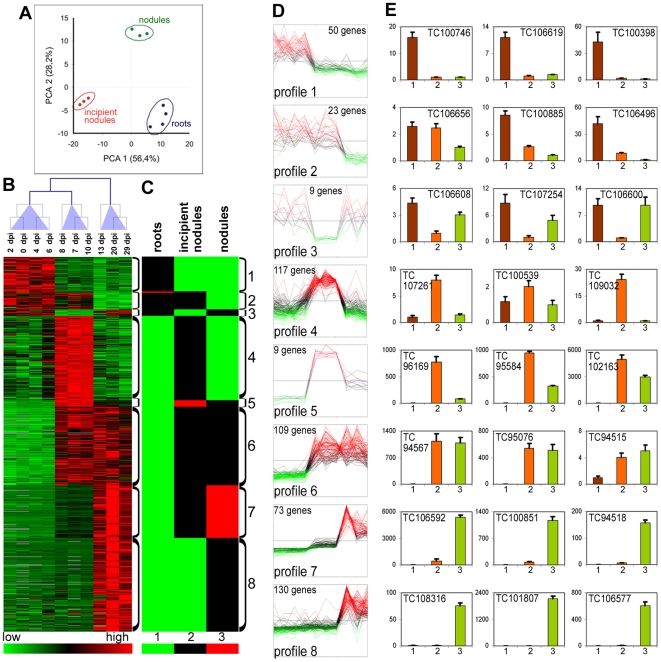
The transcriptome of wild type *M. truncatula* nodules. (A) PCA analysis of microarray experiments. The two principal components and their fraction of the overall variability of the data (%) are shown on the x-axis and the y-axis. Clusters of experiments are circled and annotated as “roots”, containing time points 0, 2, 4, 6 dpi, “incipient nodules”, containing time points 7, 8, 10 dpi and “nodules”, containing time points 13, 20 and 29 dpi. (B) Heat map of the hierarchical cluster analysis of the microarray hybridization experiments. The columns correspond to the different time points indicated above the map and the arborescence indicates the similarity among transcriptomes. Below the heat map is a colour coded scale bar for the relative expression levels of genes. (C) Heat map of the levels of expression converted to integer values (1, 2 or 3 as indicated in the colour coded scale bar below the heat map) which indicate statistical differences (*p*<0.01) and gene expression strength in numerical order. (D) Expression profiles for all the genes in the profile measured by microarray analysis. The order of the points in the curves is 0, 2, 4, 6, 7, 8, 10, 13, 20 and 29 dpi. The same colour code as in (B) is used. (E) RT-qPCR measurements of expression patterns for 3 selected genes from each expression profile. The histograms are organized in one row per profile and annotated with the MtGI accession numbers. The relative expression levels, which correspond to the fold change relative to the sample with the lowest value (arbitrarily set to 1), are shown for R108 roots, 1 (brown bars), immature nodules, induced by Sm41, at 8 dpi, 2 (orange bars) and mature nodules at 15 dpi, 3 (green bars). The error bars correspond to the standard deviations for 3 biological repetitions.

Principal component analysis (PCA) and cluster analysis (Figure 4A and 4B) of the transcriptomes obtained from wild type nodules (*M. truncatula* R108 plants inoculated with *S. meliloti* Sm41) at 10 developmental time-points (0, 2, 4, 6, 7, 8, 10, 13, 20, 29 days post inoculation (dpi)) gave 3 homogeneous clusters with respect to the time points. The first cluster consists of root samples collected at the early time points (0–6 dpi) before the macroscopically visible appearance of nodules. The second cluster contained the samples corresponding to incipient nodules which were still white, indicative for the absence of nitrogen fixation (7–10 dpi). These immature nodules had already formed a distal meristem zone and an infection zone where cells are invaded by infection threads (Figure S4A–C). Also a few enlarged and infected symbiotic cells were present, proximal to the root, but intracellular bacteria were not yet elongated and differentiated (Figure S4D and S4E). The last cluster (>13 dpi) consisted of the stages where nodules were mature and nitrogen-fixing as was indicated by their pink colour. These nodules had the typical nodule zonation and contained symbiotic cells with fully differentiated bacteroids (Figure S4F–H).

To identify genes differentially expressed in one of the three clusters, t-tests were performed and genes were selected with a *p*-value<0.01. Five hundred and twenty genes were found to be differentially expressed (Table S2). By hierarchical clustering, these 520 genes were assigned to 8 distinct temporal profiles of expression (Figure 4B–D). RT-qPCR performed on independent samples taken from uninoculated roots, immature nodules (8 dpi) and mature nodules (15 dpi) was also used to define temporal expression profiles of selected genes in each cluster and corroborate the results obtained by microarray analysis (Figure 4E).

The first three expression profiles were characteristic of genes which were repressed or temporarily repressed during the formation of the nodules (Figure 4B–E). Remarkably, among the genes having these profiles, those potentially involved in stress responses, e.g. genes in the phenylpropanoid/flavonoid/lignin/phytoalexin pathways (Table S2) were particularly common. This involvement in the stress response, for many of the genes having these profiles, was also obvious from the EST distribution in the *M. truncatula* Gene Index (MtGI): ESTs were abundant in libraries from elicited cell suspensions and/or tissues infected with pathogens or insects (Table S2). These results suggest that in contrast to pathogens, rhizobia enter a plant tissue where the defense reactions have not been induced but on the contrary have been repressed.

The fourth expression profile was exhibited by genes the induction of which was detectable only in the immature nodules (Figure 4B–E). The group of genes exhibiting this profile was strongly enriched in ribosomal genes and other genes involved in protein synthesis or protein trafficking (68 genes out of 117). This suggests an enhanced metabolic activity in this stage which may be related to the cell division activity in the incipient nodules. Indeed, this group also contained several genes that code for known cell cycle regulators like cyclin, cyclin-dependent kinase, histone as well as other genes associated with cell division in nodule and root promordia such as a WD-repeat-containing protein and an UDP-Glycosyltransferase [26], [27]. The group also contained the genes coding for the peroxidase Rip1 [28] and the membrane protein MtN3 [29] whose known expression patterns are in agreement with our microarray measurements. The fifth expression profile is similar to the fourth, however, the genes have levels of expression in mature nodules that are higher than in roots although being significantly lower than in incipient nodules. The genes *MtMMPL1* (*MtN9*) and *MtN6* have been described previously and have profiles that match perfectly the profile that was identified here [30], [31].

The remaining 3 gene expression profiles (6, 7 and 8) were characteristic of genes that were expressed in mature nodules (Figure 4B–E). The expression of these genes was activated in two waves. A first wave of expression was induced early, in immature nodules (profile 6) and the second wave (profile 8) only in mature nodules. Genes exhibiting profile 7 display a first slight induction at the early nodule stage (first wave) but attained maximum expression only in mature nodules (second wave).

**Figure 5:**
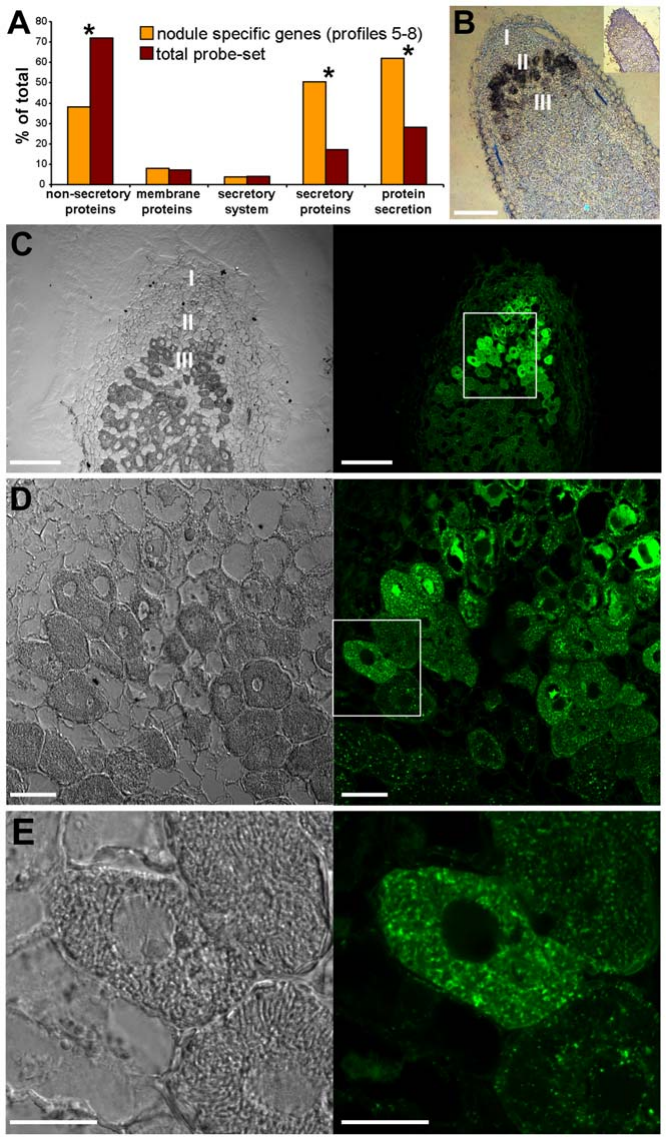
The secretory pathway is strongly activated in nodule zones II and III. (A) Distribution of genes exhibiting expression profiles 5–8 (321 genes) and the total probe-set (2366 genes) into categories of the secretory pathway. A significant enrichment or depletion of categories, supported by Fisher's exact test, is indicated by an asterisk (*). The *p*-values are 10^−42^ for the category “non-secretory proteins”, 10^−52^ for the category “secretory proteins” and 10^−42^ for the category “protein secretion” (combined categories “membrane proteins”, “secretory system” and “secretory proteins”). (B) *In situ* hybridization with an antisense probe of the *SPP* gene. The position of nodule zones I, II and III are indicated. *SPP* expression is observed as a black signal in the proximal zone II. The inset shows a control hybridization with a sense probe. (C–E) Immunolocalization of the ER specific KDEL marker MAC 256. On the left are the DIC images and on the right are the fluorescence images. (D) is a magnification of the region outlined in (C). (E) is an enlargement of the region outlined in (D). Scale bars are 100 µm (B,C), 20 µm (D) and 10 µm (E).

Strikingly, the majority of genes exhibiting profiles 5–8 are linked to the secretory pathway. Using the BLAST [32], SignalP and TMHHH [33] algorithms, we found that 50% of genes in these clusters coded for secreted proteins, which are characterized by the presence of a signal peptide, as compared to 17% of the genes in the total probe-set of the microarray, which is a significant enrichment (*p* = 10^−52^) (Figure 5A). In addition, 25 genes (8%) coded for transmembrane proteins and 12 genes (4%) encoded components of the secretory system itself. Thus, in all, 62% of the genes exhibiting profiles 5–8 are dedicated to protein secretion via the secretory pathway as compared to 28% of the genes in the total probe-set (*p* = 10^−42^) (Figure 5A). The majority of the transmembrane proteins were predicted to be transporters (16 genes) whereas one was a receptor-like kinase. The others are of unknown function. The secreted protein-encoding genes fell into a few groups: 31 genes encoded proteins with presumed functions as catalytic enzymes and 113 genes encoded peptides that had less than 220 amino acids, including the processed signal peptide. The principal groups of peptides were the NCR cysteine-rich peptides (98 genes) [34], [35] and the glycine-rich peptides (8 genes) [35], [36].

One of the upregulated genes of the secretory pathway encodes a signal peptide peptidase (SPP). SPP is an enzyme of the endoplasmic reticulum (ER) that catalyzes intramembrane proteolysis of signal peptides after they have been cleaved from a preprotein by a signal peptidase [34], [37]. Our *in situ* hybridization shows that the *SPP* gene was expressed in differentiating symbiotic cells in zone II of the nodule (Figure 5B). Using an antibody for the immuno-detection of the ER, which is the gateway of the secretory pathway, we observed that the ER is particularly well developed in differentiating symbiotic cells of the nodule zone II and in young mature cells of zone III (Figure 5C–E). These results suggest that the secretory pathway is particularly active in these cells which, also, is in agreement with the prominent presence of genes of the secretory pathway in the “nodule-specific transcriptome”.

Among the soluble proteins encoded by genes exhibiting expression profiles 5–8, we identified several interesting signalling genes, besides the expected genes involved in primary metabolism, particularly those involved in carbon/nitrogen metabolism for nitrogen assimilation and in the nutrition of bacteroids (Table S2). These included a Mitogen Activated Protein Kinase or MAPK, a calmodulin binding protein, a phosphatase 2C and remarkably, 11 different transcription factors which probably participate in the regulation of the nodule-specific transcriptome. Two of these transcription factors, MtHAP2-1 and MtNIN, have been found, indeed, to be essential for nodule development [38], [39].

### Transcriptome Analysis in Nodulation Mutants

In order to further investigate the relationship between cell differentiation and transcriptome activation and the waves of transcriptional reprogramming that were observed during wild-type nodule formation we next studied nodules impaired in their development due to mutations in the plant (7 mutants) or the bacterial (8 mutants) symbiotic partner. The set of 520 genes that we found to be differentially expressed during wild type nodule formation (wild type temporal profile data set) was used as a reference set for the comparison of the transcriptomes of the Fix^−^ nodules of the different mutants (mutant data set) and those of wild type nodules in the Jemalong J5 background induced by *S. meliloti* Sm1021 or Sm2011.

**Figure 6:**
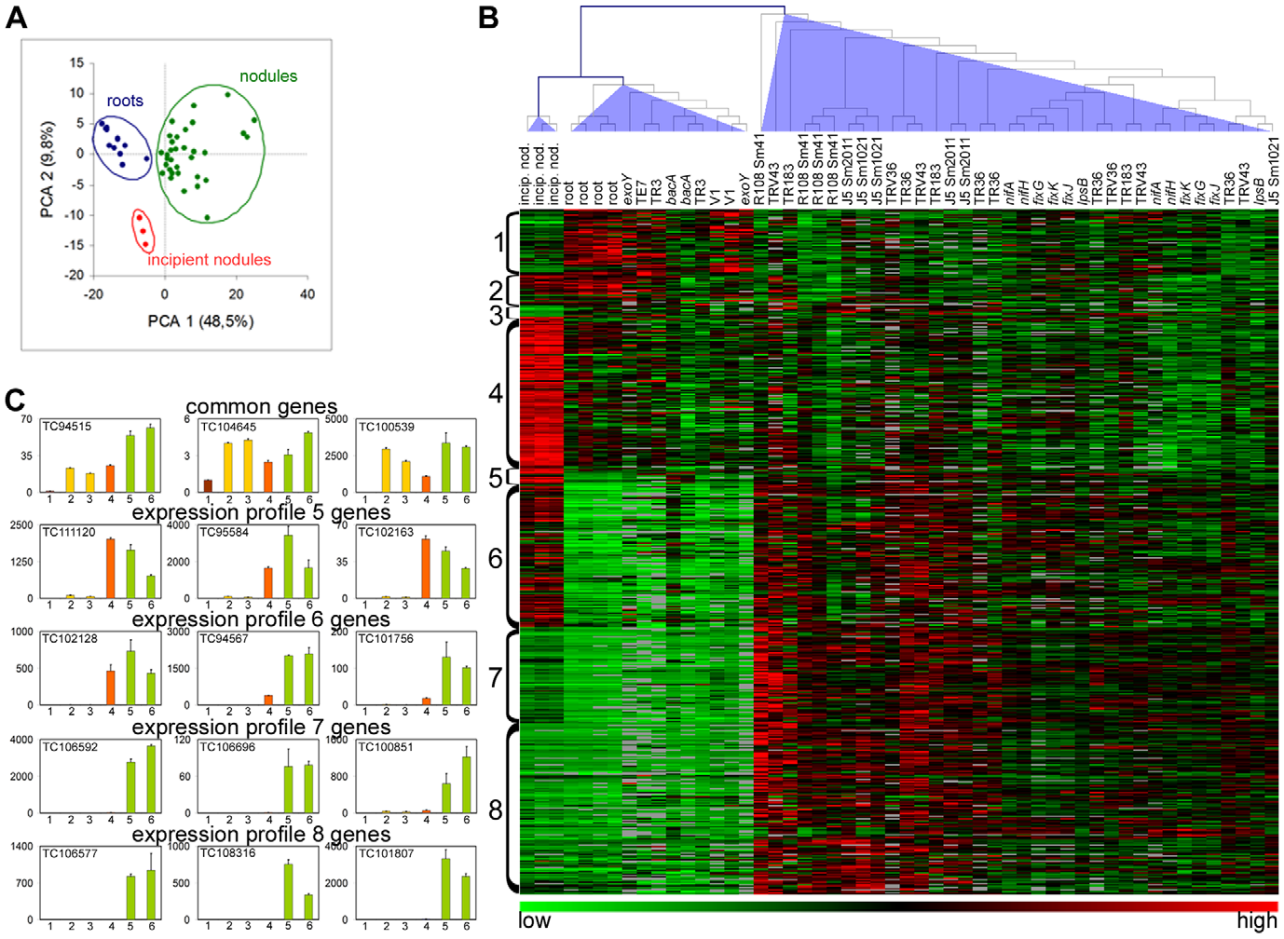
The transcriptome in non-functional, Fix^−^ nodules of *M. truncatula*. (A) PCA analysis of the microarray experiments. The two principal components and their fraction of the overall variability of the data (%) are shown on the x-axis and on the y-axis. Clusters of experiments are circled and annotated as “roots”, containing root samples and nodules from mutants Sm2011*exoY*, Sm1021*bacA*, TR3, TE7 and V1; “incipient nodules” containing wild type immature nodules; and “nodules” containing wild type nodules and nodules from the *M. truncatula* mutants TR183, TR36, TRV36, TRV43 and the *S. meliloti* mutants *lpsB*, *nifA*, *fixJ*, *fixK*, *fixG* and *nifH*. (B) Heat map of transcriptomes of nodulation mutants. The samples are annotated above the columns. The arborescence above the columns shows the similarities among the transcriptomes. The gene expression profiles that were identified in the wild type temporal analysis (see Figure 4) are indicated at the left. The colour coded scale bar for the relative levels of expression of the genes is indicated below the heat map. (C) RT-qPCR analysis of the expression patterns for 3 genes, selected among the ensemble, which exhibit expression profiles 5, 6, 7 and 8 and for genes expressed in all the mutants (common genes). The histograms are annotated with MtGI accession numbers. The samples in the histograms are as follows: J5 wild type roots, 1 (brown bars); nodules from TR3 induced by Sm1021, 2, and from TE7 induced by Sm1021, 3, (orange bars); nodules from J5 induced by Sm1021*bacA*, 4, (red bars); and nodules from TR36 induced by Sm1021, 5, and J5 wild type nodules induced by Sm1021, 6 (green bars). The relative expression levels correspond to the fold change relative to the sample with the lowest value (arbitrarily set to 1). The error bars correspond to the standard deviations for 3 biological repetitions.

PCA analysis of the mutant data set combined with the wild type temporal profile data set revealed three homogeneous clusters (Figure 6A). The first cluster grouped the 3 samples from the immature nodule stage of the wild type nodule formation. The second cluster contained the samples from roots along with samples from the Fix^−^ nodules of the *bacA* and *exoY* gene bacterial mutants and the plant mutants TE7, TR3 and V1. The third and largest cluster was defined by the samples from wild type nodules in the R108 or J5 background and induced by the *S. meliloti* strains Sm41, Sm1021 or Sm2011 and from the Fix^−^ nodules of the plant mutants TR183, TR36, TRV36, TRV43 and of the *lpsB*, *nifA*, *fixJ*, *fixK*, *fixG* and *nifH* gene bacterial mutants. Hierarchical clustering of these same data also divided the experimental samples into three major clusters (Figure 6B) the compositions of which were identical to those obtained by PCA. Since PCA and hierarchical cluster analysis does not separate the R108 and J5 *M. truncatula* lines or the Sm41, Sm1021 and Sm2011 *S. meliloti* strains, we conclude that the plant or bacterial genetic background of the *M. truncatula* or the *S. meliloti* species has little effect on the overall expression profiles. However, this does not exclude the possibility that subtle differences exist between the genetic backgrounds as has been demonstrated for example for Sm1021 and Sm2011 [40].

We next examined whether, in the Fix^−^ nodules, the levels of expression of the same groups of genes that defined the waves of induction in the temporal profiles that were observed during wild type nodule differentiation could indicate to what extent the mutant nodules had traversed these waves of induction. First, it should be noted that the wild type incipient nodule stages were clearly separated from the other experiments and that none of the plant or bacterial mutants were grouped with this cluster. The wild type incipient nodule stage was characterized by the strong expression of genes that exhibited profiles 4, 5 and 6 in the wild type temporal profile data set (Figure 6B). The heat map of the members of the second cluster, composed of the wild type root samples and the samples from the plant mutants TE7, TR3 and V1 and the bacterial mutants *exoY* and *bacA*, shows that, there was an absence of induction in genes that exhibited profiles 4, 5, 6, 7 and 8 in the wild type temporal profile data set (except for *bacA*, see below). The third and largest cluster was composed of samples from wild type nodules and the samples from the plant mutants TR36, TR183, TRV36, TRV43 and TRV183 and the bacterial mutants *lpsB*, *nifA*, *fixJ*, *fixK*, *fixG* and *nifH*. This cluster was characterized by a strong induction in the genes that had profiles 5, 6, 7 and 8 in the wild type temporal profile data set whereas the incipient nodule-specific profile 4 genes were not activated.

The transcriptome in the nodules induced by the *S. meliloti bacA* mutant closely resembled the root transcriptome (Figure 6B). Nevertheless, the heat map suggested that temporal profile 5 and 6 genes were activated (Figure 6B). This observation was verified by RT-qPCR experiments for selected genes exhibiting temporal profiles 5, 6, 7 and 8 in the wild type temporal profile data set. We found that the selected genes from the profiles 5 and 6 were indeed activated in nodules of the *S. meliloti bacA* mutant similarly to wild type and the TR36 mutant but they were not activated in roots or in nodules of the TR3 and TE7 mutants (Figure 6C). On the other hand, genes selected from the profile 7 and 8 groups were only activated in the nodules of wild type and the TR36 plant mutant but not in roots or the other mutant nodules (Figure 6C). Therefore, we conclude that the first wave of transcriptional reprogramming with activation of profiles 5 and 6 is taking place in nodules of the *S. meliloti bacA* mutant but not the second wave with activation of profiles 7 and 8.

Although the transcriptomes of the nodule-like structures in the mutants *exoY*, TE7, TR3 and V1 are similar to the transcriptomes of roots in that the large majority of nodule-specific genes were not activated, we were able to identify a small number of genes that were induced in all these mutants (*p*<0.01) (Table S3). The level of expression of these genes, however, was lower than in the wild type nodules. Interestingly, among them, we found a number of genes that are known to be associated with primordium initiation or meristem activity, such as the genes encoding a WD-repeat-containing protein [26], an UDP-glycosyltransferase [27], the MtHAP2-1 transcription factor [38] and the *enod40* gene [41]. Also, genes known to be expressed in the nodule cortex and to be involved in the creation of an oxygen-diffusion barrier, such as *enod2* [42] and carbonic anhydrase [43], were activated in these mutants. Remarkably, leghemoglobin genes were also slightly induced. This is possibly related to the early creation of the oxygen-diffusion barrier in these mutants. RT-qPCR on a few selected examples confirmed the induction of these genes in the TR3, TE7 and *S. meliloti bacA* mutants (Figure 6C).

## Discussion

### The Nodule-Specific Transcriptome

The organogenesis of nodules is accompanied by important changes in the transcriptome involving several hundreds of genes (this work and [8], [13]). The “nodule-specific transcriptome” that we identified here exhibits three major characteristics.

First, the repression of plant defense-related genes is probably necessary to avoid repulsion of the infecting rhizobia. Rhizobia possess “microorganism-associated molecular patterns” or MAMPs which are capable of eliciting the innate immune responses of the host plant whereas other rhizobial effectors, notably surface polysaccharides, are thought to attenuate or suppress this defense reaction during infection of root tissues for nodulation (for a review, see [1]). Mutations in rhizobia affecting the production of these surface polysaccharides often lead to arrested infections and/or nodule development accompanied by the induction of defense responses (see, for example [12], [44]). Further, purified rhizobial polysaccharides can suppress elicitor-induced defense responses *in vitro* [45], [46]. A recent study using elicitor-treated cell cultures of *M. truncatula* identified a set of genes that were induced by the elicitor treatment but showed a tempered induction by the presence of sinorhizobial LPS [47]. This gene set partially overlaps the genes that we identified here and includes genes encoding phenylalanine ammonia-lyase, chalcone synthases, isoflavone reductases and peroxidases. Thus, the suppression of expression that we observed for these genes during nodule formation could have been due to a direct effect of the infecting rhizobia via their LPS.

Second, a transient activation of cell cycle and protein synthesis genes at the incipient stage of nodule development reflects an increase in cell division at this stage of nodule organogenesis. The nodule originates from root cortical cells which are non-dividing and probably in a state of low metabolic activity. The initiation of the nodule formation requires dedifferentiation of these root cortical cells and induction of cell division which is in agreement with the induction of these cell cycle and protein synthesis genes. In *M. truncatula* nodules, cell division is maintained in the nodule meristem during the complete lifetime of the organ. Therefore, the transient nature of the gene expression suggests that the extent of cell division in the incipient nodule is higher than in the nodule meristem of mature nodules.

Finally, the activation of the secretory pathway and a large number of secretory proteins and/or peptides throughout organogenesis can be understood in the context of a major activity of symbiotic cells in the hosting and maintenance of the symbiosomes. The secretory pathway ensures the trafficking in cells of transmembrane or soluble proteins that are destined to reside extracellularly, in vacuoles, in the phragmoplast of dividing cells or, in the case of the symbiotic nodule cells, in infection threads or symbiosomes [48], [49]. Thus, our results indicate that a major specific activity of symbiotic cells is the secretion of proteins and peptides, most likely towards the symbiosomes. The upregulated components of the secretory pathway that we identified may be involved in specific protein traffic towards symbiosomes. Indeed, the nodule specific *DNF1* gene (Table S2, profile 6, TC96231) encodes a subunit of the signal peptidase complex that removes the signal peptide of secretory proteins during their translocation into the ER lumen. We and others have demonstrated recently that *DNF1* is involved in protein transport to symbiosomes and that the gene is essential for bacteroid differentiation and nitrogen fixation [50], [51]. A recently identified syntaxin protein, MtSYP132 is strongly induced in nodules (data not shown) and localized on the symbiosome membranes in symbiotic cells [52], [53]. Syntaxins are SNARE proteins that mediate fusions between secretory pathway vesicles and target membranes. MtSYP132 is thus probably another specific regulator of protein traffic towards symbiosomes. In addition, a Rab7 small GTPase was identified that could regulate, in conjunction with MtSYP132, vesicule docking and fusion on the symbiosome membrane [53].

A number of the secretory proteins that we have identified in this study have been shown, either in *Medicago* or in other legume species, to be transported to the symbiosomes, symbiosome lumen (peribacteroid space) or the bacteroids. These include the membrane proteins aquaporin nodulin 26 [54] and the sulfate transporter SST1 [55] and the soluble NCR peptides [50] and proteins ENOD8 [56], a calmodulin-like protein [57] and a lectin [58]. The membrane transporters probably have a role in communication or exchange of metabolites between the host cell and the endosymbiont. Likewise, the soluble proteins and peptides may be involved in symbiosome formation or functioning and bacteroid differentiation [50].

### Two Waves of Gene Expression Reprogramming during Nodule Formation and Four Categories of Fix^−^ Mutants

Our dissection of nodule formation identified two major events of transcriptional reprogramming (waves), one that is detectable at the immature nodule stage and a second one when nodules reach maturity. Both waves involve activation and repression of gene transcription which can be transient or permanent and, altogether, these waves were divided into 8 different expression profiles.

Several genes exhibiting expression profiles 5 and 6 have been found to be expressed in zone II or zone I and II of the nodule (*MtN9* [30], *MtN6* [31], *enod20* [59], *enod40* [41], *NCR084* [34], *GRP* [36], *MtHAP2* [38] and *NIN* [60]). On the other hand, genes such as leghemoglobin, *NCR001* [34], *enod8* [56], CaM-like protein [57], and asparagine synthase [61], which exhibit expression profiles 7 and 8, are known to be expressed in nodule zone III. These results suggest that the temporal expression profiles defined here correlate with a spatial distribution in the nodule tissue. Therefore, genes exhibiting expression profiles 5 and 6 are most probably expressed in nodule zone I and/or II whereas genes belonging to expression profiles 7 and 8 are most probably expressed in zone III of the nodule.

By combining histological, cytological and transcriptome data obtained from symbiotic mutants forming non-functional nodules, four categories of mutants were discerned. The V1 and Sm2011*exoY* mutants can induce nodule-like structures but fail to infect them. In these empty nodules, the “nodule-specific transcriptome” is nearly completely absent. In the second category, the allelic TR3 and TE7 mutants are filled with an infection thread network but lack infected and differentiated symbiotic cells. Also, in these mutants, the “nodule-specific transcriptome” is not induced. In the third category, represented by *S. meliloti bacA*, infected symbiotic cells are formed but bacteria in the symbiosomes fail to differentiate. Here, the first wave of transcriptome activation takes place but not the second one. In the last category, represented by the plant mutants TR183, TR36, TRV36, TRV43 and the bacterial mutants *lpsB*, *nifA*, *fixJ*, *fixK*, *fixG* and *nifH*, symbiotic cells are formed and infected with differentiated bacteroids but symbiotic cells undergo senescence prematurely because of the absence of effective nitrogen fixation. These mutants are associated with a transcriptome that is highly similar to a wild type transcriptome. It is remarkable that, in this type of mutant, the nodule organogenesis program, which is costly, is maintained rather than aborted through arrest of meristematic activity and prevention of further cell infection. This indicates that detection of the failure of nitrogen fixation and the subsequent induction of early senescence is a cell autonomous process. Such a mechanism might be valuable when nodules are infected with multiple strains having differing nitrogen-fixation efficiency, such as may occur outside the laboratory [62], thus permitting the selective elimination of non-functional symbiotic cells.

These conclusions, based on the analysis of large gene sets, correlate well with previous studies based on a small number of genes [15], [16]. Thus, surprisingly, nodule formation in *M. truncatula* follows a relatively simple transcriptional scheme with two major transcriptional events.

### Symbiotic Cell Differentiation and Bacteroid Differentiation Are Key Steps for the Establishment of the “Nodule-Specific Transcriptome”

**Figure 7:**
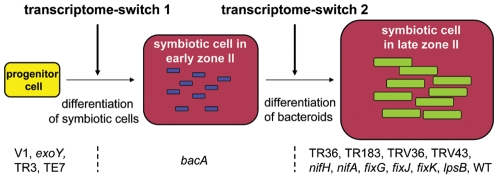
Two transcriptome-switches in nodule formation. Progenitor cells (yellow) in the primordium or meristem differentiate to large, endoreduplicated and infected symbiotic cells (red) thus activating the first transcriptome-switch. The V1, *exoY*, TR3 and TE7 mutants are blocked before this point. The rhizobia in symbiotic cells (blue) differentiate to large, endoreduplicated bacteroids (green) activating the second transcriptome-switch. The *bacA* mutant is blocked before this point but after the first switch. The mutants TR36, TR183, TRV36, TRV43, *nifH*, *nifA*, *fixG*, *fixJ*, *fixK* and *lpsB* pass, similarly to the WT, the second switch.

The correspondence of the histological, cytological and transcriptome data suggests that symbiotic cell differentiation and bacteroid differentiation are key steps for the establishment of the “nodule-specific transcriptome” (Figure 7). The correlation between the differentiation of symbiotic nodule cells and the first wave of nodule-specific gene activation suggests that the differentiation of the symbiotic cells constitutes a signal necessary for this transcriptome switch. One possible mechanism for this switch could involve endoreduplication (which drives the differentiation of symbiotic cells) directly in the regulation of gene expression, for example via an epigenetic control involving chromatin modifications. In agreement with this proposition, we have observed that, in wild type nodules, endoreduplication is initiated at the early stage when the first transcriptome wave takes place [5] and that the incipient nodules showed the presence of enlarged and infected symbiotic cells. Alternatively, the infection of symbiotic cells with rhizobia from infection threads and the formation of symbiosomes may be the trigger for this first transcriptome wave. Furthermore, the correlation between the presence of elongated, terminally differentiated bacteroids in symbiotic cells and the second transcriptome wave in nodules suggests that the differentiation of the bacteroids constitutes a signal for the execution of this second transcriptome-switch. Bacteroid differentiation involves their endoreduplication mediated elongation and is, also, associated with cell surface modifications [7], [63], [64]. Notably, the cell surface lipopolysaccharides are chemically modified. It is possible that these chemical modifications are recognized by plant receptors with subsequent activation of the second transcriptome-switch.

In conclusion, our results pinpoint new levels of communication between the two symbiotic partners. A challenge for the future will be to identify the molecular bases of these cell differentiation signals and the signal transduction pathways which lead to the gene activation that we have described.

## Materials and Methods

### *Sinorhizobium meliloti* Strains and *Medicago truncatula* Lines

The *M. truncatula* R108 or Jemalong J5 lines and the *S. meliloti* strains Sm41, Sm1021 or Sm2011 constituted the plant and bacterial genetic backgrounds of the mutants used in this study (Table S1). To exclude transcriptome variations due to the genetic background, the different wild type bacterial and plant lines were included in the study. The mutants covered a wide range of phenotypes, all of which displayed arrested nodule development. For the bacterial *bacA*, *exoY*, *lpsB* and *nif*/*fix* mutants, phenotypic descriptions are available [6], [18]–[21], [23], [24]. The *M. truncatula* Fix^−^ mutant TE7 has also been described before [22]. Four other *M. truncatula* Fix^−^ mutants, TR3, TR36, TR183 and TRV43, are representatives of 4 distinct complementation groups [65], [66] and TR3 and TE7 were found to be allelic mutants [67]. The TRV36 mutant was not assigned to any complementation group but is likely distinct because it forms fewer nodules and is also affected in root growth (data not shown). Except for the TE7 mutant, the Fix^−^ phenotype of the other mutants has not been further detailed. We, thus, established a phenotypic description of all the mutants used in this study to provide a framework for interpreting transcriptome results.

### Plant Growth and Nodulation

All bacterial strains were transformed via triparental conjugation with plasmid pXLGD4 carrying a constitutively expressed *lacZ* gene [68]. Plants were cultivated on perlite substrate humidified with SolI (http://www.isv.cnrs-gif.fr/embo01/manuels/pdf/module1.pdf). To obtain nodules, one week old seedlings were inoculated with bacterial suspensions at OD_600_ = 0.1. Nodules were harvested between 15 and 21 dpi. For the nodulation kinetics, the R108 wild type plants were cultivated with *S. meliloti* Sm41 in an aeroponic system (http://www.isv.cnrs-gif.fr/embo01/manuels/pdf/module1.pdf) using the same growth medium and rhizobial inoculation system as above. Nodules were harvested at 0, 2, 4, 6, 7, 8, 10, 13, 20, 29 dpi. From 0 to 6 dpi, no macroscopically visible signs of nodule development were detectable and samples were taken from root pieces in the nodulation competent zone (from the root tip to the region with mature root hairs). From 7, 8 and 10 dpi, clearly evident and macroscopically distinguishable white incipient nodules were collected. From 13 dpi on, the collected nodules were differentiated with zonation and with a pink colour indicative of nitrogen fixation.

### Histological Methods

Sections (7 µm) of technovit embedded nodules were stained with toluidine blue [3]. Thick sections (70 µm) of agarose embedded nodules were stained for *lacZ*-encoded β-galactosidase activity [69]. Sections were observed and photographed with a Leica DMI 6000B inverted microscope. Bacteroids were purified from nodules, stained with DAPI and observed by fluorescence microscopy as described [7].

*In situ* hybridizations with S^35^-labeled radioactive probes were done as described [70]. RNA probes for the *SPP* gene (MtGI accession number TC95075) were synthesized by *in vitro* transcription of PCR products obtained with gene specific oligos GCGGATGGTATGCTTTAAAG (forward primer) and CTTAAAATATTGGGGTTGCT (reverse primer) resulting in a 367 bp fragment, internal to the coding sequence. For the antisense probe, the reverse primer sequence was preceded with the T7 promoter sequence GAATTGTAATACGACTCACTATAGGGC. For synthesis of the control, sense probe, the forward gene specific primer was coupled to the T7 promoter sequence.

For immuno-localization of the ER with the ER-specific KDEL-antibody (MAC 256, abcam, Cambridge, UK), 7 µm longitudinal sections of fixed and paraffin imbedded nodules [70] were treated as follows: incubation in 0.1 M glycine, 0.1 M ammonium chloride in MTSB (50 mM Pipes, 5 mM EGTA, 5 mM MgSO_4_; pH 7.0) (15 min); washing with MTSB (5×10 min); washing with MTSB/0.1% triton (6×10 min); blocking in 3% BSA in MTSB/0.1% triton (1 h); incubation with primary antibody at 1/250 dilution (6 h); washing with MTSB/0.1% triton (8×10 min); incubation with secondary antibody anti-rat IgG-Alexa fluor 488 (Invitrogen) at 1∶300 dilution in 3% BSA in MTSB/0.1% triton (3 h); washing with MTSB/0.1% triton (8×12 min); washing with MTSB (5×10 min). Imaging was performed with a confocal laser scanning microscope TCS SP2 (Leica).

Flow cytometry measurements of ploidy levels in nodule nuclei were performed as described [4]. For each measurement, 6 nodules from 3 plants were pooled and each measurement was repeated 3 times. This sampling method avoided any eventual heterogeneity among individual nodules. The mean values of the repetitions were calculated. The fraction of nuclei at ploidy levels from 2C to 64C were expressed as a % of the total number of nuclei measured and the data were analyzed by hierarchical clustering with the Cluster and Treeview software [71]. An endoreduplication index expressing, specifically, the level of 16C, 32C and 64C endoreduplicated nuclei was calculated according to the formula (% 16C nuclei)*3+(% 32C nuclei)*4+(% 64C nuclei)*5.

### Microarrays

An initial, small EST collection of 389 clones from a *M. truncatula* R108 young nodule cDNA library [72] was extended with a total of 3459 new sequences (accession n° DY615432 to DY618499). Using BLASTN against a local database of the sequences and the *M. truncatula* EST collection at MtGI, these sequences were grouped into 2366 unigenes (Table S4). 525 cDNA were singletons corresponding to newly discovered transcripts.

For the production of custom microarrays, representative clones (probes) of the 2366 identified unigenes were amplified by PCR using vector-specific primers. The probes were spotted on glass slides (GAPS II, Corning) according to [73] with a Genetac G3 arrayer (Genomic Solution, Ann Arbor, MI, U.S.A.). Although the resulting microarrays cover the transcriptome of *M. truncatula* to a lesser extent than microarrays used in other studies [8], [10]–[13], our microarrays are nevertheless appropriate and well adapted to our studies since they should be strongly enriched in probes that represent nodule-enhanced genes given that these probes were isolated from a nodule cDNA library. Moreover, our objectives were to have a set of genes sufficiently large enough to dissect the nodulation process in transcriptional stages rather than to have a comprehensive view of the transcriptome.

Total RNA extractions from harvested materials were done with the Qiagen RNeasy Mini kit (Qiagen). 25 µg total RNA were used for sample labeling with Cy5 using the SuperScript™ Indirect cDNA Labeling System (Invitrogen) according to manufacturer's recommendations.

Dual label hybridizations on the microarrays were performed with a reference sample that was identical for all the hybridizations in this study. The reference consisted of an equimolar mix of PCR products of all the clones present on the microarrays. Labeling with Cy3 was done using the Megaprime kit (Amersham) according to the manufacturer's instructions.

Hybridizations with a mixture of the Cy5-labelled sample and the Cy3-labelled reference, image acquisition and spot intensity measurements were done as previously described [73].

The total hybridization intensity in the Cy5 channel for the root samples was much lower than for nodule samples. This is related to the nature of the custom microarrays which are printed with probes from a nodule cDNA library. These probes represent only a small part of the genome. The gene *mtc27*, which is commonly used as a constitutive control in roots and nodules for expression studies in *Medicago*, was used to identify the genes on the microarrays that that show low variation. The *mtc27* gene was spotted 12 times over the entire surface of the array on the slide. Comparison with *mtc27* allowed the identification of genes the expression of which was invariant among the samples and conditions studied. 1500 genes were selected and were used to normalize the complete data set. The ratio of Cy5/Cy3 intensities of each gene was normalized using the median of these 1500 genes selected for the normalization procedure. All of gene expression data were deposited in the ArrayExpress Database (www.ebi.ac.uk/arrayexpress) under accession number E-MEXP-1987.

To identify the genes that were differentially expressed during wild type nodule formation, the expression levels of the genes were compared, pair-wise, with a *t*-test. Genes for which the levels of expression were significantly different (*p*<0.01) in at least one of the 3 comparisons (roots versus incipient nodule, root versus nodule and incipient nodule versus nodule) were retained. Hierarchical cluster analysis, PCA and the generation of heat maps was done with the MultiExperiment Viewer software package [74] or Spotfire Decision Site Software (TIBCO). To permit visualization of statistical groups, as in Figure 4C, expression levels were transformed to integer values (1, 2 or 3) reflecting the strength of expression and the statistically supported difference in the expression level (the “Digital Genes” tool in the MultiExperiment Viewer software package).

### RT-qPCR

For the wild type developmental time course experiment, uninoculated R108 roots and immature (8 dpi) and mature nodules (15 dpi), formed after inoculation with *S. meliloti* Sm41, were sampled. For the experiment with the nodulation mutants, roots from J5 wild type, nodules (21 dpi) from TR3, TE7, TR36 and J5 wild type induced by wild type Sm1021 and nodules from J5 wild type induced by Sm1021*bacA* were collected. Three biological replicates were performed. Total RNA was extracted, using the RNeasy Plant Mini Kit (Qiagen), and treated with RNase-free DNase I to remove traces of genomic DNA. Removal was verified by PCR. First-strand cDNA was synthesized from 5 µg of total RNA using the Superscript® II first-strand synthesis system (Invitrogen). Primer design was performed using the Primer3 software (http://frodo.wi.mit.edu/cgi-bin/primer3/primer3_www.cgi). The primer sequences are listed in Table S5. Real-time RT-PCR reactions were performed using the LightCycler® FastStart DNA Master^PLUS^ SYBR Green I kit (Roche Mannheim, Germany) on a Roche LightCycler® 1.5 instrument. Conditions were 95°C for 10 min, 45 cycles at 95°C for 5 s, 60°C for 5 s and 72°C for 15 s. The specificity of the PCR amplification was confirmed by the analysis of the corresponding dissociation curves (55°C to 95°C). Crossing points (CP) for each transcript were determined with the LightCycler software. CP is the PCR cycle at which the fluorescence rises above the background. The relative expression levels were calculated according to the method described by [75] which is based upon CP values that are corrected for the amplification efficiency for each primer pair. The reference transcript (constitutive control) for normalization of input cDNA was Histone H3-like TC106487. Fold changes were calculated relative to the sample with the lowest value being arbitrarily designated as having a value of 1.

## Supporting Information

Figure S1 Nodule structure and infection in mutants. Semi-thin longitudinal nodule sections were stained with toluidine blue and observed by light microscopy. (A) TR183-Sm1021; (B) J5-Sm1021*lpsB*; (C) J5-Sm1021*nifA*; (D) TR36-Sm1021; (E) TRV36-Sm1021. (Left panels) Tissue organisation of nodules. Bars equal 200 µm. (Middle panels) Enlargement of the central area of the nodules showing the presence of differentiated symbiotic cells. Bars equal 50 µm. (Right panels) Images of symbiotic cells showing the structure of the intracellular bacteria. Bars equal 50 µm.
(8.69 MB TIF)

Figure S2 Infection of nodule cells in wild type and mutant nodules. Plant roots were infected with rhizobia expressing, constitutively, the *lacZ* gene from the plasmid pXLGD4 and thick nodule sections were stained for *lacZ* encoded β-galactosidase activity (blue colour). (A) J5-Sm1021 Wild type; (B) TRV43-Sm1021; (C) TRV36-Sm1021; (D) TR183-Sm1021; (E) TR36-Sm1021. (Left panels) Infection pattern of a whole nodule. Bars equal 200 µm. (Middle panels) Enlargement of the central area of the nodules showing the presence or absence of differentiated symbiotic cells. Bars equal 50 µm. (Right panels) Enlargement of the senescence zone of nodules showing the continuous growth of an infection thread network as well as senescent cells occupied with saprophytic rhizobia. Bars equal 50 µm.
(7.95 MB TIF)

Figure S3 Infection of nodule cells in wild type and mutant nodules. Plant roots were infected with rhizobia expressing, constitutively, the *lacZ* gene from the plasmid pXLGD4 and thick nodule sections were stained for *lacZ* encoded β-galactosidase activity (blue colour). (A) J5-Sm1021*nifA*; (B) J5-Sm1021*lpsB*; (C) J5-Sm1021*bacA*; (D) TR3-Sm1021; (E) V1-Sm1021. (Left panels) Infection pattern of a whole nodule. Bars equal 200 µm. (Middle panels) Enlargement of the central area of the nodules showing the presence or absence of differentiated symbiotic cells. Bars equal 50 µm. (Right panels) Enlargement of the senescence zone of nodules showing the continuous growth of an infection thread network as well as senescent cells occupied with saprophytic rhizobia. Bars equal 50 µm.
(7.31 MB TIF)

Figure S4 Structure of incipient and mature wild type nodules. R108 *M. truncatula* plants were inoculated with *S. meliloti* Sm41. Semi-thin longitudinal sections of an incipient, 7 dpi (A–E) and a mature, 13 dpi (F–H) nodule, stained with toluidine blue, are shown. (A) Tissue organisation of an incipient nodule with a meristem (I) and an infection zone (II). (B) Schematic illustration of the nodule shown in (A). (C) Enlargement of the meristem and infection zone of the nodule shown in (A). (D) Enlargement of the root proximal zone of the nodule shown in (A), containing large infected cells. (E) Enlargement of infected cells of the nodule shown in (A) showing the presence of not yet differentiated bacteria. (F) Mature, nitrogen-fixing nodule displaying nodule zones I, II and III. (G) Schematic presentation of the nodule shown in (F). (H) Enlargement of symbiotic cells from the nodule shown in (F). Differentiated, elongated bacteroids are visible in these cells. Scale bars: 200 µm (A and F); 50 µm (C–E and H).
(8.42 MB TIF)

Table S1 Bacterial strains and plant lines used in this study.
(0.10 MB DOC)

Table S2 List of 520 differentially expressed genes identified in this study. The first column is the order of genes in the heat maps of Figure 4B, 4C and 6B. The second column indicates the expression profile to which the gene belongs (Figure 4). The third column indicates the induction factor for the gene between the condition with the lowest and the highest expression. The clone number is an identification number for laboratory use. The next columns indicate GenBank and MtGI accession numbers. The last column is an annotation of the gene corresponding to the clone. A colour-coded category of the encoded proteins is provided: in yellow are secretory proteins, in blue are transmembrane proteins, in green are proteins of the secretory system, in orange are stress responsive proteins, light blue is for protein synthesis, and in pink are cell division proteins.
(0.13 MB PDF)

Table S3 List of clones for genes that are induced in all the mutants that were analyzed in this study. The cluster, clone number, accession number and annotation are indicated.
(0.06 MB XLS)

Table S4 List of the clones present on the custom microarrays. Clones were isolated from a *M. truncatula* R108 nodule cDNA library. Clone identification numbers for internal use are given together with GenBank accession numbers at http://www.ncbi.nlm.nih.gov/entrez/ and MtGI accession numbers at http://compbio.dfci.harvard.edu/tgi/.
(0.10 MB PDF)

Table S5 Primer list used for RT-qPCR experiments shown in Figure 4E and Figure 6C.
(0.08 MB PDF)
