# Supplementary figures and images for: Differentiation of Symbiotic Cells and Endosymbionts in Medicago truncatula Nodulation Are Coupled to Two Transcriptome-Switches
Source: PLoS One. 2010 Mar 4;5(3):e9519. doi: 10.1371/journal.pone.0009519 (PMC2832008; doi:10.1371/journal.pone.0009519)

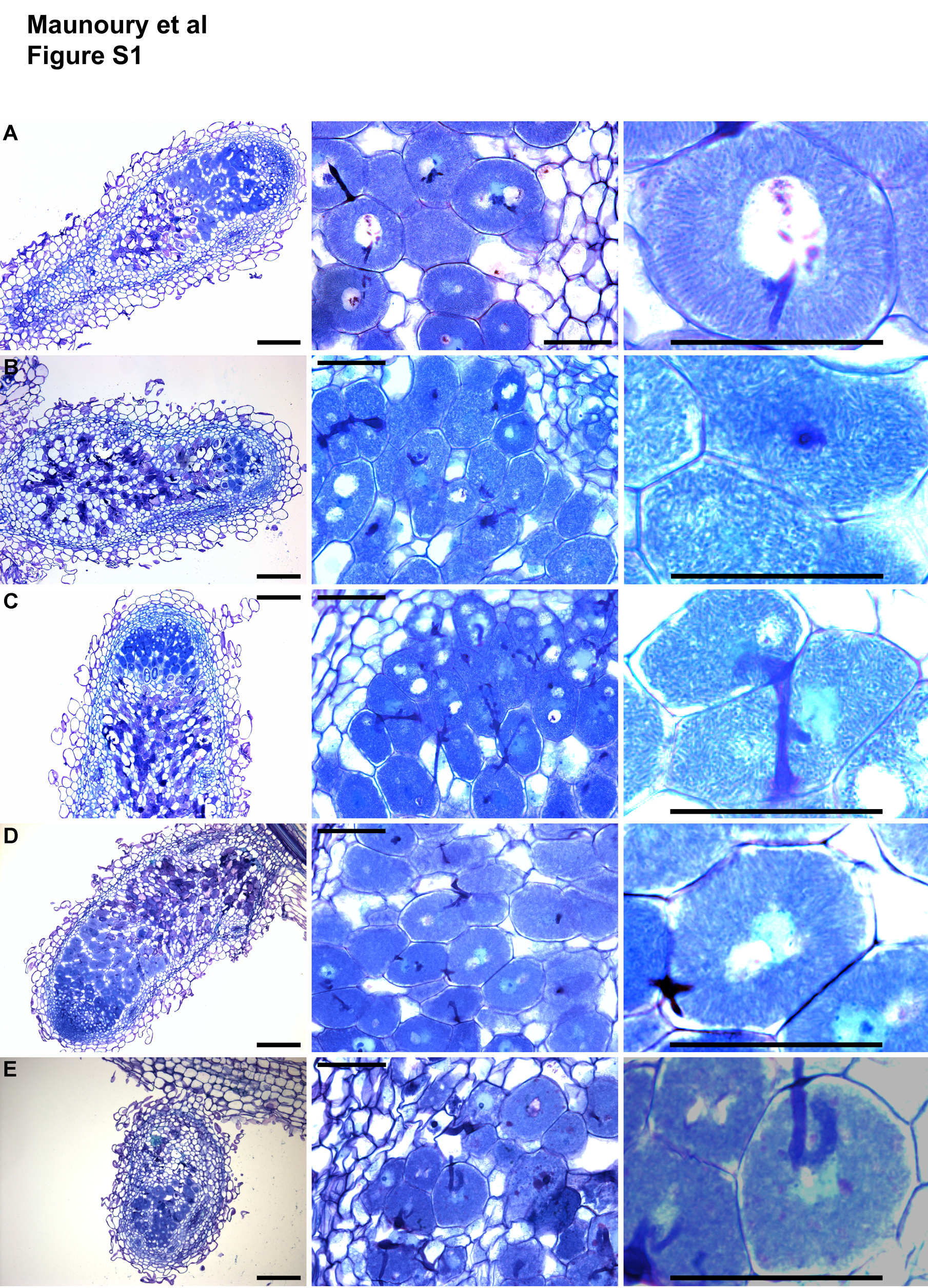

Supplement: Figure S1 — Nodule structure and infection in mutants. Semi-thin longitudinal nodule sections were stained with toluidine blue and observed by light microscopy. (A) TR183-Sm1021; (B) J5-Sm1021lpsB; (C) J5-Sm1021nifA; (D) TR36-Sm1021; (E) TRV36-Sm1021. (Left panels) Tissue organisation of nodules. Bars equal 200 µm. (Middle panels) Enlargement of the central area of the nodules showing the presence of differentiated symbiotic cells. Bars equal 50 µm. (Right panels) Images of symbiotic cells showing the structure of the intracellular bacteria. Bars equal 50 µm. (8.69 MB TIF) [file pone.0009519.s001.tif]

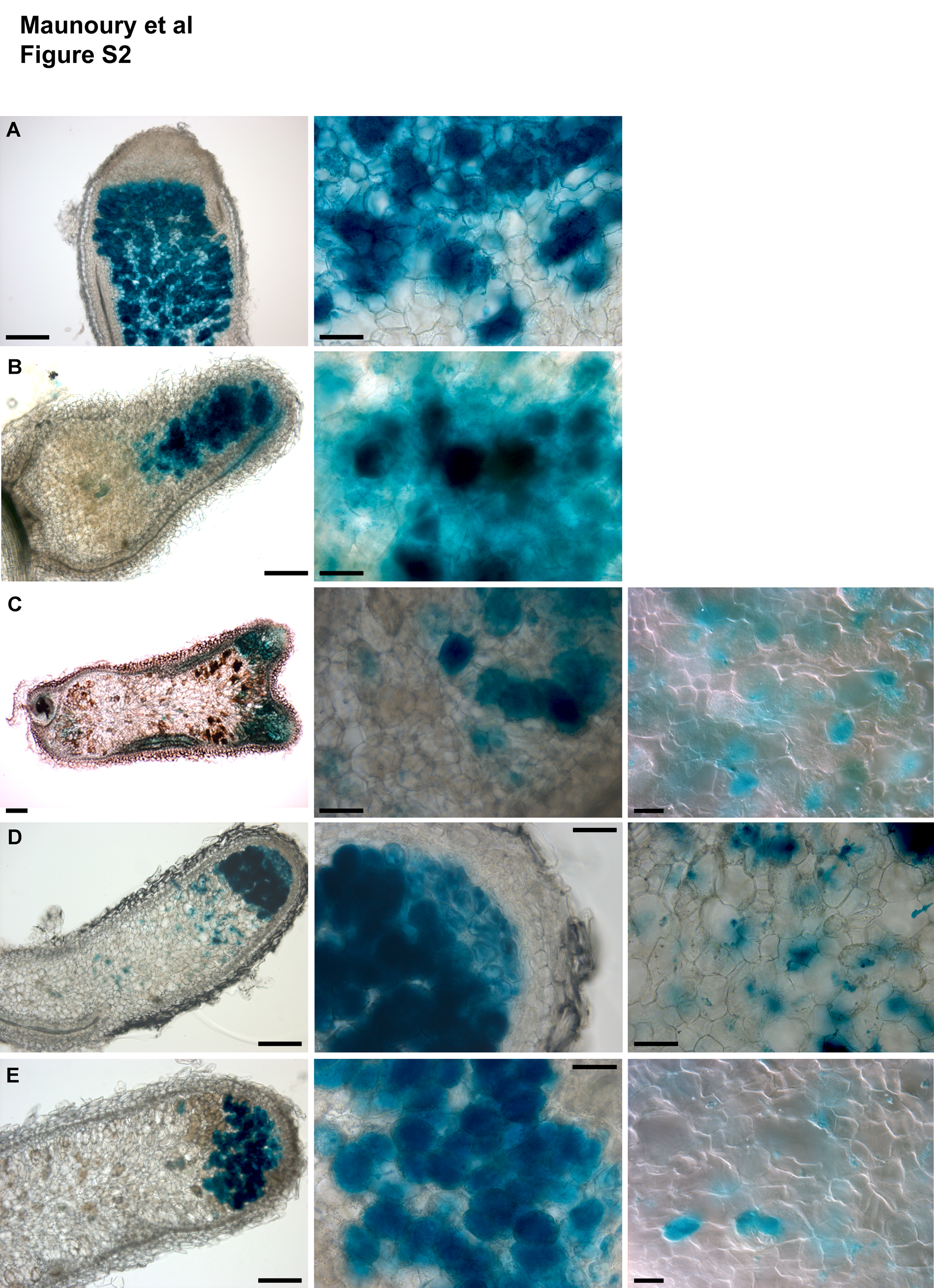

Supplement: Figure S2 — Infection of nodule cells in wild type and mutant nodules. Plant roots were infected with rhizobia expressing, constitutively, the lacZ gene from the plasmid pXLGD4 and thick nodule sections were stained for lacZ encoded β-galactosidase activity (blue colour). (A) J5-Sm1021 Wild type; (B) TRV43-Sm1021; (C) TRV36-Sm1021; (D) TR183-Sm1021; (E) TR36-Sm1021. (Left panels) Infection pattern of a whole nodule. Bars equal 200 µm. (Middle panels) Enlargement of the central area of the nodules showing the presence or absence of differentiated symbiotic cells. Bars equal 50 µm. (Right panels) Enlargement of the senescence zone of nodules showing the continuous growth of an infection thread network as well as senescent cells occupied with saprophytic rhizobia. Bars equal 50 µm. (7.95 MB TIF) [file pone.0009519.s002.tif]

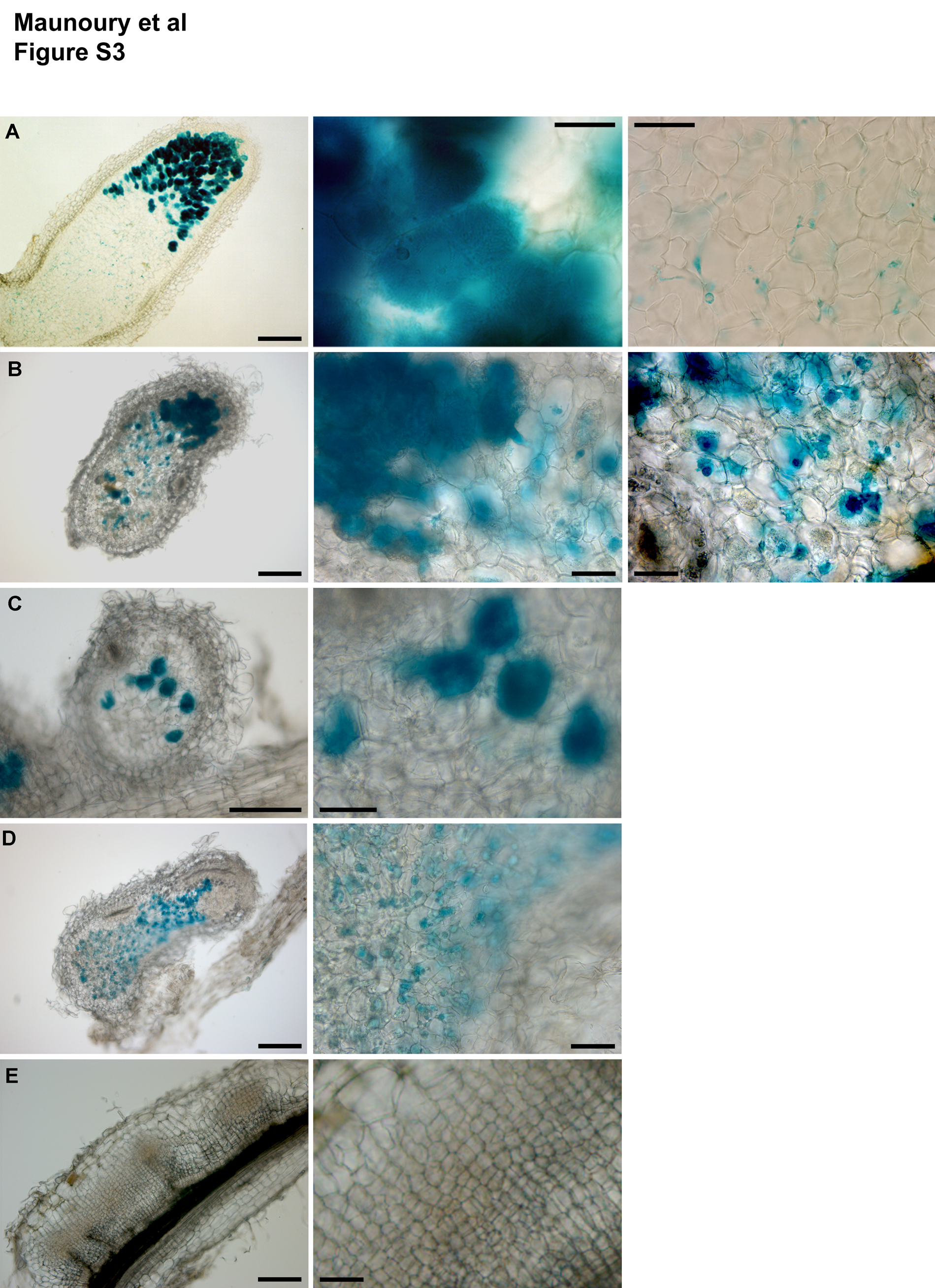

Supplement: Figure S3 — Infection of nodule cells in wild type and mutant nodules. Plant roots were infected with rhizobia expressing, constitutively, the lacZ gene from the plasmid pXLGD4 and thick nodule sections were stained for lacZ encoded β-galactosidase activity (blue colour). (A) J5-Sm1021nifA; (B) J5-Sm1021lpsB; (C) J5-Sm1021bacA; (D) TR3-Sm1021; (E) V1-Sm1021. (Left panels) Infection pattern of a whole nodule. Bars equal 200 µm. (Middle panels) Enlargement of the central area of the nodules showing the presence or absence of differentiated symbiotic cells. Bars equal 50 µm. (Right panels) Enlargement of the senescence zone of nodules showing the continuous growth of an infection thread network as well as senescent cells occupied with saprophytic rhizobia. Bars equal 50 µm. (7.31 MB TIF) [file pone.0009519.s003.tif]

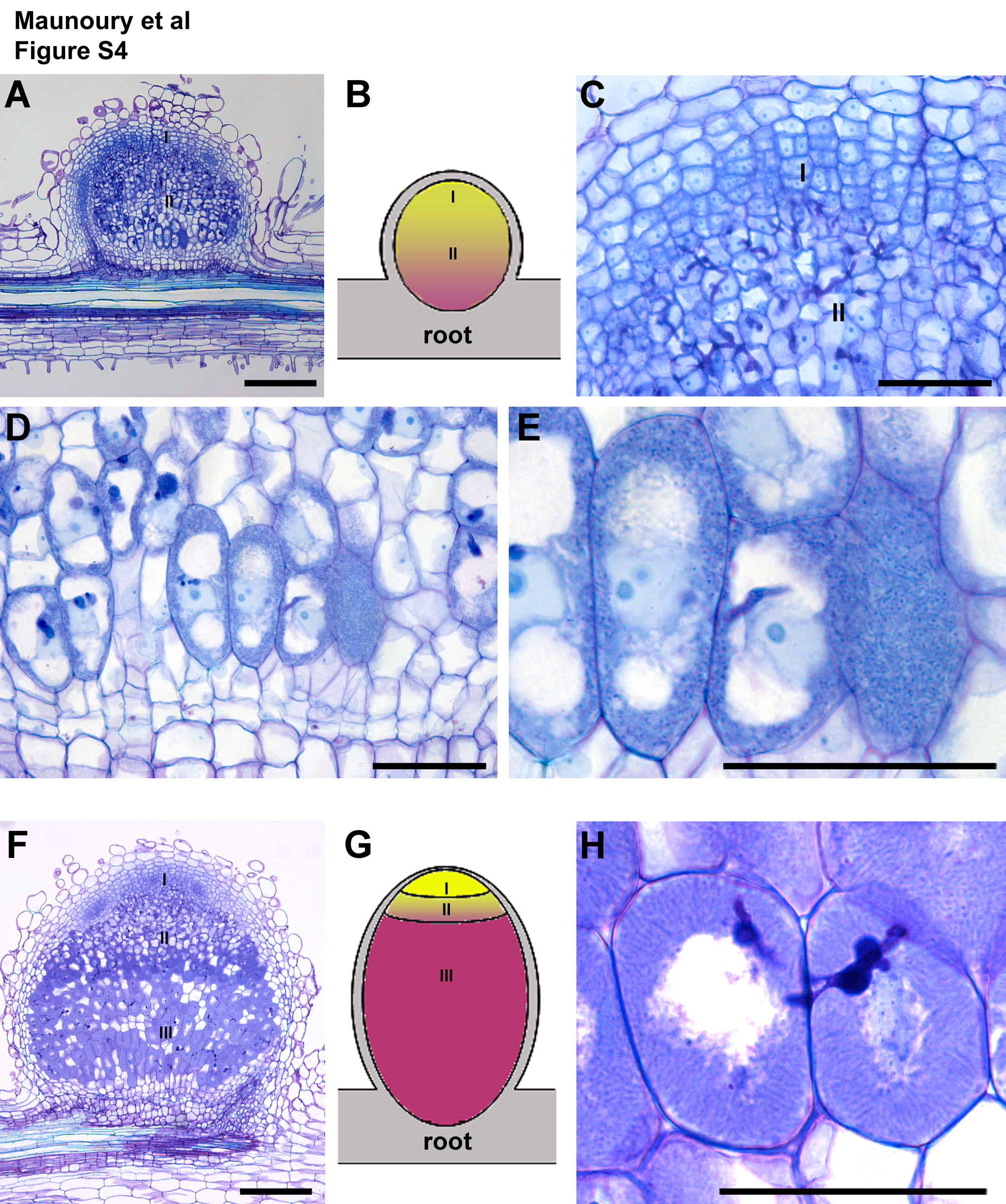

Supplement: Figure S4 — Structure of incipient and mature wild type nodules. R108 M. truncatula plants were inoculated with S. meliloti Sm41. Semi-thin longitudinal sections of an incipient, 7 dpi (A–E) and a mature, 13 dpi (F–H) nodule, stained with toluidine blue, are shown. (A) Tissue organisation of an incipient nodule with a meristem (I) and an infection zone (II). (B) Schematic illustration of the nodule shown in (A). (C) Enlargement of the meristem and infection zone of the nodule shown in (A). (D) Enlargement of the root proximal zone of the nodule shown in (A), containing large infected cells. (E) Enlargement of infected cells of the nodule shown in (A) showing the presence of not yet differentiated bacteria. (F) Mature, nitrogen-fixing nodule displaying nodule zones I, II and III. (G) Schematic presentation of the nodule shown in (F). (H) Enlargement of symbiotic cells from the nodule shown in (F). Differentiated, elongated bacteroids are visible in these cells. Scale bars: 200 µm (A and F); 50 µm (C–E and H). (8.42 MB TIF) [file pone.0009519.s004.tif]
