# Supplementary material for: Differentiation of Symbiotic Cells and Endosymbionts in Medicago truncatula Nodulation Are Coupled to Two Transcriptome-Switches
Source: PLoS One. 2010 Mar 4;5(3):e9519. doi: 10.1371/journal.pone.0009519 (PMC2832008; doi:10.1371/journal.pone.0009519)
Supplement: Table S1 — Bacterial strains and plant lines used in this study. (0.10 MB DOC) [file pone.0009519.s005.doc]

| ***Sinorhizobium meliloti* and *Medicago truncatula* strains** | | | | | | | |
| --- | --- | --- | --- | --- | --- | --- | --- |
|  | | | | | | | |
| ***S. meliloti* genetic backgrounds** | | | | | | | |
| **Strain** | | **Relevant features** | | | | References | |
| Sm41 | | Nodulates *M. truncatula* R108 efficiently  Nodulates *M. truncatula* J5 inefficiently (Fix^-^) | | | | [1] | |
| Sm1021 | | Nodulates *M. truncatula* R108 and J5 efficiently | | | | [2] | |
| Sm2011 | | Nodulates *M. truncatula* R108 and J5 efficiently | | | | [3] | |
| ***S. meliloti* mutants** | | | | | | | |
| **Mutated gene** | **Background** | | **Strain N°** | | **Gene function** | | **Reference** |
| *exoY* | Sm2011 | | Sm0540 | | Galactosyl transferase; EPS and KPS synthesis | | [4] |
| *lpsB* | Sm2011 | | Sm6963 | | Mannosyl transferase; LPS synthesis | | [5] |
| *bacA* | Sm1021 | | Sm8386 | | Transport protein | | [6] |
| *nifH* | Sm2011 | | GMI296 | | Nitrogenase subunit | | J. Batut |
| *fixG* | Sm2011 | | GMI394 | | Fe-S membrane protein | | [7] |
| *fixJ* | Sm2011 | | GMI347 | | Transcriptional regulator | | [7] |
| *fixK* | Sm2011 | | GMI942 | | Transcriptional regulator | | [8] |
| *nifA* | Sm2011 | | GMI5601 | | Transcriptional regulator | | [7] |
| ***M. truncatula* genetic backgrounds** | | | | | | | |
| **Line** | | | | **Reference** | | | |
| R108 | | | | [9] | | | |
| JemalongJ5 | | | | [10] | | | |
| ***M. truncatula* mutants** | | | | | | | |
| **Line** | | **Background/mutated gene** | | | **Reference** | | |
| V1 | | R108/ undetermined | | | [11] | | |
| TE7 | | J5*/ Mtsym1=Mtsym17* | | | [12,13] | | |
| TR3 | | J5/ *Mtsym17=Mtsym1* | | | [10,13,14] | | |
| TR36 | | J5/ *Mt sym18* | | | [10,14] | | |
| TRV36 | | J5/ undetermined | | | unpublished. | | |
| TR183 | | J5/ *Mtsym19* | | | [10,14] | | |
| TRV43 | | J5/*Mtsym20* | | | [14] | | |

**References**

1. Kondorosi E, Banfalvi Z, Kondorosi A (1984) Physical and genetic analysis of a symbiotic region of *Rhizobium meliloti*: identification of nodulation genes. Mol Gen Genet 193: 445-452.

2. Meade HM, Long SR, Ruvkun GB, Brown SE, Ausubel FM (1982) Physical and genetic characterization of symbiotic and auxotrophic mutants of *Rhizobium meliloti* induced by transposon Tn*5* mutagenesis. J Bacteriol 149: 114-122.

3. Rosenberg C, Boistard P, Denarie J, Casse-Delbart F (1981) Genes controlling early and late functions in symbiosis are located on a megaplasmid in *Rhizobium meliloti*. Mol Gen Genet 184: 326-333.

4. Müller P, Keller M, Weng WM, Quandt J, Arnold W, et al. (1993) Genetic analysis of the *Rhizobium meliloti* *exoYFQ* operon: ExoY is homologous to sugar transferases and ExoQ represents a transmembrane protein. Mol Plant-Microbe Interact 6: 55-65.

5. Niehaus K, Becker A (1998) The role of microbial surface polysaccharides in the Rhizobium-legume interaction. Subcell Biochem 29: 73-116.

6. Glazebrook J, Ichige A, Walker GC (1993) A *Rhizobium meliloti* homolog of the *Escherichia coli* peptide-antibiotic transport protein SbmA is essential for bacteroid development. Genes Dev 7: 1485-1497.

7. David M, Daveran ML, Batut J, Dedieu A, Domergue O, et al. (1988) Cascade regulation of *nif* gene expression in *Rhizobium meliloti*. Cell 54: 671-683.

8. Foussard M, Garnerone AM, Ni F, Soupene E, Boistard P, et al. (1997) Negative autoregulation of the *Rhizobium meliloti fixK* gene is indirect and requires a newly identified regulator, FixT. Mol Microbiol 25: 27-37.

9. Hoffmann B, Trinh TH, Leung J, Kondorosi A, Kondorosi E (1997) A new *Medicago truncatula* line with superior in vitro regeneration, transformation, and symbiotic properties isolated through cell culture selection. Mol Plant-Microbe Interact 10: 307-315.

10. Sagan M, Morandi D, Tarenghi E, Duc G (1995) Selection of nodulation and mycorrhizal mutants in the model-plant *Medicago truncatula* (*Gaertn.*) after gamma-ray mutagenesis. Plant Sci 111: 63-71.

11. Mergaert P, Nikovics K, Kelemen Z, Maunoury N, Vaubert D, et al. (2003) A novel family in *Medicago truncatula* consisting of more than 300 nodule-specific genes coding for small, secreted polypeptides with conserved cysteine motifs. Plant Physiol 132: 161-173.

12. Bénaben V, Duc G, Lefebvre V, Huguet T (1995) TE7, an inefficient symbiotic mutant of *Medicago truncatula Gaertn.* cv Jemalong. Plant Physiol 107: 53-62.

13. Sagan M, De Larembergue H, Morandi D (1998) Genetic analysis of symbiosis mutants in *Medicago truncatula*. In: Elmerich C, Kondorosi A, Newton WE, editors. Biological Nitrogen Fixation for the 21^st^ Century: Kluwer. pp. 317-318.

14. Morandi D, Prado E, Sagan M, Duc G (2005) Characterisation of new symbiotic *Medicago truncatula* (*Gaertn.*) mutants, and phenotypic or genotypic complementary information on previously described mutants. Mycorrhiza 15: 283-289.
