# Supplementary material for: Differentiation of Symbiotic Cells and Endosymbionts in Medicago truncatula Nodulation Are Coupled to Two Transcriptome-Switches
Source: PLoS One. 2010 Mar 4;5(3):e9519. doi: 10.1371/journal.pone.0009519 (PMC2832008; doi:10.1371/journal.pone.0009519)
Supplement: Table S2 — List of 520 differentially expressed genes identified in this study. The first column is the order of genes in the heat maps of Figure 4B, 4C and 6B. The second column indicates the expression profile to which the gene belongs (Figure 4). The third column indicates the induction factor for the gene between the condition with the lowest and the highest expression. The clone number is an identification number for laboratory use. The next columns indicate GenBank and MtGI accession numbers. The last column is an annotation of the gene corresponding to the clone. A colour-coded category of the encoded proteins is provided: in yellow are secretory proteins, in blue are transmembrane proteins, in green are proteins of the secretory system, in orange are stress responsive proteins, light blue is for protein synthesis, and in pink are cell division proteins. (0.13 MB PDF) [file pone.0009519.s006.pdf]

tree n°	expression profile	induction factor	clone n°	GenBank accession	MtGI accession	annotation
1	profile 1	3,174548684	AC0073	AJ388714	TC94210	class III peroxidase
2	profile 1	3,113332709	AC1366	DY615989	TC93959	class I chitinase
3	profile 1	4,142124409	AC0589	DY615508	TC100398	chalcone reductase
4	profile 1	3,293078828	AC4521	DY618225	TC100399	chalcone reductase
5	profile 1	2,763481866	AC2173	DY616637	TC94379	endoxyloglucan transferase; cell wall biosynthesis
6	profile 1	2,381882534	AC0320	AJ388949	TC106716	3-deoxy-D-arabino-heptulosonate 7-phosphate synthase; aromatic amino acid family biosynthesis
7	profile 1	2,640342419	AC4011	DY617826	TC94783	protein of unknown function
8	profile 1	2,727792485	AC1668	DY616229	TC101355	N-hydroxycinnamoyl/benzoyltransferase; phytoalexin biosynthesis
9	profile 1	4,670527656	AC4532	DY618233	TC106619	aquaporin
10	profile 1	2,736543308	AC2320	DY616740	TC106642	cysteine proteinase
11	profile 1	2,165400925	AC0416	AJ389039	TC100146	polyubiquitin protein
12	profile 1	5,340956395	AC2248	DY616694	TC100291	aquaporin
13	profile 1	2,790428956	AC2703	DY617025	TC94604	arginine decarboxylase
14	profile 1	2,032468495	AC2153	DY616618	TC106701	protein of unknown function
15	profile 1	8,266523366	AC1414	DY616027	TC106536	chalcone synthase
16	profile 1	3,823504572	AC0662	DY615578	TC94281	isoflavone reductase
17	profile 1	4,924898619	AC2540	DY616902	singleton	isoflavone reductase
18	profile 1	2,637140889	AC4715	DY618371	TC94651	MYB transcription factor
19	profile 1	2,569288491	AC2663	DY616994	TC108442	protein of unknown function
20	profile 1	3,444317729	AC4171	DY617954	TC100215	protein of unknown function with Sec14p-like lipid-binding domain
21	profile 1	3,377020122	AC4841	DY618465	TC94571	protein of unknown function with DUF1645 domain
22	profile 1	1,851731693	AC1207	DY615867	TC95172	Ethylene-responsive transcription factor
23	profile 1	2,946113522	AC4864	DY618481	TC94944	protein of unknown function
24	profile 1	2,435018614	AC3026	DY617248	TC106726	protein of unknown function; B12D protein
25	profile 1	2,673564939	AC4245	DY618015	TC94676	Class III peroxidase
26	profile 1	4,502894407	AC2350	DY616765	TC97485	beta-glucan-elicitor receptor
27	profile 1	1,89086513	AC1461	DY616071	TC94230	hydroxyisourate hydrolase; glycosyl hydrolase family 1 protein
28	profile 1	1,894433222	AC3007	DY617236	TC106810	cellulose synthase
29	profile 1	4,551261155	AC4215	DY617986	TC93930	60S ribosomal protein L10
30	profile 1	5,522483514	AC1191	DY615854	TC108403	protease inhibitor

31	profile 1	3,841309937	AC0044	AJ388685	TC107101	isoflavone reductase
32	profile 1	3,345965354	AC1101	DY615780	TC100155	lipoxygenase
33	profile 1	4,162226616	AC3828	DY617683	TC94611	eukaryotic aspartyl protease
34	profile 1	3,073747616	AC4284	DY618045	TC106484	class III peroxidase
35	profile 1	4,402662817	AC1560	DY616156	TC100746	class III peroxidase
36	profile 1	4,400341435	AC1700	DY616252	TC100779	protein of unknown function with plastocyanin-like domain (copper binding protein)
37	profile 1	4,417545026	AC2725	DY617046	TC106753	protein of unknown function; DREPP plasma membrane polypeptide
38	profile 1	2,016419936	AC1159	DY615827	TC107600	lipase
39	profile 1	1,877274083	AC1741	DY616282	TC101289	protein of unknown function
40	profile 1	2,958864522	AC2315	DY616736	TC106503	protein of unknown function; protein with 4 ACT domain repeats
41	profile 1	2,542450632	AC3226	DY617344	TC100768	protein of unknown function; IQ calmodulin-binding motif
42	profile 1	3,398984724	AC3887	DY617723	singleton	no homology
43	profile 1	2,656858335	AC1541	DY616140	TC106316	plant invertase/pectin methylesterase inhibitor (PMEI)
44	profile 1	2,108181563	AC1562	DY616158	TC94661	signal peptidase I
45	profile 1	1,82994603	AC3744	DY617614	TC107320	Isoflavone reductase
46	profile 1	2,181402092	AC0768	DY615680	TC93944	protein of unknown function; late-embryogenesis protein
47	profile 1	3,709907936	AC2927	DY617198	TC100398	chalcone reductase
48	profile 1	3,429524479	AC0371	AJ388996	TC106667	phenylalanine ammonia-lyase (PAL)
49	profile 1	5,899128294	AC4292	DY618051	TC94347	class III peroxidase
50	profile 1	4,90075482	AC1202	DY615862	TC94347	class III peroxidase
51	profile 2	3,607386615	AC2044	DY616525	TC106656	beta-glucosidase; Glycosyl hydrolase family 1
52	profile 2	2,425738457	AC4622	DY618297	TC107222	brassinosteroid biosynthetic protein diminuto; cell elongation
53	profile 2	1,932263083	AC1612	DY616191	TC107365	glutaminyl-peptide cyclotransferase; protein post-translational modification
54	profile 2	1,989311802	AC1530	DY616131	TC100802	Protein of unknown function with DUF791 domain
55	profile 2	2,680067565	AC4716	DY618372	TC106540	class III peroxidase
56	profile 2	2,132690607	AC1121	DY615797	TC103583	dirigent-like protein; disease resistance response protein
57	profile 2	3,070246093	AC1174	DY615840	TC100478	serine protease; subtilisin-like proteinase
58	profile 2	5,353958163	AC3809	DY617667	TC100589	quinone oxidoreductase
59	profile 2	3,568896063	AC2749	DY617066	TC100519	UDP-glucosyltransferase; anthocyanidin-3-glucoside rhamnosyltransferase
60	profile 2	7,161939748	AC0143	AJ388780	TC100308	aquaporin
61	profile 2	2,349413382	AC0156	AJ388793	TC106425	L-ascorbate peroxidase
62	profile 2	2,66582142	AC1141	DY615814	TC94364	glutathione S-transferase
63	profile 2	2,01128208	AC1300	DY615953	TC106715	glycine/serine hydroxymethyltransferase; amino acid transport and metabolism
64	profile 2	4,177603903	AC4494	DY618204	TC100885	class III peroxidase
65	profile 2	6,674177139	AC3440	DY617449	TC106496	ripening related protein; Pathogenesis-related protein Bet v I family

66	profile 2	1,944179067	AC0547	DY615476	TC106633	3-hydroxy-3-methylglutaryl coenzyme a reductase (HMGR1)
67	profile 2	4,620355967	AC1326	DY615967	TC97494	aquaporin
68	profile 2	2,346663931	AC1018	DY615729	TC100483	calmodulin
69	profile 2	1,594109391	AC0378	AJ389002	TC94131	Ras-like Sar1 GTP binding protein; intracellular protein transport
70	profile 2	1,738898254	AC0406	AJ389029	TC100979	disease resistance protein-like protein MsR1; CC-NBS-LRR class
71	profile 2	1,444282997	AC0191	AJ388825	TC103840	protein of unknown function
72	profile 2	1,573173061	AC0274	AJ388905	TC106899	40S ribosomal protein S20
73	profile 2	1,408161876	AC0527	DY615458	TC107324	small G-protein ROP/RAC
74	profile 3	2,493032823	AC2134	DY616602	TC107303	bZIP transcription factor
75	profile 3	8,759055838	AC3794	DY617654	TC106600	auxin-repressed protein of unknown function
76	profile 3	2,469072633	AC4796	DY618428	TC107164	protein of unknown function
77	profile 3	2,044508588	AC0561	DY615487	TC94532	O-diphenol-O-methyl transferase
78	profile 3	3,465199359	AC3792	DY617652	TC106608	protein of unknown function
79	profile 3	1,718115821	AC1768	DY616304	singleton	peptidase; papain; cathepsin B-like cysteine proteinase
80	profile 3	2,208180964	AC1824	DY616353	TC94526	protein of unknown function
81	profile 3	1,981241886	AC1067	DY615761	TC100463	Cinnamyl alcohol dehydrogenase
82	profile 3	3,529538586	AC2351	DY616766	TC107254	protein of unknown function with CBS domain
83	profile 4	5,764768108	AC2456	DY616837	TC109032	MtN3; contains 7 transmembrane domains
84	profile 4	3,866165691	AC2566	DY616921	TC94341	cytochrome P450; isopenoid hydroxylase
85	profile 4	4,334194709	AC2383	DY616791	TC106707	protein disulfide-isomerase (PDI)
86	profile 4	2,392486036	AC4565	DY618259	TC94606	xyloglucan endotransglycosylase; cell growth
87	profile 4	2,016240434	AC1372	DY615995	TC109033	aurora kinase; regulation of mitosis; serine/threonine-specific protein kinase
88	profile 4	1,651238498	AC1147	DY615818	TC106512	60S ribosomal protein L39
89	profile 4	3,625190073	AC2113	DY616584	TC100408	tubulin alpha; cytoskeleton
90	profile 4	1,955636668	AC3099	DY617279	TC106356	mitochondrial heat shock 70 kDa protein; protein synthesis; protein folding
91	profile 4	2,566989359	AC0391	AJ389014	TC108193	histone H4
92	profile 4	2,52634809	AC1675	DY616234	TC107228	calmodulin-binding protein
93	profile 4	9,072096398	AC2751	DY617068	TC100767	L-asparaginase (conversion of asparagine to aspartate); nitrogen metabolism
94	profile 4	1,852903683	AC0405	AJ389028	TC108603	NADH-cytochrome b5 reductase
95	profile 4	2,811152845	AC1181	DY615847	singleton	GRP1G; EST corresponds to 3' untranslated
96	profile 4	1,681223343	AC1958	DY616463	TC112406	protein of unknown function
97	profile 4	5,293315948	AC2026	DY616512	TC106691	secreted protein; glutamine rich cell wall protein homologous to cotton fiber proteins E6
98	profile 4	2,193864035	AC3854	DY617701	TC104788	transcription factor; CCCH-type zinc finger

99	profile 4	2,714218506	AC1467	DY616076	TC101141	histone deacetylase HD2; repression of expression; associated with cell cycle progression
100	profile 4	2,189604286	AC1477	DY616085	singleton	RING-finger-containing ubiquitin ligase
101	profile 4	3,17221606	AC1114	DY615791	TC94539	60S ribosomal protein L6
102	profile 4	2,390893947	AC2771	DY617083	TC106375	60S ribosomal protein L7
103	profile 4	2,875493019	AC0310	AJ388940	TC106692	60S ribosomal protein L9
104	profile 4	2,177532824	AC0098	AJ388737	TC94402	40S ribosomal protein S13
105	profile 4	3,007846766	AC3782	DY617645	TC106902	40S ribosomal protein S15a
106	profile 4	2,576334085	AC0374	AJ388998	TC106741	40S ribosomal protein S24
107	profile 4	2,931844647	AC2647	DY616983	TC106794	T-complex protein 1 epsilon subunit (TCP-1-epsilon) (CCT-epsilon); subunit of cytosolic chaperonin; promote protein folding by using energy derived from ATP hydrolysis
108	profile 4	2,664960787	AC4226	DY617997	TC106794	T-complex protein 1 epsilon subunit (TCP-1-epsilon) (CCT-epsilon); subunit of cytosolic chaperonin; promote protein folding by using energy derived from ATP hydrolysis
109	profile 4	2,606753616	AC1422	DY616034	singleton	UDP-glucose glucosyltransferase
110	profile 4	1,528223937	AC2805	DY617112	TC95942	Uridine 5'-monophosphate synthase (UMP synthase); Pyrimidine metabolism
111	profile 4	5,85735412	AC0358	AJ388983	TC106640	ferritin, iron ion binding protein, iron homeostasis
112	profile 4	2,88207529	AC1815	DY616346	TC106674	cyclophilin; peptidylprolyl cis-trans isomerase (PPIase); PPIase accelerates protein folding by catalyzing the cis-trans isomerization of the peptide bonds preceding proline residues
113	profile 4	2,351303921	AC2025	DY616511	TC95495	RNA binding protein; contains a RRM domain found in proteins part of RNPs
114	profile 4	2,470543264	AC0042	AJ388684	TC94718	translation initiation factor 3 subunit 3 (eIF-3 gamma)
115	profile 4	2,527649397	AC3765	DY617632	TC107407	elongation factor 1-gamma
116	profile 4	4,005753699	AC4448	DY618172	TC100539	WD-40 repeat protein; signal transduction; expressed in nodule primordia & meristem
117	profile 4	2,521368707	AC1609	DY616190	TC95169	mitochondrial import receptor subunit TOM40; protein-mitochondrial targeting; mitochondrial outer membrane translocase complex
118	profile 4	2,639047991	AC4092	DY617889	TC107200	lysyl-tRNA synthetase
119	profile 4	2,499246529	AC4836	DY618462	TC100645	mevalonate disphosphate decarboxylase; mevalonate pathway
120	profile 4	5,079627361	AC4013	DY617828	TC107261	rip1; Secretory peroxidase; Class III of the plant heme-dependent peroxidase superfamily
121	profile 4	3,929664944	AC0043	AJ389056	TC106354	polyadenylate-binding protein, positive regulation of translation
122	profile 4	2,293330026	AC2731	DY617052	TC107591	endo-polygalacturonase
123	profile 4	9,101148097	AC2810	DY617117	TC106707	protein disulfide-isomerase precursor (PDI)
124	profile 4	2,843658241	AC0145	AJ388782	TC94384	sec61 translocase alpha subunit; ER protein involved in protein secretion

125	profile 4	1,722339367	AC0110	AJ388748	TC107342	ribosome biogenesis; WD repeat; mRNA processing; rRNA processing
126	profile 4	2,937769445	AC1261	DY615915	TC100411	Ribosomal protein L3
127	profile 4	2,39311315	AC1304	DY615956	TC94304	60S ribosomal protein L4
128	profile 4	2,801555409	AC0082	AJ388722	TC106773	60S ribosomal protein L5
129	profile 4	2,084549171	AC1411	DY616024	TC94398	26S proteasome subunit 4 AtRPT2a
130	profile 4	3,276002669	AC4859	DY618477	TC106368	tubulin beta chain (Beta tubulin)
131	profile 4	2,621008736	AC3411	DY617425	TC104645	UDP-glycosyltransferase; cell division activation (Woo et al., 1999 Plant Cell 11, 2303-2315)
132	profile 4	3,553824101	AC0605	DY615522	TC94313	60S ribosomal protein L19
133	profile 4	2,50132679	AC1288	DY615941	TC94406	12-oxophytodienoate reductase; jasmonic acid biosynthesis
134	profile 4	1,954462804	AC1428	DY616039	TC94398	26S proteasome subunit 4 AtRPT2a
135	profile 4	2,114996019	AC2486	DY616861	TC100179	ADP-ribosylation factor (small GTPase ARF); intracellular protein transport
136	profile 4	3,945629146	AC1115	DY615792	TC106434	tubulin beta; cytoskeleton
137	profile 4	2,210418845	AC1936	DY616445	TC107124	EF-hand Calcium binding protein; conserved plant protein
138	profile 4	2,486927332	AC0525	DY615456	TC101346	cell division control protein (cdk) 2
139	profile 4	2,164164704	AC0326	AJ388953	TC106500	60S ribosomal protein L18a
140	profile 4	2,813174408	AC0348	AJ388975	TC93950	60S ribosomal protein L27A
141	profile 4	1,679208556	AC1446	DY616057	TC100761	coatomer delta subunit (Delta COP); intracellular vesicle transport; Golgi vesicle
142	profile 4	5,007045486	AC0517	DY615448	TC95953	cyclin D3
143	profile 4	2,036475241	AC1702	DY616254	TC107031	cytosolic tRNA-Ala synthetase; translation; protein synthesis
144	profile 4	2,332617088	AC0222	AJ388856	TC106485	elongation factor 1-alpha, protein biosynthesis, regulation of translation
145	profile 4	2,066734351	AC2441	DY616832	TC100199	translation initiation factor eIF-4A; DEAD box RNA helicase
146	profile 4	1,726836323	AC3998	DY617814	TC95177	beta-1,3 glucanase (callose degradation)
147	profile 4	2,16928415	AC1627	DY616202	TC108345	alpha-glucosidase; starch metabolism; Hydrolysis of terminal, non-reducing 1,4-linked α-D-glucose residues with release of α-D-glucose
148	profile 4	2,80590201	AC4124	DY617917	TC94521	HSP90-like protein (SHEPHERD); Endoplasmin homolog; ER localized
149	profile 4	3,118665356	AC1383	DY616001	TC98647	heat shock protein
150	profile 4	2,149025015	AC0763	DY615675	TC100786	isoflavone reductase
151	profile 4	3,297026292	AC0721	DY615636	TC108572	MtN3-like; contains two transmembrane helices
152	profile 4	1,295605766	AC0234	AJ388866	TC94582	NADH-ubiquinone oxidoreductase 24 kDa subunit mitochondrial precursor; oxidative phosphorylation
153	profile 4	4,481302834	AC1381	DY616000	singleton	protein of unknown function
154	profile 4	1,617049555	AC4600	DY618282	TC102084	hypothetical protein, contains 3 transmembrane domains
155	profile 4	1,749486457	AC2250	DY616696	TC100615	nucleolin; contains 2 RRM (RNA recognition motif); role in pre-rRNA transcription and ribosome assembly; role in transcriptional elongation
156	profile 4	2,725879309	AC1549	DY616147	TC106342	poly-A Binding Protein; positive regulator of translation

157	profile 4	1,594328176	AC4015	DY617830	TC100933	potassium ion channel regulatory beta chain with aldo-keto oxidoreductase activity
158	profile 4	1,824127139	AC1170	DY615836	TC94135	26S proteasome subunit RPN5a
159	profile 4	2,374896623	AC1918	DY616430	TC107025	20S proteasome alpha 6 subunit
160	profile 4	3,780063682	AC4802	DY618433	TC94066	beta-1 3-glucanase
161	profile 4	2,820979081	AC1474	DY616082	TC94015	elongation factor EF-2; translation
162	profile 4	2,293581097	AC0284	AJ388914	TC101000	protein with unknown function
163	profile 4	1,499413187	AC1927	DY616438	TC107201	conserved plant protein; contains a N-terminal Sm-domain conserved in RNA binding proteins part of RNPs
164	profile 4	2,377236778	AC2268	DY616709	TC94934	protein with Tetratricopeptide repeat domain (TPR); unknown function
165	profile 4	2,106507473	AC2425	DY616823	TC101237	cop-coated vesicle membrane protein; secretory pathway
166	profile 4	1,998648033	AC2621	DY616963	TC106798	hypothetical protein; unknown function; DNA binding, homologous to Alba an archeal chromatin protein
167	profile 4	2,562845625	AC3191	DY617323	TC107034	conserved protein of unknown function; contains transmembrane domains
168	profile 4	1,784369115	AC3471	DY617469	TC109490	Armadillo/beta-catenin-like repeat containing protein of unknown function
169	profile 4	1,508432805	AC0350	AJ388977	TC111704	50S ribosomal protein L21 mitochondrial precursor
170	profile 4	1,686288263	AC1085	DY615769	TC100778	ribosomal protein L28
171	profile 4	2,491910597	AC2112	DY616583	TC106330	60S ribosomal protein L17
172	profile 4	3,217459641	AC2213	DY616668	TC101073	60S ribosomal protein L18
173	profile 4	2,718521243	AC2453	DY616836	TC94302	60S ribosomal protein L4
174	profile 4	2,244601802	AC0192	AJ388826	TC94496	60S ribosomal protein L12
175	profile 4	3,019581794	AC3032	DY617253	TC100447	60S ribosomal protein L13a-4
176	profile 4	2,483991082	AC0022	AJ388670	TC107120	ribosomal protein L14; hydroxyproline-rich glycoprotein
177	profile 4	2,548248334	AC0253	AJ388884	TC100421	60S ribosomal protein L15
178	profile 4	3,397997303	AC1844	DY616370	TC106334	ribosomal protein L2
179	profile 4	2,384281187	AC1052	DY615753	TC106429	ribosomal L22e protein
180	profile 4	2,133535467	AC3735	DY617609	TC94760	60S ribosomal protein L26
181	profile 4	1,850061651	AC1542	DY616141	TC106474	60S ribosomal protein L34
182	profile 4	2,381937673	AC0114	AJ388751	TC100995	60S ribosomal protein L35
183	profile 4	2,980373864	AC0054	AJ388695	TC100531	60S ribosomal protein L6
184	profile 4	2,299755803	AC1472	DY616080	TC100719	60S acidic ribosomal protein P1
185	profile 4	2,75001686	AC1798	DY616330	TC106748	40S ribosomal protein S10
186	profile 4	2,789824306	AC1926	DY616437	TC100470	40S ribosomal protein S11
187	profile 4	3,373993584	AC1529	DY616130	TC107406	40S ribosomal protein S12
188	profile 4	3,367140181	AC0196	AJ388830	TC106824	40S ribosomal protein S15-1
189	profile 4	2,618346275	AC0377	AJ389001	TC94278	40S ribosomal protein S2

190	profile 4	2,99553378	AC3563	DY617514	TC100931	40S ribosomal protein S20
191	profile 4	2,764133894	AC0290	AJ388920	TC106541	40S ribosomal protein S3
192	profile 4	2,329772607	AC1342	DY615976	TC93948	40S ribosomal S4 protein
193	profile 4	2,634616162	AC1746	DY616284	TC106523	40S ribosomal protein S6
194	profile 4	3,557221528	AC2119	DY616589	TC100149	40S ribosomal protein S7
195	profile 4	1,818761075	AC1329	DY615970	TC106644	40S ribosomal protein S9
196	profile 4	2,190300225	AC2691	DY617014	TC108094	signal peptide peptidase (SPP)
197	profile 4	1,617351458	AC2408	DY616809	TC101217	protein transport protein SEC13; secretory pathway; contains WD40 domain
198	profile 4	1,792995521	AC4328	DY618081	TC108419	U2 small nuclear ribonucleoprotein A' (U2 snRNP-A'); RNA splicing
199	profile 4	3,476310816	AC0338	AJ388965	TC106866	ubiquitin / ribosomal protein CEP52 (Ubiquitin fused to ribosomal protein L40)
200	profile 5	19,27522593	AC0655	DY615572	TC103668	protein with unknown function
201	profile 5	6,46254704	AC2722	DY617043	singleton	ubiquitin carboxyl-terminal hydrolase; deubiquitinating enzyme; MtN2
202	profile 5	6,018016746	AC4482	DY618196	TC100662	alcohol dehydrogenase
203	profile 5	5,148961243	AC2657	DY616990	TC95584	MtN9; MtMMPL1; Zinc-dependent metalloprotease; signal peptide
204	profile 5	18,36802283	AC1810	DY616342	TC102486	Thioredoxin S-type, (symbiotic-type); defense against oxidative stress (reducing hydrogen peroxide and certain radicals), reducing protein disulfide bonds and thereby regulation of protein (enzymes, transcription factors) functions; signal peptide
205	profile 5	6,869798705	AC4780	DY618415	TC102163	MtN1-like (defensin like)
206	profile 5	33,34792733	AC1608	DY616189	TC96169	nodulin 6 (MtN6); nodulin 61 (soybean); glutamate-ammonia ligase (glutamine syntase)
207	profile 5	10,41475901	AC4496	DY618205	singleton	protein with unknown function
208	profile 5	12,07040973	AC2126	DY616596	TC111120	protein with unknown function
209	profile 6	18,32982105	AC4018	DY617833	TC98964	caffeic acid O-methyltransferase (catalyzes the S-adenosylmethionine-dependent O-methylation of caffeic acid to form ferulic acid (antioxidant))
210	profile 6	19,04469603	AC0102	AJ388741	TC94567	NCR084
211	profile 6	26,11458658	AC1531	DY616132	singleton	NCR145
212	profile 6	30,5745779	AC3478	DY617472	TC109261	NCR213
213	profile 6	7,985041228	AC2302	DY616733	TC97740	NCR233
214	profile 6	5,787608499	AC2067	DY616542	singleton	NCR321
215	profile 6	7,338613812	AC3774	DY617638	TC98265	NCR336
216	profile 6	11,83497238	AC2577	DY616926	singleton	NCR356
217	profile 6	7,500150713	AC2834	DY617139	singleton	protein with unknown function

218	profile 6	5,029945076	AC3666	DY617554	singleton	HRD ubiquitin ligase complex, ER membrane component [Posttranslational modification, protein turnover, chaperones]
219	profile 6	4,928192698	AC2423	DY616821	TC100662	alcohol dehydrogenase
220	profile 6	99,8583964	AC0131	AJ388768	TC100150	carbonic anhydrase
221	profile 6	43,72509995	AC0643	DY615560	TC101756	Lipase/lipooxygenase, PLAT/LH2 (interaction with lipids or membrane bound proteins), Embryo-specific protein 3, (ATS3), signal peptide; (6 nodule specific genes on genomic locus - see clones AC1452; AC3976; AC643)
222	profile 6	10,43015702	AC1296	DY615949	TC100834	signal peptidase 25kDa subunit (SPC25); ER; protein secretion
223	profile 6	37,93077627	AC1371	DY615994	TC101347	enod16, signal peptide, proline-rich; related to enod20
224	profile 6	8,450738123	AC0237	AJ388868	TC100789	MtN1 (defensin like)
225	profile 6	23,533256	AC0366	AJ388991	TC102128	Small Nodulin Acidic RNA-binding Peptide SNARP3
226	profile 6	11,22080129	AC1721	DY616265	TC95535	nodule specific charged protein (100-120aa); glutamic acid (E) and lycine (K)-rich nodule specific protein from soybean; no homology
227	profile 6	2,982057887	AC2283	DY616721	singleton	secretory peroxidase; Class III of the plant heme-dependent peroxidase superfamily
228	profile 6	2,022875427	AC2974	DY617220	TC96114	conserved transmembrane protein of unknown function
229	profile 6	5,969600764	AC1051	DY615752	TC95076	signal peptide peptidase (SPP)
230	profile 6	4,343428603	AC1676	DY616235	TC96179	MtN21; integral membrane protein; permease; transporter
231	profile 6	105,9809008	AC1555	DY616152	TC109169	MtN25 protein; signal peptide; Glycine rich protein
232	profile 6	48,38471545	AC1589	DY616180	TC107188	MtN29 protein; Glycine rich protein
233	profile 6	8,21652518	AC4024	DY617837	singleton	GRP1C
234	profile 6	9,75189242	AC1002	DY615716	TC108339	NCR109 (MtN28)
235	profile 6	10,58990503	AC3400	DY617421	TC95075	signal peptide peptidase (SPP)
236	profile 6	5,505348818	AC0410	AJ389033	TC100580	proline-rich cell wall protein
237	profile 6	12,52007619	AC3895	DY617729	TC94812	UDP-sulfoquinovose synthase; Sulfolipid biosynthesis protein
238	profile 6	1,741055484	AC1915	DY616427	TC95407	Cytokinin oxidase, FAD-binding; cytokinin inactivation and degradation
239	profile 6	4,231940455	AC3135	DY617301	singleton	bZIP transcription factor TGA-type (TGACG MOTIF-BINDING)
240	profile 6	4,306548537	AC0385	AJ389008	TC102614	DNAJ protein (heat shock protein)
241	profile 6	2,388983036	AC1884	DY616400	TC94919	beta-tubulin (cytoskeleton)
242	profile 6	3,827311973	AC2063	DY616538	singleton	POT oligo peptide, proton dependent transporter
243	profile 6	1,725280236	AC4827	DY618455	TC94328	UDP-glucose pyrophosphorylase; starch and sugar metabolism
244	profile 6	2,591159716	AC2148	DY616613	singleton	NADH:flavin dependent 12-oxophytodienoate-10,11-reductase) (OPDA-reductase) jasmonic acid biosynthesis
245	profile 6	4,082165056	AC0240	AJ388871	TC93945	ATP synthase beta chain mitochondrial precursor

246	profile 6	10,42294217	AC3976	DY617795	singleton	Lipase/lipooxygenase, PLAT/LH2 (interaction with lipids or membrane bound proteins), Embryo-specific protein 3, (ATS3), signal peptide; (6 nodule specific genes on genomic locus - see clones AC1452; AC3976; AC643)
247	profile 6	7,906637969	AC0309	AJ388939	TC94515	nodulin ENOD40
248	profile 6	2,466154856	AC0060	AJ388701	TC100309	phosphopyruvate hydratase; enolase
249	profile 6	3,63777989	AC2561	DY616917	singleton	GATA-binding transcription facor
250	profile 6	5,540216568	AC1858	DY616384	TC94194	granule-bound glycogen (Starch) synthase
251	profile 6	2,41306495	AC0097	AJ388736	TC100430	cytosolic malate dehydrogenase
252	profile 6	127,3429529	AC0024	AJ388672	TC107014	MtN22, signal peptide; same genomic locus as CaM-like proteins (clone AC0344) and nodulin25 (clone AC0154)
253	profile 6	11,07656672	AC0210	AJ388844	TC100608	MtN5 protein; lipid transfer protein
254	profile 6	2,890605877	AC0259	AJ388890	singleton	MRP-like ABC transporter (Glutathione S-conjugate-transporting ATPase)
255	profile 6	17,29901527	AC1912	DY616424	TC107654	MtN24; conserved plant protein with transmembrane helices
256	profile 6	8,880172045	AC0360	AJ388985	TC109579	protein of unknown function with DUF640 domain
257	profile 6	5,881307981	AC3954	DY617778	TC109112	CBS, Phox and Bem1p domains-containing protein; signal transduction protein of unknown function
258	profile 6	3,669823098	AC4398	DY618136	TC110990	conserved protein of unknown function
259	profile 6	18,23460853	AC4151	DY617937	TC96630	remorin; plant-specific membrane associated and lipid rafts associated protein of unknown function; phospho-protein; signalling
260	profile 6	5,143584961	AC2150	DY616615	TC95444	pathogenesis related protein of unknown function; PR-1 like; signal peptide and extracellular SCP domain conserved in plant and human immune system
261	profile 6	3,164103176	AC0257	AJ388888	TC100410	sucrose synthase
262	profile 6	3,499784262	AC1424	DY616036	singleton	peptide/amino acid transporter
263	profile 6	1,87590363	AC1940	DY616448	TC95445	actin depolymerizing factor (ADF)/cofilin; cytoskeleton
264	profile 6	6,662455917	AC4175	DY617957	singleton	caffeic acid O-methyltransferase (catalyzes the S-adenosylmethionine-dependent O-methylation of caffeic acid to form ferulic acid (antioxidant))
265	profile 6	7,428367205	AC1116	DY615793	singleton	defensin-like peptide
266	profile 6	6,686255308	AC3835	DY617690	singleton	ER lumen protein retaining receptor
267	profile 6	2,340261495	AC1722	DY616266	singleton	fructokinase;glycolysis
268	profile 6	3,728430031	AC0029	AJ388674	TC106913	glutamine synthetase
269	profile 6	9,339259817	AC0064	AJ388705	singleton	no ORF in EST; homologous to the 3' end of the GRP5B
270	profile 6	3,425509916	AC4107	DY617902	TC102728	transcription factor; C2H2-type zinc finger protein
271	profile 6	5,190849615	AC2154	DY616619	TC109630	secreted protein of unknown function related to MtN26
272	profile 6	8,105085346	AC1277	DY615931	TC97495	NCR237
273	profile 6	4,798950838	AC1455	DY616066	singleton	NCR110
274	profile 6	6,172296079	AC2863	DY617157	singleton	NCR128

275	profile 6	4,946650905	AC2612	DY616956	TC97025	NCR201
276	profile 6	11,45883558	AC0242	AJ388873	TC102095	NCR206
277	profile 6	9,189584705	AC2884	DY617169	singleton	NCR351
278	profile 6	5,621985001	AC2494	DY616867	singleton	NCR327
279	profile 6	16,77020542	AC4473	DY618190	TC110997	NCR339
280	profile 6	4,741606399	AC4075	DY617875	singleton	NCR342
281	profile 6	2,085419686	AC0353	AJ388979	TC106697	nucleoside diphosphate kinase I (NDK I) (NDP kinase I) (NDPK I)
282	profile 6	2,197736842	AC1099	DY615778	TC95756	conserved protein of unknown function
283	profile 6	2,491043547	AC1108	DY615786	TC95643	protein of unknown function , conserved in plants
284	profile 6	5,002835419	AC2376	DY616786	TC95075	signal peptide peptidase (SPP)
285	profile 6	3,866558726	AC4515	DY618219	singleton	NCR340
286	profile 6	3,824966036	AC1278	DY615932	TC95087	alpha-1,4 glucan phosphorylase L isozyme, chloroplast/amyloplast precursor
287	profile 6	5,301270053	AC3122	DY617294	TC95550	aspartyl (acid) protease; secreted
288	profile 6	6,724880925	AC0505	DY615436	TC100731	enod20; proline-rich; related to enod16
289	profile 6	8,114013977	AC4126	DY617919	singleton	ER lumen protein retaining receptor
290	profile 6	3,263754525	AC2590	DY616937	TC94500	ferritin 2 chloroplast precursor
291	profile 6	13,07507354	AC4626	DY618300	TC108069	NCR119
292	profile 6	6,133029594	AC2461	DY616841	TC100321	NCR123
293	profile 6	24,25362815	AC1502	DY616107	TC95912	NCR220 (MtN27)
294	profile 6	4,808400033	AC4051	DY617856	TC96632	NCR244
295	profile 6	5,205014529	AC1544	DY616143	singleton	NCR319
296	profile 6	10,21414864	AC2212	DY616667	singleton	NCR326
297	profile 6	10,40863076	AC3467	DY617465	TC107275	protein of unknown function
298	profile 6	2,72778908	AC2666	DY616997	singleton	NCR347
299	profile 6	27,02906357	AC1954	DY616460	singleton	allantoin (Ureide) permease (L. japonicus is an amide transporter; soybean is an ureide transporter)
300	profile 6	5,273996307	AC4100	DY617895	singleton	TIR domain protein; resistance
301	profile 6	2,379315661	AC1232	DY615889	TC96933	UV-damaged DNA binding factor; re-mRNA cleavage and polyadenylation specificity factor [RNA processing and modification]
302	profile 6	5,645284139	AC2694	DY617017	singleton	transcription factor; nodule inception protein (nin)
303	profile 6	3,356145003	AC2155	DY616620	TC96274	secreted aspartyl protease (pepsin A)
304	profile 6	2,722717613	AC0277	AJ388908	TC103041	allantoin (Ureide) permease (L. japonicus is an amide transporter; soybean is an ureide transporter)
305	profile 6	2,401611144	AC3480	DY617474	TC96501	bHLH transcription factor
306	profile 6	2,009581345	AC3497	DY617484	TC96184	protein of unknown function
307	profile 6	2,723878652	AC3822	DY617677	TC107034	conserved protein of unknown function

308	profile 6	2,184394992	AC4299	DY618058	TC109990	protein phosphatase 2C; serine/threonine phosphatase; signal transduction
309	profile 6	16,65924545	AC1377	DY615997	TC96231	signal peptidase 22kDa subunit (SPC22); ER; protein secretion
310	profile 6	11,70859234	AC2699	DY617021	TC95981	transcription factor CCAAT-binding nuclear factor Y subunit (HAP2); Combier et al., 2006
311	profile 6	3,545786817	AC4480	DY618194	TC95480	conserved protein, unknown function
312	profile 6	2,208805129	AC1701	DY616253	TC96544	MtN21-like; integral membrane protein; permease; transporter; conserved in plants
313	profile 6	3,17234287	AC0105	AJ388744	TC100510	40S ribosomal protein S8
314	profile 6	1,966915064	AC3093	DY617276	TC94731	glucose-6-phosphate/phosphate translocator
315	profile 6	1,995369601	AC4577	DY618266	TC103210	kinesin
316	profile 6	1,977830874	AC1205	DY615865	singleton	Mitochondrial carrier protein; transport of metabolites in and out the mitochondrion
317	profile 6	1,783927978	AC3807	DY617665	TC108396	protein of unknown function with ribosomal protein S6 domain
318	profile 7	63,69778309	AC1444	DY616055	TC95391	leghemoglobin 29
319	profile 7	24,71569945	AC1816	DY616347	TC109065	NCR028
320	profile 7	42,37187086	AC1083	DY615767	TC110997	NCR306
321	profile 7	20,62539189	AC0279	AJ388910	TC94903	NCR095
322	profile 7	33,4194598	AC3921	DY617752	TC100432	enod8; suger esterase
323	profile 7	27,22770706	AC0081	AJ388721	TC100432	enod8; suger esterase
324	profile 7	8,579333275	AC3505	DY617487	TC95255	nodule specific lectin; homologous to PsNlec1
325	profile 7	106,7004289	AC0304	AJ388934	TC107718	GRP3A, nodule-specific glycine-rich protein 3
326	profile 7	9,027415238	AC0074	AJ388715	TC106138	MtN20 like
327	profile 7	43,88648739	AC0123	AJ388760	TC94240	NCR053
328	profile 7	27,51067031	AC2581	DY616929	TC97251	NCR140
329	profile 7	21,90117457	AC0164	AJ388801	TC101084	NCR165
330	profile 7	16,68211431	AC2098	DY616570	TC101764	NCR172
331	profile 7	14,80042842	AC1086	DY615770	TC94693	NCR316
332	profile 7	55,09728143	AC2724	DY617045	singleton	NCR331
333	profile 7	11,2675428	AC4165	DY617948	TC107718	GRP3
334	profile 7	24,77324247	AC0418	AJ389041	TC100851	nodulin 26 aquaporin; symbiosome membrane protein
335	profile 7	2,578684595	AC1113	DY615790	TC101191	prolyl 4-hydroxylase alpha subunit; protein maturation in ER
336	profile 7	10,45720407	AC1540	DY616139	TC101252	sulfate transporter (orthologue of L. japonicus Sst1; Krussell et al., 2005 Plant Cell 17:1625-1636)
337	profile 7	136,091272	AC0215	AJ388849	TC106591	leghemoglobin
338	profile 7	25,84179179	AC1195	DY615857	singleton	NCR042
339	profile 7	35,41102561	AC2999	DY617232	TC94003	NCR164

340	profile 7	28,60049739	AC2195	DY616651	singleton	NCR325
341	profile 7	17,2963192	AC1291	DY615944	singleton	NCR343
342	profile 7	8,46142504	AC0347	AJ388974	singleton	NCR135
343	profile 7	5,55617843	AC3990	DY617807	TC101021	26S proteasome regulatory particle chain RPT6; ATPase; binds ubiquitin protein ligases
344	profile 7	3,59962499	AC3212	DY617337	singleton	transmembrane amino acid transporter protein; amino acid permease
345	profile 7	11,13905288	AC2344	DY616761	TC102092	carbonic anhydrase, nodule specific
346	profile 7	11,00122375	AC1452	DY616063	singleton	lipase/lipooxygenase, PLAT/LH2 (interaction with lipids or membrane bound proteins), Embryo-specific protein 3, (ATS3)
347	profile 7	22,52086872	AC0376	AJ389000	singleton	GRP
348	profile 7	109,1978476	AC1220	DY615879	TC111259	GRP1L
349	profile 7	15,66489484	AC4651	DY618319	TC107043	Universal stress protein (Usp); induced upon stress; increase survival upon stress
350	profile 7	14,59222326	AC2037	DY616518	TC101259	GATA-binding transcription factor (Zn finger)
351	profile 7	4,830428198	AC0585	DY615505	TC107287	hexose transporter protein
352	profile 7	97,62000373	AC3629	DY617527	TC94045	histone H2B
353	profile 7	14,99776419	AC4419	DY618152	TC98260	lipase/esterase; similar to enod8
354	profile 7	129,0347818	AC0020	AJ388668	TC106578	leghemoglobin 2
355	profile 7	135,3227147	AC0108	AJ388746	TC106593	leghemoglobin
356	profile 7	51,08288559	AC0130	AJ388767	TC106592	leghemoglobin 1
357	profile 7	95,37935568	AC0146	AJ388783	TC106579	leghemoglobin
358	profile 7	115,8242723	AC0224	AJ388857	TC100586	leghemoglobin 29
359	profile 7	64,71199693	AC1071	DY615763	TC106587	leghemoglobin
360	profile 7	26,3914911	AC3245	DY617357	singleton	amino acid transproter; Lysine and histidine specific transporter; amino acid permease
361	profile 7	24,41770326	AC0288	AJ388918	TC94518	enod2/nodulin75/N8 (other nodule specific homologue:TC106596)
362	profile 7	8,903604622	AC1890	DY616405	TC103322	NCR081
363	profile 7	15,56958725	AC1379	DY615998	TC106595	NCR086
364	profile 7	6,543374842	AC0212	AJ388846	TC100607	NCR094
365	profile 7	7,743300495	AC3386	DY617415	TC101238	NCR103
366	profile 7	21,08831396	AC0263	AJ388894	TC102899	NCR105
367	profile 7	6,560819034	AC3021	DY617244	singleton	NCR278
368	profile 7	17,19422103	AC2542	DY616904	singleton	NCR329
369	profile 7	24,15418448	AC1635	DY616207	TC104158	Mitogen Activated Kinase (MAPK)
370	profile 7	222,8350203	AC0154	AJ388791	TC106696	nodulin 25
371	profile 7	12,39378825	AC1658	DY616222	TC108598	phosphoethanolamine N-methyltransferase
372	profile 7	5,748646447	AC0091	AJ388730	TC95187	proline rich protein

373	profile 7	9,146754987	AC1132	DY615806	TC100814	protein of unknown function
374	profile 7	7,490510935	AC1672	DY616232	TC107129	Universal stress protein (Usp); induced upon stress; increase survival upon stress
375	profile 7	8,502222071	AC3325	DY617391	TC109930	IQ calmodulin-binding protein
376	profile 7	20,38870687	AC4391	DY618132	singleton	complex I intermediate-associated protein 30; chaperone protein involved in the assembly of the mitochondrial NADH:ubiquinone oxidoreductase complex I
377	profile 7	16,30544737	AC4415	DY618148	singleton	conserved protein of unknown function; contains transmembrane domains
378	profile 7	74,62462479	AC0113	AJ389051	TC102213	protein of unknown function, contains ankyrin repeats (protein-protein interactions)
379	profile 7	9,57289634	AC0580	DY615500	TC98374	wound induced protein of unknown function
380	profile 7	6,630154893	AC0233	AJ388865	TC93925	triosephosphate isomerase
381	profile 7	3,195495136	AC1864	DY616387	TC100894	NCR032
382	profile 7	9,0306047	AC3917	DY617748	TC107608	NCR035
383	profile 7	13,51518013	AC0189	AJ388823	TC97593	NCR102
384	profile 7	27,85873079	AC1412	DY616025	singleton	NCR317
385	profile 7	16,57395632	AC1953	DY616459	singleton	NCR344
386	profile 7	5,019345206	AC1390	DY616006	singleton	Prolyl 4-hydroxylase, alpha subunit
387	profile 7	6,342441963	AC2638	DY616978	singleton	RING/C3HC4/PHD zinc finger protein; ubiquitin protein ligase
388	profile 7	7,41178202	AC3933	DY617763	singleton	N-21 (soybean) homologue; conserved protein in plants; integral membrane protein
389	profile 7	3,209581849	AC0258	AJ388889	TC110049	pyridoxal kinase; vitamin B6 synthesis
390	profile 7	3,138861759	AC3490	DY617480	TC106719	SGT1; regulation of cell division; SCF-mediated ubiquitination of proteins; pathogen resistance; functions as a cochaperone
391	profile 8	37,64911232	AC0287	AJ388917	TC93931	basic blue cupper-binding protein, electron transport
392	profile 8	7,106802376	AC2105	DY616577	TC95710	NCR018
393	profile 8	14,62071212	AC0262	AJ388893	TC104756	NCR169
394	profile 8	33,80619559	AC3355	DY617401	singleton	NCR333
395	profile 8	4,467332624	AC1297	DY615950	TC108792	protein of unknown function; similar to pathogenesis-related protein
396	profile 8	4,403595099	AC3455	DY617456	TC107024	ARG10; protein of unknown function; responive to auxin and aluminium; possible asparagine synthethase
397	profile 8	6,012418913	AC1807	DY616339	TC106729	glutamine synthetase
398	profile 8	87,84422988	AC0068	AJ388709	TC106577	NCR001
399	profile 8	39,00829529	AC3183	DY617319	TC101991	NCR003
400	profile 8	35,9568433	AC0312	AJ388941	TC94540	NCR019
401	profile 8	34,03871611	AC1715	DY616261	TC102115	NCR072
402	profile 8	12,19466106	AC1960	DY616464	TC107422	NCR160
403	profile 8	15,51400378	AC1140	DY615813	singleton	NCR287

404	profile 8	15,60170168	AC0390	AJ389013	singleton	NCR312
405	profile 8	16,62496821	AC0193	AJ388827	singleton	endoplasmatic reticulum retrieval protein RER1, protein secretion
406	profile 8	6,01513388	AC0356	AJ388981	singleton	protein of unknown function
407	profile 8	13,84246425	AC1670	DY616230	singleton	protein of unknown function
408	profile 8	2,881206358	AC0205	AJ388839	TC94720	conserved protein of unknown function
409	profile 8	2,748456247	AC2187	DY616646	TC107737	wound induced protein (91aa) of unknown function; conserved in plants
410	profile 8	5,265363081	AC2625	DY616967	TC94674	alternative respiratory pathway oxidase
411	profile 8	3,827665761	AC0129	AJ388766	TC111984	low affinity sulfate transporter 3
412	profile 8	8,249763865	AC3923	DY617754	TC108713	NCR002
413	profile 8	28,23136343	AC1489	DY616094	TC94921	NCR006
414	profile 8	22,81907609	AC0169	AJ388805	TC108507	NCR009
415	profile 8	43,15085048	AC2317	DY616738	TC108455	NCR040
416	profile 8	9,585135008	AC2411	DY616812	singleton	NCR079
417	profile 8	17,33485481	AC0093	AJ388732	TC107446	NCR099
418	profile 8	78,30321637	AC0286	AJ388916	singleton	NCR108
419	profile 8	41,2858991	AC0133	AJ388770	TC100865	NCR113
420	profile 8	9,909119334	AC1228	DY615887	TC95930	NCR219
421	profile 8	14,92545848	AC2622	DY616964	singleton	NCR330
422	profile 8	4,381587106	AC4472	DY618189	singleton	NCR338
423	profile 8	5,883695805	AC1262	DY615916	TC108269	GcpE; 1-hydroxy-2-methyl-2-(E)-butenyl 4-diphosphate synthase for plastid isoprenoid synthesis via the MEP pathway
424	profile 8	8,533649547	AC2498	DY616870	TC108234	Class III peroxidase (homologous to rip1)
425	profile 8	4,88761657	AC1358	DY615983	TC111751	polypyrimidine tract-binding protein; mRNA processing; mRNA splicing
426	profile 8	57,87591911	AC3640	DY617534	TC108455	NCR040
427	profile 8	3,65580492	AC4692	DY618351	TC95234	auxin responsive SAUR protein
428	profile 8	28,09038067	AC0344	AJ388971	TC101807	calmodulin-like protein
429	profile 8	45,69284886	AC0049	AJ388690	TC107339	enod18; cytosolic, potential ATP binding protein
430	profile 8	4,168371146	AC1185	DY615850	TC94780	NADH-dependent glutamate synthase
431	profile 8	3,388117556	AC1805	DY616337	singleton	protein of unknown function
432	profile 8	4,27954299	AC3838	DY617693	TC101134	mannosyl-oligosaccharide 1 2-alpha-mannosidase
433	profile 8	10,59781415	AC4342	DY618094	TC94421	NCR007
434	profile 8	19,5992627	AC0230	AJ388862	TC108178	NCR036
435	profile 8	6,759928825	AC1573	DY616167	TC95706	NCR097
436	profile 8	24,01424731	AC0239	AJ388870	singleton	NCR125
437	profile 8	21,29897256	AC1253	DY615907	TC101763	NCR159
438	profile 8	6,616136465	AC0571	DY615493	TC111268	NCR230

439	profile 8	75,23900331	AC1413	DY616026	TC106362	NCR269
440	profile 8	15,92486911	AC2102	DY616574	singleton	NCR324
441	profile 8	3,414304076	AC0664	DY615580	singleton	protein of unknown function
442	profile 8	5,138481472	AC2432	DY616829	singleton	protein of unknown function
443	profile 8	3,39849701	AC2088	DY616562	TC95035	AP2/EREBP transcription factor
444	profile 8	14,37076067	AC3165	DY617311	TC108316	Small Nodulin Acidic RNA-binding Peptide SNARP1
445	profile 8	5,709491988	AC2515	DY616884	TC108792	pathogenesis related protein
446	profile 8	47,37208753	AC2925	DY617196	TC102991	calcium-binding EF-hand; adenine nucleotide translocator 1
447	profile 8	3,521679737	AC1834	DY616361	TC100830	CCCH-type zinc finger transcription factor
448	profile 8	5,252752975	AC4379	DY618123	TC97287	glutamine amidotransferase class I
449	profile 8	3,766170891	AC0089	AJ388728	TC107091	protein of the AS2 family (AS2-like or ASL) with LOB-domain class II; plant specific domain; possible transcription factor
450	profile 8	5,760911388	AC1192	DY615855	TC104742	homologous to uncharacterized protein containing DHHC-type Zn finger
451	profile 8	3,284922627	AC2470	DY616848	TC107091	protein of the AS2 family (AS2-like or ASL) with LOB-domain class II; plant specific domain; possible transcription factor
452	profile 8	5,367988475	AC2778	DY617090	TC96682	protein of unknown function
453	profile 8	3,266798647	AC0278	AJ388909	TC101043	pyrophosphate-dependent phosphofructo-1-kinase (glycolysis)
454	profile 8	17,79612441	AC1528	DY616129	TC94722	senescence associated protein with Rhodanese Homology Domain (RHOD)
455	profile 8	4,300360863	AC0250	AJ388881	TC98666	similar to wound induced protein of unknown function
456	profile 8	7,749595304	AC3156	DY617308	singleton	carbonic anhydrase
457	profile 8	3,798699553	AC2177	DY616641	TC100753	NCR
458	profile 8	9,023422578	AC3206	DY617332	TC95501	NCR011
459	profile 8	5,593949352	AC3111	DY617286	TC100893	NCR184
460	profile 8	58,97698078	AC3124	DY617296	TC95184	NCR238
461	profile 8	5,553089688	AC2331	DY616749	singleton	NCR346
462	profile 8	5,691612401	AC2864	DY617158	singleton	NCR350
463	profile 8	2,013371834	AC4706	DY618364	TC95558	protein of unknown function
464	profile 8	5,18853275	AC0361	AJ388986	TC101066	myosin heavy chain; Smc-domain, Chromosome segregation ATPases; Cell division and chromosome partitioning
465	profile 8	2,447511753	AC1654	DY616219	TC106560	S-adenosylmethionine synthase
466	profile 8	4,647513428	AC2713	DY617034	TC94896	chitinase class III
467	profile 8	7,793249745	AC2394	DY616798	TC95833	NCR208
468	profile 8	2,570951774	AC2051	DY616531	TC111920	polygalacturonase PG1
469	profile 8	4,919593974	AC0207	AJ388841	TC95900	serine carboxypeptidase
470	profile 8	2,111238577	AC2180	DY616643	TC95517	secreted aspartic proteinase (senescence associated)
471	profile 8	2,584561907	AC4691	DY618350	TC96290	chitinase

472	profile 8	2,820139859	AC4353	DY618103	TC96056	cysteine proteinase
473	profile 8	6,133491659	AC0393	AJ389016	TC98625	cysteine proteinase
474	profile 8	6,344526295	AC1380	DY615999	TC100440	cysteine proteinase
475	profile 8	6,187199866	AC1811	DY616343	TC110797	class III chitinase
476	profile 8	1,962892604	AC2835	DY617140	singleton	POT family protein (proton-dependent oligopeptide transport); Dipeptide/tripeptide permease
477	profile 8	3,072073733	AC1080	DY615766	TC101701	acyl-CoA oxidase; fatty acid metabolism; peroxisome
478	profile 8	2,1658412	AC1105	DY615783	TC106745	ARG10; protein of unknown function; responive to auxin and aluminium; possible asparagine synthethase (homologous to AC3455=TC107024)
479	profile 8	28,1170654	AC0037	AJ388679	TC100391	asparagine synthetase
480	profile 8	21,2828082	AC1367	DY615990	TC100391	asparagine synthetase
481	profile 8	19,28409453	AC2820	DY617127	TC100393	asparagine synthetase
482	profile 8	6,501217985	AC3384	DY617413	TC94631	aspartate aminotransferase
483	profile 8	10,70328673	AC3246	DY617358	singleton	NCR023
484	profile 8	2,545483239	AC2530	DY616895	TC100502	cytochrome P450 [Secondary metabolites biosynthesis, transport, and catabolism]
485	profile 8	8,574490175	AC1272	DY615926	singleton	NCR078
486	profile 8	4,925427483	AC1030	DY615736	singleton	NCR314
487	profile 8	3,21056225	AC0087	AJ388726	singleton	NCR
488	profile 8	3,971367264	AC2079	DY616553	singleton	reverse transcriptase retrotransposon (remnant); homologous to nodule specific retrotransposon TC102933
489	profile 8	4,114088694	AC2658	DY616991	singleton	RNA-directed DNA polymerase (Reverse transcriptase); homologous to nodule specific retrotransposon TC102933
490	profile 8	2,019956693	AC2967	DY617216	TC103144	oxidoreductase; secondary metabolism; senescence-associated nodulin Glycine max
491	profile 8	3,796237808	AC2943	DY617207	TC94214	defensin (76aa); Gamma-thionin protease inhibitor; Knottin family; signal peptide
492	profile 8	2,380450481	AC0313	AJ388942	TC95881	apyrase-like protein
493	profile 8	2,689942373	AC0737	DY615650	TC106991	heme oxygenase 1
494	profile 8	2,334750538	AC1122	DY615798	TC107005	protein of unknown function
495	profile 8	2,563675083	AC1363	DY615987	TC99503	conserved protein of unknown function; cupin family; suger isomerase
496	profile 8	5,308759802	AC1861	DY616386	singleton	calmodulin-binding protein with IQ calmodulin-binding motif
497	profile 8	2,920603539	AC2019	DY616509	TC98799	conserved protein of unknown function; contains Smc domain for Chromosome segregation ATPases
498	profile 8	1,684049671	AC2065	DY616540	TC101943	nucleotide kinase
499	profile 8	2,439532927	AC2615	DY616957	TC107035	soluble diacylglycerol acyltransferase
500	profile 8	5,258898214	AC4317	DY618071	TC100441	protein of unknown function

501	profile 8	6,892068084	AC0332	AJ388959	TC100432	enod8; suger esterase
502	profile 8	2,410560637	AC2650	DY616985	TC95965	transcription factor; bZIP transcriptional activator
503	profile 8	1,870589602	AC1033	DY615738	TC101115	membrane protein of unknown function
504	profile 8	2,001388243	AC2204	DY616659	TC108059	trehalose-6-phosphate phosphatase
505	profile 8	3,662683215	AC4150	DY617936	TC106537	chalcone synthase
506	profile 8	7,680585186	AC3695	DY617578	TC102310	NCR052
507	profile 8	3,759492213	AC0245	AJ388876	TC106462	NCR120
508	profile 8	5,858612917	AC2591	DY616938	singleton	NCR250
509	profile 8	6,420140932	AC2535	DY616898	singleton	NCR328
510	profile 8	3,315403918	AC1050	DY615751	TC108488	protein of unknown function
511	profile 8	1,866311518	AC1056	DY615755	singleton	protein of unknown function
512	profile 8	3,378925041	AC1180	DY615846	singleton	peptide/nitrate transporter
513	profile 8	1,328227547	AC3020	DY617243	TC109073	protein of unknown function
514	profile 8	2,045694304	AC4497	DY618206	TC108560	2OG-Fe(II) oxygenase superfamily
515	profile 8	4,011599097	AC3128	DY617298	TC96367	cellulase (glycosyl hydrolase family 5)
516	profile 8	3,979227979	AC1469	DY616078	singleton	NCR318
517	profile 8	3,856885122	AC4485	DY618198	singleton	phosphate transporter
518	profile 8	1,959747432	AC1301	DY615954	TC104041	protein of unknown function
519	profile 8	2,807641995	AC4439	DY618164	TC103017	receptor like kinase (RLK); signal transduction
520	profile 8	8,100965343	AC1481	DY616088	TC109232	NCR039
