# Supplementary material for: Differentiation of Symbiotic Cells and Endosymbionts in Medicago truncatula Nodulation Are Coupled to Two Transcriptome-Switches
Source: PLoS One. 2010 Mar 4;5(3):e9519. doi: 10.1371/journal.pone.0009519 (PMC2832008; doi:10.1371/journal.pone.0009519)
Supplement: Table S4 — List of the clones present on the custom microarrays. Clones were isolated from a M. truncatula R108 nodule cDNA library. Clone identification numbers for internal use are given together with GenBank accession numbers at http://www.ncbi.nlm.nih.gov/entrez/ and MtGI accession numbers at http://compbio.dfci.harvard.edu/tgi/. (0.10 MB PDF) [file pone.0009519.s008.pdf]

clone N°	GenBank Accession	MtGI TC N°
0019	AJ388667	TC98930
0020	AJ388668	TC106578
0022	AJ388670	TC107120
0023	AJ388671	TC93964
0024	AJ388672	TC107014
0028	AJ388673	TC93973
0029	AJ388674	TC106913
0031	AJ388675	TC100176
0032	AJ388676	TC94754
0033	AJ388677	TC108508
0035	AJ388678	singleton
0037	AJ388679	TC100391
0038	AJ388680	TC100446
0039	AJ388681	singleton
0040	AJ388682	TC94277
0041	AJ388683	TC106485
0042	AJ388684	TC94718
0043	AJ389056	TC106354
0044	AJ388685	TC107101
0045	AJ388686	TC96826
0046	AJ388687	TC94988
0047	AJ388688	TC100709
0048	AJ388689	singleton
0049	AJ388690	TC107339
0050	AJ388691	TC106673
0051	AJ388692	TC93939
0052	AJ388693	TC97228
0053	AJ388694	TC106766
0054	AJ388695	TC100531
0055	AJ388696	TC107307
0056	AJ388697	singleton
0057	AJ388698	TC106925
0058	AJ388699	TC106598
0059	AJ388700	singleton
0060	AJ388701	TC100309
0061	AJ388702	TC94746
0062	AJ388703	TC108704
0063	AJ388704	singleton
0064	AJ388705	singleton
0065	AJ388706	TC106580
0066	AJ388707	TC96363
0067	AJ388708	TC112477
0068	AJ388709	TC106577
0069	AJ388710	TC106329
0070	AJ388711	TC94822
0071	AJ388712	TC107642
0072	AJ388713	TC106473
0073	AJ388714	TC94210
0074	AJ388715	TC106138
0076	AJ388716	TC96103
0077	AJ388717	TC107346
0078	AJ388718	TC109584

0079	AJ388719	singleton
0080	AJ388720	TC100549
0081	AJ388721	TC100432
0082	AJ388722	TC106773
0083	AJ388723	TC104825
0084	AJ388724	TC103061
0085	AJ388725	TC93930
0087	AJ388726	singleton
0088	AJ388727	singleton
0089	AJ388728	TC107091
0090	AJ388729	TC94386
0091	AJ388730	TC95187
0092	AJ388731	TC95257
0093	AJ388732	TC107446
0094	AJ388733	singleton
0095	AJ388734	TC97307
0096	AJ388735	TC106365
0097	AJ388736	TC100430
0098	AJ388737	TC94402
0099	AJ388738	TC100443
0100	AJ388739	TC94397
0101	AJ388740	TC94691
0102	AJ388741	TC94567
0103	BQ079321	singleton
0104	AJ388743	singleton
0105	AJ388744	TC100510
0106	AJ388745	TC107863
0107	AJ389050	TC106578
0108	AJ388746	TC106593
0109	AJ388747	TC96384
0110	AJ388748	TC107342
0111	AJ388749	TC94384
0112	AJ388750	TC106733
0113	AJ389051	TC102213
0114	AJ388751	TC100995
0115	AJ388752	TC100225
0116	AJ388753	TC102857
0117	AJ388754	singleton
0118	AJ388755	TC103712
0119	AJ388756	singleton
0120	AJ388757	TC95457
0121	AJ388758	TC96029
0122	AJ388759	TC108009
0123	AJ388760	TC94240
0124	AJ388761	TC109385
0125	AJ388762	singleton
0126	AJ388763	TC107020
0128	AJ388765	TC95325
0129	AJ388766	TC111984
0130	AJ388767	TC106592
0131	AJ388768	TC100150
0133	AJ388770	TC100865
0134	AJ388771	TC107872
0135	AJ388772	TC106563
0136	AJ388775	TC101916
0137	AJ388773	singleton

0138	AJ389052	TC94835
0139	AJ388776	TC106721
0141	AJ388778	TC100755
0142	AJ388779	TC98063
0143	AJ388780	TC100308
0144	AJ388781	singleton
0145	AJ388782	TC94384
0146	AJ388783	TC106579
0147	AJ388784	TC106953
0148	AJ388785	TC111389
0149	AJ388786	TC101064
0150	AJ388787	singleton
0151	AJ388788	singleton
0152	AJ388789	TC95776
0153	AJ388790	TC94624
0154	AJ388791	TC104921
0155	AJ388792	singleton
0156	AJ388793	TC106425
0157	AJ388794	TC96235
0159	AJ388796	singleton
0160	AJ388797	TC107190
0161	AJ388798	TC108776
0162	AJ388799	TC99781
0163	AJ388800	singleton
0164	AJ388801	TC101084
0165	AJ388802	singleton
0166	AJ388803	singleton
0169	AJ388805	TC108507
0170	AJ388806	singleton
0171	AJ388807	TC100467
0172	AJ388808	TC110270
0173	AJ388809	TC96168
0174	AJ388810	TC112393
0175	AJ388811	TC108034
0176	AJ388812	singleton
0177	AJ388813	TC100759
0178	AJ388814	TC107470
0179	AJ388815	TC106310
0180	AJ388816	TC97161
0181	AJ388817	singleton
0182	AJ388818	TC98782
0183	AJ388819	TC93977
0184	AJ389053	singleton
0186	AJ388820	TC108449
0187	AJ388821	TC98767
0188	AJ388822	TC101307
0189	AJ388823	TC97593
0190	AJ388824	singleton
0191	AJ388825	TC103840
0192	AJ388826	TC94496
0193	AJ388827	singleton
0195	AJ388829	TC106380
0196	AJ388830	TC106824
0197	AJ388831	singleton
0198	AJ388832	TC109316
0200	AJ388834	singleton

0201	AJ388835	TC94538
0202	AJ388836	TC98857
0203	AJ388837	TC106620
0204	AJ388838	TC97735
0205	AJ388839	TC94720
0206	AJ388840	TC102963
0207	AJ388841	TC95900
0209	AJ388843	TC107121
0210	AJ388844	TC100608
0211	AJ388845	singleton
0212	AJ388846	TC100607
0213	AJ388847	TC107039
0214	AJ388848	TC110814
0215	AJ388849	TC106591
0216	AJ388850	TC107148
0217	AJ388851	TC110093
0218	AJ388852	singleton
0220	AJ388854	singleton
0221	AJ388855	TC100955
0222	AJ388856	TC106485
0224	AJ388857	TC100586
0226	AJ388859	singleton
0229	AJ388861	TC106594
0230	AJ388862	TC108178
0231	AJ388863	singleton
0233	AJ388865	TC93925
0234	AJ388866	TC94582
0236	AJ388867	TC100533
0237	AJ388868	TC100789
0238	AJ388869	TC107436
0239	AJ388870	singleton
0240	AJ388871	TC93945
0241	AJ388872	TC94296
0242	AJ388873	TC102095
0243	AJ388874	TC104099
0244	AJ388875	singleton
0245	AJ388876	TC106462
0246	AJ388877	TC95164
0247	AJ388878	singleton
0249	AJ388880	singleton
0250	AJ388881	TC98666
0251	AJ388882	singleton
0252	AJ388883	TC107790
0253	AJ388884	TC100421
0254	AJ388885	singleton
0255	AJ388886	TC111940
0256	AJ388887	TC107267
0257	AJ388888	TC100410
0258	AJ388889	TC110049
0259	AJ388890	singleton
0260	AJ388891	TC101296
0262	AJ388893	TC104756
0263	AJ388894	TC102899
0265	AJ388896	singleton
0266	AJ388897	TC107193
0267	AJ388898	TC94436

0268	AJ388899	singleton
0270	AJ388901	TC96245
0271	AJ388902	singleton
0273	AJ388904	singleton
0274	AJ388905	TC106899
0276	AJ388907	TC98590
0277	AJ388908	TC103041
0278	AJ388909	TC101043
0279	AJ388910	TC94903
0280	AJ388911	TC96054
0281	AJ388912	singleton
0282	AJ389054	TC96916
0283	AJ388913	TC94647
0284	AJ388914	TC101000
0285	AJ388915	singleton
0286	AJ388916	singleton
0287	AJ388917	TC93931
0288	AJ388918	TC94518
0289	AJ388919	TC94581
0290	AJ388920	TC106541
0291	AJ388921	TC107758
0292	AJ388922	TC95339
0294	AJ388924	singleton
0295	AJ388925	TC93919
0296	AJ388926	TC94681
0299	AJ388929	TC93945
0300	AJ388930	TC101898
0301	AJ388931	TC100213
0303	AJ388933	TC95563
0304	AJ388934	TC107718
0305	AJ388935	TC101268
0307	AJ388937	singleton
0308	AJ388938	TC94446
0309	AJ388939	TC94515
0310	AJ388940	TC106692
0312	AJ388941	TC94540
0313	AJ388942	TC95881
0314	AJ388943	TC101797
0315	AJ388944	singleton
0316	AJ388945	TC110546
0317	AJ388946	TC101623
0319	AJ388948	TC94718
0320	AJ388949	TC106716
0321	AJ389055	TC102185
0323	AJ388950	singleton
0324	AJ388951	TC109055
0325	AJ388952	singleton
0326	AJ388953	TC106500
0327	AJ388954	TC94360
0329	AJ388956	TC96120
0330	AJ388957	TC94053
0331	AJ388958	TC106347
0332	AJ388959	TC94874
0333	AJ388960	singleton
0334	AJ388961	TC107273
0337	AJ388964	TC94209

0338	AJ388965	TC106866
0340	AJ388967	TC112150
0341	AJ388968	TC94268
0342	AJ388969	TC94980
0343	AJ388970	TC102979
0344	AJ388971	TC101807
0345	AJ388972	TC100879
0346	AJ388973	TC94061
0347	AJ388974	singleton
0348	AJ388975	TC93950
0349	AJ388976	TC106459
0350	AJ388977	TC111704
0352	AJ388978	TC108003
0353	AJ388979	TC106697
0356	AJ388981	singleton
0357	AJ388982	TC103108
0358	AJ388983	TC106640
0359	AJ388984	singleton
0360	AJ388985	TC109579
0361	AJ388986	TC101066
0362	AJ388987	TC99272
0363	AJ388988	TC106384
0364	AJ388989	TC95314
0365	AJ388990	TC104053
0366	AJ388991	TC102128
0367	AJ388992	TC94919
0368	AJ388993	TC110787
0371	AJ388996	TC106667
0374	AJ388998	TC106741
0375	AJ388999	TC106743
0376	AJ389000	singleton
0377	AJ389001	TC94278
0378	AJ389002	TC94131
0379	AJ389003	TC110894
0382	AJ389005	TC94293
0383	AJ389006	TC94333
0384	AJ389007	singleton
0385	AJ389008	TC102614
0386	AJ389009	singleton
0387	AJ389010	TC94886
0389	AJ389012	TC106896
0390	AJ389013	singleton
0391	AJ389014	TC108193
0392	AJ389015	TC101100
0393	AJ389016	TC98625
0394	AJ389017	singleton
0395	AJ389018	TC100271
0397	AJ389020	TC99453
0398	AJ389021	TC94101
0399	AJ389022	TC101865
0400	AJ389023	singleton
0401	AJ389024	singleton
0402	AJ389025	TC108392
0403	AJ389026	TC106457
0404	AJ389027	TC107252
0405	AJ389028	TC108603

0406	AJ389029	TC100979
0407	AJ389030	TC94770
0408	AJ389031	singleton
0409	AJ389032	TC110056
0410	AJ389033	TC100580
0411	AJ389034	TC101039
0412	AJ389035	TC97140
0413	AJ389036	TC106312
0414	AJ389037	TC105276
0415	AJ389038	TC95565
0416	AJ389039	TC100146
0417	AJ389040	TC107133
0418	AJ389041	TC100851
0419	AJ389042	TC109830
0421	AJ389044	TC106948
0423	AJ389046	TC101728
0424	AJ389047	TC98386
0425	AJ389048	TC106582
0426	AJ389049	TC95421
0501	DY615432	singleton
0502	DY615433	TC97828
0503	DY615434	singleton
0505	DY615436	TC100731
0506	DY615437	TC101825
0507	DY615438	singleton
0508	DY615439	TC110922
0509	DY615440	TC108181
0510	DY615441	TC108327
0511	DY615442	TC100777
0512	DY615443	TC96206
0513	DY615444	TC102768
0514	DY615445	TC101236
0515	DY615446	singleton
0516	DY615447	TC98206
0517	DY615448	TC95953
0518	DY615449	TC109790
0519	DY615450	singleton
0520	DY615451	singleton
0521	DY615452	TC109798
0522	DY615453	TC108015
0523	DY615454	TC103969
0524	DY615455	TC105324
0525	DY615456	TC101346
0526	DY615457	TC95560
0527	DY615458	TC107324
0528	DY615459	TC95450
0529	DY615460	TC100726
0531	DY615461	TC96606
0532	DY615462	TC101980
0533	DY615463	TC102255
0535	DY615464	singleton
0536	DY615465	singleton
0537	DY615466	TC97253
0538	DY615467	singleton
0540	DY615469	singleton
0541	DY615470	singleton

0542	DY615471	TC109231
0543	DY615472	TC102189
0544	DY615473	TC100522
0545	DY615474	TC100133
0546	DY615475	TC93918
0547	DY615476	TC106633
0550	DY615479	singleton
0551	DY615480	singleton
0552	DY615481	singleton
0553	DY615482	singleton
0555	DY615483	singleton
0559	DY615485	singleton
0560	DY615486	TC97930
0561	DY615487	TC94532
0566	DY615490	singleton
0568	DY615491	singleton
0570	DY615492	singleton
0571	DY615493	TC111268
0574	DY615495	TC108738
0575	DY615496	TC94382
0577	DY615497	singleton
0578	DY615498	TC101857
0579	DY615499	singleton
0580	DY615500	TC98374
0581	DY615501	singleton
0582	DY615502	singleton
0583	DY615503	TC106770
0585	DY615505	TC107287
0587	DY615507	TC106613
0589	DY615508	TC100398
0592	DY615510	singleton
0593	DY615511	singleton
0594	DY615512	singleton
0595	DY615513	singleton
0596	DY615514	singleton
0597	DY615515	singleton
0598	DY615516	singleton
0600	DY615517	singleton
0601	DY615518	singleton
0602	DY615519	TC107729
0603	DY615520	TC106737
0604	DY615521	TC109445
0605	DY615522	TC94313
0610	DY615527	TC107071
0611	DY615528	TC107232
0612	DY615529	TC108612
0613	DY615530	TC106801
0614	DY615531	TC101686
0615	DY615532	TC103209
0616	DY615533	TC99189
0617	DY615534	TC107964
0618	DY615535	singleton
0619	DY615536	TC107397
0620	DY615537	TC99623
0621	DY615538	singleton
0622	DY615539	singleton

0627	DY615544	TC100652
0628	DY615545	TC106787
0629	DY615546	TC108841
0630	DY615547	TC101167
0631	DY615548	TC107529
0632	DY615549	singleton
0634	DY615551	TC107119
0635	DY615552	TC103164
0638	DY615555	TC103399
0639	DY615556	TC98184
0640	DY615557	TC93936
0641	DY615558	singleton
0642	DY615559	TC94498
0643	DY615560	TC101756
0645	DY615562	TC108130
0646	DY615563	TC97752
0647	DY615564	TC95129
0648	DY615565	TC94983
0651	DY615568	TC94335
0652	DY615569	TC97596
0655	DY615572	TC103668
0661	DY615577	TC102148
0662	DY615578	TC94281
0664	DY615580	singleton
0665	DY615581	singleton
0666	DY615582	singleton
0676	DY615592	singleton
0677	DY615593	singleton
0678	DY615594	singleton
0679	DY615595	TC102608
0683	DY615599	TC100555
0687	DY615603	TC100754
0691	DY615607	TC97355
0694	DY615610	TC106521
0698	DY615614	TC109386
0699	DY615615	singleton
0700	DY615616	TC107231
0701	DY615617	TC102794
0703	DY615618	TC100267
0706	DY615621	singleton
0707	DY615622	singleton
0709	DY615624	singleton
0711	DY615626	TC100524
0713	DY615628	singleton
0715	DY615630	TC99176
0716	DY615631	TC109420
0717	DY615632	singleton
0719	DY615634	singleton
0720	DY615635	TC96968
0721	DY615636	TC108572
0724	DY615638	TC108661
0725	DY615639	TC103326
0726	DY615640	TC104135
0727	DY615641	TC94762
0728	DY615642	TC94065
0729	DY615643	TC106519

0731	DY615644	TC101123
0732	DY615645	TC106677
0733	DY615646	TC108679
0737	DY615650	TC106991
0738	DY615651	singleton
0740	DY615653	TC106922
0742	DY615655	singleton
0745	DY615658	singleton
0747	DY615660	singleton
0748	DY615661	TC95586
0750	DY615663	TC106986
0754	DY615666	TC109594
0755	DY615667	TC102600
0756	DY615668	TC106746
0759	DY615671	TC95443
0760	DY615672	TC102365
0761	DY615673	TC96004
0763	DY615675	TC100786
0764	DY615676	TC112080
0768	DY615680	TC93944
0769	DY615681	TC106497
0800	DY615685	singleton
0802	DY615687	singleton
0804	DY615689	TC108851
0805	DY615690	TC112013
0811	DY615696	singleton
0812	DY615697	singleton
0813	DY615698	singleton
0814	DY615699	TC100549
0817	DY615702	singleton
0818	DY615703	TC100635
0823	DY615708	singleton
0827	DY615711	TC98231
0829	DY615713	TC109393
0830	DY615714	TC100579
1001	DY615715	TC106342
1002	DY615716	TC108339
1003	DY615717	TC109470
1004	DY615718	singleton
1005	DY615719	TC93929
1006	DY615720	TC106631
1008	DY615721	TC99808
1009	DY615722	TC112251
1010	DY615723	singleton
1011	DY615724	singleton
1012	DY615725	TC94357
1013	DY615726	singleton
1015	DY615727	TC106518
1018	DY615729	TC100483
1022	DY615731	TC96186
1023	DY615732	TC100575
1025	DY615733	TC107058
1028	DY615734	TC95507
1029	DY615735	TC108463
1030	DY615736	singleton
1032	DY615737	TC106753

1033	DY615738	TC101115
1035	DY615739	TC106528
1036	DY615740	TC94329
1037	DY615741	TC100515
1040	DY615744	TC106772
1042	DY615745	TC100681
1043	DY615746	TC109580
1044	DY615747	TC106919
1045	DY615748	TC100369
1050	DY615751	TC108488
1051	DY615752	TC95076
1052	DY615753	TC106429
1053	DY615754	TC104290
1056	DY615755	singleton
1059	DY615757	TC101527
1060	DY615758	TC100708
1065	DY615759	TC100533
1067	DY615761	TC100463
1071	DY615763	TC106587
1072	DY615764	TC94961
1075	DY615765	TC101544
1080	DY615766	TC101701
1083	DY615767	TC110997
1084	DY615768	TC100139
1085	DY615769	TC100778
1086	DY615770	TC94693
1092	DY615772	TC106404
1093	DY615773	TC100147
1095	DY615775	TC102653
1097	DY615776	TC102378
1099	DY615778	TC95756
1101	DY615780	TC100155
1103	DY615782	singleton
1105	DY615783	TC106745
1106	DY615784	TC105505
1107	DY615785	singleton
1108	DY615786	TC95643
1109	DY615787	TC110467
1110	DY615788	TC100696
1113	DY615790	TC101191
1114	DY615791	TC94539
1115	DY615792	TC106434
1116	DY615793	singleton
1118	DY615795	TC101372
1121	DY615797	TC103583
1122	DY615798	TC107005
1123	DY615799	TC111767
1125	DY615800	TC94982
1126	DY615801	singleton
1127	DY615802	TC97726
1128	DY615803	singleton
1129	DY615804	TC102042
1132	DY615806	TC100814
1133	DY615807	TC94897
1134	DY615808	TC106645
1135	DY615809	TC94940

1138	DY615811	TC100604
1140	DY615813	singleton
1141	DY615814	TC94364
1143	DY615815	singleton
1144	DY615816	TC107786
1145	DY615817	TC103042
1147	DY615818	TC106512
1150	DY615820	singleton
1151	DY615821	TC97327
1153	DY615822	TC106494
1154	DY615823	TC101209
1155	DY615824	TC93927
1158	DY615826	TC95195
1159	DY615827	TC107600
1161	DY615828	singleton
1163	DY615829	TC93952
1164	DY615830	TC101653
1165	DY615831	TC100650
1166	DY615832	TC100976
1167	DY615833	TC108160
1168	DY615834	TC109642
1169	DY615835	TC106627
1170	DY615836	TC94135
1171	DY615837	TC100692
1172	DY615838	TC95472
1173	DY615839	singleton
1174	DY615840	TC100478
1175	DY615841	TC100137
1176	DY615842	TC107712
1177	DY615843	TC106995
1178	DY615844	TC104784
1179	DY615845	singleton
1180	DY615846	singleton
1181	DY615847	singleton
1182	DY615848	TC99419
1184	DY615849	TC96252
1185	DY615850	TC94780
1187	DY615851	TC106751
1188	DY615852	TC95427
1189	DY615853	singleton
1191	DY615854	TC108403
1192	DY615855	TC104742
1193	DY615856	TC100168
1195	DY615857	singleton
1196	DY615858	singleton
1198	DY615859	TC100276
1199	DY615860	TC101253
1200	DY615861	TC108755
1202	DY615862	TC94347
1204	DY615864	singleton
1205	DY615865	singleton
1207	DY615867	TC95172
1208	DY615868	TC110704
1210	DY615870	TC95180
1212	DY615872	singleton
1213	DY615873	TC96083

1215	DY615874	TC95129
1216	DY615875	TC102656
1218	DY615877	TC106831
1219	DY615878	singleton
1220	DY615879	TC111259
1223	DY615882	TC106826
1224	DY615883	TC100943
1226	DY615885	TC94621
1227	DY615886	TC107530
1228	DY615887	TC95930
1229	DY615888	TC107521
1232	DY615889	TC96933
1233	DY615890	TC107746
1234	DY615891	singleton
1236	DY615893	TC103178
1239	DY615894	TC100310
1240	DY615895	TC100178
1241	DY615896	TC107567
1242	DY615897	singleton
1243	DY615898	singleton
1244	DY615899	TC94452
1245	DY615900	TC106440
1248	DY615903	TC108608
1250	DY615905	TC100233
1251	DY615906	TC108979
1253	DY615907	TC101763
1254	DY615908	singleton
1255	DY615909	TC101113
1256	DY615910	TC94956
1258	DY615912	TC106105
1260	DY615914	TC109792
1261	DY615915	TC100411
1262	DY615916	TC108269
1263	DY615917	TC100136
1264	DY615918	singleton
1265	DY615919	TC108606
1266	DY615920	TC94275
1267	DY615921	TC106698
1269	DY615923	TC106650
1270	DY615924	TC109457
1271	DY615925	TC107444
1272	DY615926	singleton
1273	DY615927	TC108373
1274	DY615928	TC107117
1276	DY615930	TC107772
1277	DY615931	TC97495
1278	DY615932	TC95087
1279	DY615933	TC107583
1282	DY615935	TC103726
1284	DY615937	TC107268
1285	DY615938	TC100606
1287	DY615940	singleton
1288	DY615941	TC94406
1289	DY615942	singleton
1290	DY615943	singleton
1291	DY615944	singleton

1292	DY615945	TC109935
1293	DY615946	TC95250
1294	DY615947	TC94793
1295	DY615948	TC94064
1296	DY615949	TC100834
1297	DY615950	TC108792
1298	DY615951	singleton
1300	DY615953	TC106715
1301	DY615954	TC104041
1304	DY615956	TC94304
1305	DY615957	TC106747
1308	DY615959	TC107182
1316	DY615961	TC95321
1319	DY615962	TC107322
1322	DY615963	singleton
1324	DY615965	TC94425
1325	DY615966	singleton
1326	DY615967	TC97494
1327	DY615968	TC100541
1329	DY615970	TC106644
1332	DY615972	singleton
1333	DY615973	TC101558
1340	DY615974	TC108905
1341	DY615975	TC106516
1342	DY615976	TC93948
1343	DY615977	TC100807
1352	DY615979	singleton
1354	DY615980	TC111083
1357	DY615982	TC107669
1358	DY615983	TC111751
1360	DY615985	TC95551
1362	DY615986	TC95054
1363	DY615987	TC99503
1365	DY615988	TC106941
1366	DY615989	TC93959
1367	DY615990	TC100391
1368	DY615991	TC94789
1371	DY615994	TC101347
1372	DY615995	TC109033
1373	DY615996	singleton
1377	DY615997	TC96231
1379	DY615998	TC106595
1380	DY615999	TC100440
1381	DY616000	singleton
1383	DY616001	TC98647
1384	DY616002	singleton
1385	DY616003	TC100148
1387	DY616004	TC96166
1390	DY616006	singleton
1391	DY616007	singleton
1392	DY616008	TC100610
1393	DY616009	TC106618
1394	DY616010	TC94935
1395	DY616011	singleton
1396	DY616012	TC94672
1398	DY616013	TC94680

1399	DY616014	singleton
1401	DY616016	singleton
1402	DY616017	TC109480
1404	DY616019	TC106799
1405	DY616020	TC101284
1409	DY616022	TC100159
1410	DY616023	TC107514
1411	DY616024	TC94398
1412	DY616025	singleton
1413	DY616026	TC106362
1414	DY616027	TC106536
1416	DY616029	TC106336
1418	DY616030	singleton
1419	DY616031	TC94190
1420	DY616032	singleton
1421	DY616033	TC106485
1422	DY616034	singleton
1424	DY616036	singleton
1425	DY616037	TC108312
1426	DY616038	TC100402
1428	DY616039	TC94398
1429	DY616040	singleton
1430	DY616041	TC106929
1431	DY616042	TC100990
1432	DY616043	singleton
1434	DY616045	TC106399
1435	DY616046	TC100767
1436	DY616047	TC97207
1437	DY616048	TC103632
1438	DY616049	TC103276
1439	DY616050	singleton
1440	DY616051	TC100212
1441	DY616052	TC94614
1444	DY616055	TC95391
1446	DY616057	TC100761
1447	DY616058	TC100557
1448	DY616059	TC106328
1449	DY616060	singleton
1451	DY616062	singleton
1452	DY616063	singleton
1453	DY616064	TC108149
1454	DY616065	singleton
1455	DY616066	singleton
1456	DY616067	TC97086
1460	DY616070	TC106720
1461	DY616071	TC94230
1464	DY616074	singleton
1466	DY616075	singleton
1467	DY616076	TC101141
1468	DY616077	TC100383
1469	DY616078	singleton
1472	DY616080	TC100719
1474	DY616082	TC94015
1475	DY616083	TC107277
1476	DY616084	singleton
1477	DY616085	singleton

1479	DY616086	TC101826
1481	DY616088	TC100270
1482	DY616089	TC96982
1483	DY616090	singleton
1484	DY616091	TC95493
1489	DY616094	TC94921
1490	DY616095	TC94876
1491	DY616096	TC107045
1492	DY616097	TC107828
1493	DY616098	TC100222
1494	DY616099	TC100408
1495	DY616100	singleton
1497	DY616102	TC95961
1498	DY616103	TC100151
1499	DY616104	singleton
1500	DY616105	TC101307
1501	DY616106	singleton
1502	DY616107	TC95912
1503	DY616108	singleton
1505	DY616109	TC94867
1506	DY616110	TC109433
1509	DY616113	TC107441
1511	DY616114	TC110419
1514	DY616117	TC98461
1516	DY616119	TC96791
1517	DY616120	TC100427
1518	DY616121	TC93989
1519	DY616122	TC107830
1521	DY616123	TC100410
1522	DY616124	TC93993
1526	DY616127	TC100868
1528	DY616129	TC94722
1529	DY616130	TC107406
1530	DY616131	TC100802
1531	DY616132	singleton
1532	DY616133	TC108128
1533	DY616134	TC106408
1534	DY616135	TC107340
1535	DY616136	singleton
1536	DY616137	TC108346
1538	DY616138	TC96468
1540	DY616139	TC101252
1541	DY616140	TC106316
1542	DY616141	TC106474
1544	DY616143	singleton
1547	DY616145	TC100178
1548	DY616146	singleton
1549	DY616147	TC106342
1550	DY616148	TC100647
1552	DY616150	TC99490
1555	DY616152	TC109169
1558	DY616154	TC93991
1560	DY616156	TC100746
1561	DY616157	singleton
1562	DY616158	TC94661
1563	DY616159	singleton

1565	DY616161	TC100742
1567	DY616162	TC108604
1568	DY616163	singleton
1573	DY616167	TC95706
1574	DY616168	TC102356
1575	DY616169	TC100854
1576	DY616170	TC95086
1577	DY616171	TC93977
1578	DY616172	singleton
1579	DY616173	singleton
1581	DY616175	TC103292
1587	DY616178	TC94104
1588	DY616179	singleton
1589	DY616180	TC107188
1591	DY616181	TC100208
1595	DY616182	TC102302
1596	DY616183	TC98572
1599	DY616184	singleton
1600	DY616185	TC101118
1604	DY616187	TC109373
1608	DY616189	TC96169
1609	DY616190	TC95169
1612	DY616191	TC107365
1613	DY616192	TC95887
1623	DY616199	TC101219
1626	DY616201	TC101824
1627	DY616202	TC108345
1629	DY616203	singleton
1630	DY616204	singleton
1633	DY616205	TC93947
1634	DY616206	TC105954
1635	DY616207	TC104158
1638	DY616208	singleton
1641	DY616209	TC106965
1643	DY616210	TC107809
1645	DY616211	singleton
1647	DY616212	TC97772
1648	DY616213	singleton
1650	DY616215	TC102472
1651	DY616216	singleton
1652	DY616217	TC103873
1653	DY616218	singleton
1654	DY616219	TC106560
1655	DY616220	TC100446
1656	DY616221	TC95298
1658	DY616222	TC108598
1662	DY616224	TC95109
1665	DY616226	singleton
1668	DY616229	TC101355
1670	DY616230	singleton
1671	DY616231	singleton
1672	DY616232	TC107129
1674	DY616233	TC96091
1675	DY616234	TC107228
1676	DY616235	TC96179
1678	DY616236	TC94645

1683	DY616239	singleton
1684	DY616240	TC107656
1685	DY616241	TC108779
1686	DY616242	singleton
1687	DY616243	TC101298
1688	DY616244	TC106719
1691	DY616245	singleton
1692	DY616246	singleton
1695	DY616249	TC100671
1698	DY616250	TC94096
1699	DY616251	TC108011
1700	DY616252	TC100779
1701	DY616253	TC96544
1702	DY616254	TC107031
1704	DY616256	TC107769
1714	DY616260	TC100540
1715	DY616261	TC102115
1717	DY616262	singleton
1719	DY616263	TC101218
1720	DY616264	singleton
1721	DY616265	TC95535
1722	DY616266	singleton
1723	DY616267	singleton
1725	DY616268	TC101029
1726	DY616269	TC98404
1728	DY616270	TC108081
1729	DY616271	TC106311
1730	DY616272	TC100668
1731	DY616273	singleton
1733	DY616275	TC107908
1734	DY616276	TC94517
1738	DY616279	TC94058
1739	DY616280	TC103815
1740	DY616281	singleton
1741	DY616282	TC101289
1744	DY616283	TC94252
1746	DY616284	TC106523
1748	DY616285	TC94348
1761	DY616297	TC99440
1764	DY616300	TC96701
1768	DY616304	singleton
1792	DY616325	TC106884
1795	DY616328	TC109740
1798	DY616330	TC106748
1801	DY616333	singleton
1802	DY616334	TC106560
1803	DY616335	TC94474
1805	DY616337	singleton
1806	DY616338	TC95916
1807	DY616339	TC106729
1810	DY616342	TC102486
1811	DY616343	TC110797
1812	DY616344	TC106777
1813	DY616345	TC95233
1815	DY616346	TC106674
1816	DY616347	TC109065

1821	DY616350	singleton
1822	DY616351	TC110221
1824	DY616353	TC94526
1825	DY616354	singleton
1827	DY616356	TC102274
1828	DY616357	TC102718
1832	DY616359	TC96796
1834	DY616361	TC100830
1837	DY616363	TC101753
1841	DY616367	TC103688
1842	DY616368	TC107630
1844	DY616370	TC106334
1846	DY616372	TC106598
1847	DY616373	singleton
1848	DY616374	singleton
1849	DY616375	singleton
1850	DY616376	TC97284
1851	DY616377	TC95157
1852	DY616378	TC101456
1853	DY616379	TC102497
1854	DY616380	TC102924
1858	DY616384	TC94194
1861	DY616386	singleton
1864	DY616387	TC100894
1870	DY616389	TC95385
1871	DY616390	TC95002
1872	DY616391	TC103477
1873	DY616392	TC104329
1875	DY616393	TC101752
1876	DY616394	TC107068
1878	DY616396	TC101103
1883	DY616399	TC100590
1884	DY616400	TC94919
1888	DY616403	singleton
1889	DY616404	singleton
1890	DY616405	TC103322
1892	DY616407	TC110605
1894	DY616408	TC107295
1895	DY616409	TC100203
1899	DY616413	TC107374
1900	DY616414	TC99595
1902	DY616416	singleton
1904	DY616418	TC98974
1905	DY616419	TC94752
1908	DY616421	singleton
1909	DY616422	TC102605
1911	DY616423	TC101230
1912	DY616424	TC107654
1914	DY616426	TC98752
1915	DY616427	TC95407
1916	DY616428	TC99791
1917	DY616429	singleton
1918	DY616430	TC107025
1919	DY616431	TC107494
1920	DY616432	TC94137
1921	DY616433	TC94289

1923	DY616434	TC106791
1925	DY616436	TC105580
1926	DY616437	TC100470
1927	DY616438	TC107201
1928	DY616439	TC106904
1929	DY616440	TC100769
1934	DY616443	TC107783
1935	DY616444	TC111473
1936	DY616445	TC107124
1939	DY616447	TC95128
1940	DY616448	TC95445
1941	DY616449	TC101529
1946	DY616452	TC95420
1948	DY616454	TC100667
1949	DY616455	TC111807
1950	DY616456	TC100709
1951	DY616457	TC110643
1952	DY616458	TC95079
1953	DY616459	singleton
1954	DY616460	singleton
1957	DY616462	singleton
1958	DY616463	TC112406
1960	DY616464	TC107422
2004	DY616498	TC103370
2005	DY616499	TC106711
2006	DY616500	TC105194
2007	DY616501	TC107073
2008	DY616502	singleton
2013	DY616505	TC108933
2014	DY616506	TC94610
2016	DY616507	singleton
2019	DY616509	TC98799
2020	DY616510	TC105779
2025	DY616511	TC95495
2026	DY616512	TC106691
2032	DY616514	TC107107
2035	DY616516	TC102015
2036	DY616517	TC95037
2037	DY616518	TC101259
2038	DY616519	TC97021
2040	DY616521	TC97438
2041	DY616522	TC100761
2042	DY616523	TC94334
2044	DY616525	TC106656
2048	DY616528	TC108691
2049	DY616529	TC98608
2050	DY616530	TC101128
2051	DY616531	TC111920
2052	DY616532	TC101558
2054	DY616533	TC95175
2057	DY616535	TC94971
2063	DY616538	singleton
2065	DY616540	TC101943
2067	DY616542	singleton
2068	DY616543	TC106622
2069	DY616544	TC100773

2074	DY616548	TC94745
2076	DY616550	TC94802
2077	DY616551	singleton
2078	DY616552	TC106337
2079	DY616553	singleton
2080	DY616554	TC108714
2081	DY616555	singleton
2082	DY616556	TC94702
2083	DY616557	TC101444
2084	DY616558	TC107252
2086	DY616560	singleton
2087	DY616561	TC107833
2088	DY616562	TC95035
2090	DY616563	singleton
2091	DY616564	TC95127
2092	DY616565	TC94349
2094	DY616566	TC94228
2096	DY616568	TC110870
2097	DY616569	singleton
2098	DY616570	TC101764
2099	DY616571	TC103427
2100	DY616572	TC106814
2101	DY616573	TC109412
2102	DY616574	singleton
2105	DY616577	TC95710
2106	DY616578	TC101679
2108	DY616580	TC94953
2109	DY616581	TC95439
2112	DY616583	TC106330
2113	DY616584	TC100408
2115	DY616585	TC101418
2118	DY616588	TC98616
2119	DY616589	TC100149
2120	DY616590	TC101639
2124	DY616594	singleton
2126	DY616596	TC111120
2127	DY616597	TC108062
2131	DY616599	TC94329
2134	DY616602	TC107303
2139	DY616604	TC101262
2140	DY616605	singleton
2142	DY616607	TC106751
2143	DY616608	singleton
2146	DY616611	singleton
2147	DY616612	TC100210
2148	DY616613	singleton
2149	DY616614	TC107375
2150	DY616615	TC95444
2151	DY616616	TC94902
2153	DY616618	TC106701
2154	DY616619	TC109630
2155	DY616620	TC96274
2156	DY616621	singleton
2157	DY616622	singleton
2159	DY616624	TC106530
2160	DY616625	TC94238

2161	DY616626	TC107577
2162	DY616627	TC107574
2163	DY616628	TC96256
2164	DY616629	TC96436
2166	DY616631	TC94454
2168	DY616632	TC100532
2169	DY616633	TC93921
2170	DY616634	TC94181
2172	DY616636	TC95264
2173	DY616637	TC94379
2174	DY616638	singleton
2175	DY616639	TC101023
2176	DY616640	TC107843
2177	DY616641	TC100753
2178	DY616642	TC100770
2180	DY616643	TC95517
2187	DY616646	TC107737
2188	DY616647	TC101882
2189	DY616648	TC94795
2191	DY616649	TC100314
2194	DY616650	TC102101
2195	DY616651	singleton
2196	DY616652	TC106441
2197	DY616653	TC95576
2198	DY616654	TC106377
2199	DY616655	TC100488
2200	DY616656	TC95688
2202	DY616658	singleton
2204	DY616659	TC108059
2206	DY616661	singleton
2207	DY616662	TC104949
2208	DY616663	TC107728
2209	DY616664	TC109465
2210	DY616665	TC106952
2211	DY616666	TC95147
2212	DY616667	singleton
2213	DY616668	TC101073
2215	DY616670	TC110231
2216	DY616671	TC104784
2217	DY616672	TC94423
2218	DY616673	TC106724
2219	DY616674	TC101889
2220	DY616675	singleton
2222	DY616676	TC95758
2224	DY616677	TC97557
2225	DY616678	TC95493
2227	DY616679	TC109982
2228	DY616680	TC112390
2229	DY616681	TC100561
2231	DY616682	TC104856
2237	DY616687	TC107772
2238	DY616688	TC106354
2239	DY616689	TC108357
2242	DY616691	TC107235
2243	DY616692	TC100674
2246	DY616693	TC95835

2248	DY616694	TC100291
2249	DY616695	TC103993
2250	DY616696	TC100615
2251	DY616697	TC106807
2254	DY616698	TC107103
2256	DY616699	TC101081
2257	DY616700	singleton
2259	DY616702	TC107018
2260	DY616703	TC103513
2261	DY616704	TC101506
2262	DY616705	TC96734
2263	DY616706	TC103735
2265	DY616707	TC95848
2266	DY616708	TC107236
2268	DY616709	TC94934
2270	DY616710	TC94702
2271	DY616711	TC96131
2272	DY616712	TC102832
2275	DY616714	TC99100
2276	DY616715	TC106968
2279	DY616717	singleton
2282	DY616720	TC96223
2283	DY616721	singleton
2286	DY616722	TC104365
2287	DY616723	TC111649
2288	DY616724	TC108034
2289	DY616725	TC101590
2290	DY616726	TC109454
2292	DY616727	TC103802
2296	DY616730	TC101971
2299	DY616731	TC101314
2302	DY616733	TC97740
2305	DY616734	TC100409
2310	DY616735	TC109783
2315	DY616736	TC106503
2316	DY616737	TC108835
2317	DY616738	TC108455
2319	DY616739	TC100876
2320	DY616740	TC106642
2321	DY616741	TC98248
2322	DY616742	singleton
2325	DY616743	TC95426
2326	DY616744	TC111306
2327	DY616745	TC99787
2328	DY616746	TC107837
2330	DY616748	TC96352
2331	DY616749	singleton
2332	DY616750	singleton
2333	DY616751	TC101174
2334	DY616752	singleton
2335	DY616753	TC101297
2336	DY616754	TC94011
2337	DY616755	TC106452
2338	DY616756	singleton
2339	DY616757	TC100435
2341	DY616758	TC103086

2342	DY616759	TC100706
2343	DY616760	TC101470
2344	DY616761	TC102092
2348	DY616764	TC106346
2350	DY616765	TC97485
2351	DY616766	TC107254
2352	DY616767	TC102816
2354	DY616769	singleton
2355	DY616770	singleton
2356	DY616771	TC97699
2357	DY616772	singleton
2358	DY616773	TC100753
2359	DY616774	singleton
2360	DY616775	TC106367
2362	DY616776	singleton
2363	DY616777	TC94492
2364	DY616778	TC101216
2365	DY616779	TC103708
2367	DY616780	TC100335
2368	DY616781	TC100151
2369	DY616782	TC106845
2373	DY616784	TC107459
2374	DY616785	TC101595
2376	DY616786	TC95075
2379	DY616789	TC107456
2383	DY616791	TC106707
2387	DY616792	TC102193
2388	DY616793	TC102918
2392	DY616796	TC94128
2393	DY616797	TC101281
2394	DY616798	TC95833
2395	DY616799	TC112392
2399	DY616801	singleton
2403	DY616804	TC108323
2404	DY616805	TC111851
2407	DY616808	TC103563
2408	DY616809	TC101217
2409	DY616810	TC105179
2410	DY616811	TC95512
2411	DY616812	singleton
2412	DY616813	singleton
2413	DY616814	TC106920
2414	DY616815	TC103149
2416	DY616816	singleton
2422	DY616820	singleton
2423	DY616821	TC100662
2424	DY616822	TC95098
2425	DY616823	TC101237
2426	DY616824	TC95256
2428	DY616826	TC94295
2431	DY616828	TC107634
2432	DY616829	singleton
2439	DY616831	TC106855
2441	DY616832	TC100199
2447	DY616834	TC104501
2449	DY616835	TC100744

2453	DY616836	TC94302
2456	DY616837	TC109032
2458	DY616839	singleton
2459	DY616840	TC103670
2461	DY616841	TC100321
2463	DY616843	singleton
2465	DY616845	singleton
2468	DY616846	singleton
2470	DY616848	TC107091
2471	DY616849	TC100165
2472	DY616850	TC106874
2483	DY616858	TC100515
2484	DY616859	singleton
2485	DY616860	singleton
2486	DY616861	TC100179
2491	DY616864	TC93965
2492	DY616865	TC102085
2493	DY616866	TC108788
2494	DY616867	singleton
2498	DY616870	TC108234
2500	DY616872	TC107110
2501	DY616873	TC110647
2502	DY616874	singleton
2503	DY616875	TC96368
2504	DY616876	TC100847
2505	DY616877	TC106736
2509	DY616878	singleton
2510	DY616879	singleton
2511	DY616880	TC94471
2512	DY616881	TC99653
2513	DY616882	singleton
2514	DY616883	TC106714
2515	DY616884	TC108792
2516	DY616885	TC109197
2517	DY616886	singleton
2520	DY616888	TC101160
2522	DY616889	TC98931
2523	DY616890	singleton
2525	DY616891	TC102114
2530	DY616895	TC100502
2535	DY616898	singleton
2539	DY616901	TC100701
2540	DY616902	singleton
2541	DY616903	singleton
2542	DY616904	singleton
2544	DY616906	singleton
2545	DY616907	TC97955
2547	DY616908	singleton
2548	DY616909	singleton
2551	DY616910	TC96755
2553	DY616912	TC107443
2561	DY616917	singleton
2562	DY616918	TC94486
2565	DY616920	TC106518
2566	DY616921	TC94341
2568	DY616922	TC100718

2569	DY616923	TC106418
2572	DY616924	singleton
2574	DY616925	TC107581
2577	DY616926	singleton
2578	DY616927	TC100561
2579	DY616928	singleton
2581	DY616929	TC97251
2582	DY616930	TC94850
2584	DY616932	TC101066
2585	DY616933	TC101142
2588	DY616935	TC94400
2589	DY616936	TC101024
2590	DY616937	TC94500
2591	DY616938	singleton
2592	DY616939	singleton
2595	DY616942	TC100824
2596	DY616943	TC107533
2597	DY616944	TC100588
2598	DY616945	TC94282
2601	DY616946	singleton
2602	DY616947	singleton
2605	DY616950	TC107083
2608	DY616952	TC108328
2609	DY616953	TC108909
2610	DY616954	TC100565
2611	DY616955	TC110306
2612	DY616956	TC97025
2615	DY616957	TC107035
2616	DY616958	TC104393
2617	DY616959	TC93926
2618	DY616960	TC97791
2619	DY616961	TC108156
2621	DY616963	TC106798
2622	DY616964	singleton
2623	DY616965	TC96910
2624	DY616966	TC110721
2625	DY616967	TC94674
2627	DY616969	TC102992
2630	DY616972	singleton
2631	DY616973	TC94663
2632	DY616974	TC94104
2633	DY616975	TC94600
2636	DY616977	TC107081
2638	DY616978	singleton
2639	DY616979	TC106901
2640	DY616980	TC95022
2645	DY616981	TC100558
2646	DY616982	TC100147
2647	DY616983	TC106794
2650	DY616985	TC95965
2653	DY616986	singleton
2654	DY616987	TC101392
2655	DY616988	TC111248
2656	DY616989	TC107417
2657	DY616990	TC95584
2658	DY616991	singleton

2663	DY616994	TC108442
2664	DY616995	TC94512
2665	DY616996	TC108957
2666	DY616997	singleton
2668	DY616998	singleton
2669	DY616999	TC94897
2670	DY617000	TC101429
2676	DY617002	TC94846
2679	DY617003	TC108199
2681	DY617005	TC108603
2682	DY617006	TC107731
2684	DY617008	TC100497
2686	DY617009	TC109528
2691	DY617014	TC108094
2693	DY617016	TC106660
2694	DY617017	singleton
2696	DY617019	TC100999
2698	DY617020	TC104786
2699	DY617021	TC95981
2701	DY617023	TC94264
2703	DY617025	TC94604
2706	DY617028	TC96346
2707	DY617029	TC94703
2711	DY617032	TC103043
2712	DY617033	TC95709
2713	DY617034	TC94896
2714	DY617035	TC100827
2715	DY617036	singleton
2716	DY617037	TC95106
2717	DY617038	TC107015
2718	DY617039	singleton
2719	DY617040	TC103908
2720	DY617041	TC105817
2721	DY617042	singleton
2722	DY617043	singleton
2724	DY617045	singleton
2725	DY617046	TC106753
2729	DY617050	TC94357
2730	DY617051	TC97624
2731	DY617052	TC107591
2736	DY617056	TC106909
2740	DY617059	TC94335
2742	DY617061	singleton
2743	DY617062	TC107723
2744	DY617063	TC95492
2747	DY617064	TC94823
2748	DY617065	TC96758
2749	DY617066	TC100519
2751	DY617068	TC100767
2752	DY617069	singleton
2754	DY617071	singleton
2755	DY617072	TC94938
2756	DY617073	TC107009
2758	DY617074	TC107225
2762	DY617077	TC100512
2764	DY617079	singleton

2766	DY617080	TC106032
2768	DY617081	TC103102
2769	DY617082	TC96871
2771	DY617083	TC106375
2772	DY617084	TC106095
2773	DY617085	TC94023
2774	DY617086	TC106545
2775	DY617087	TC94154
2777	DY617089	TC94088
2778	DY617090	TC96682
2779	DY617091	TC101263
2782	DY617093	TC95945
2784	DY617094	TC94953
2788	DY617097	TC95638
2792	DY617101	TC103250
2794	DY617102	TC94369
2795	DY617103	TC96805
2798	DY617105	TC94365
2799	DY617106	singleton
2800	DY617107	singleton
2801	DY617108	TC104687
2805	DY617112	TC95942
2808	DY617115	TC97643
2809	DY617116	TC94689
2810	DY617117	TC106707
2812	DY617119	TC96316
2813	DY617120	TC100917
2814	DY617121	singleton
2815	DY617122	TC107756
2816	DY617123	TC94660
2818	DY617125	TC102709
2819	DY617126	singleton
2820	DY617127	TC100393
2822	DY617128	singleton
2825	DY617131	TC101443
2831	DY617136	singleton
2834	DY617139	singleton
2835	DY617140	singleton
2836	DY617141	TC100820
2837	DY617142	TC112090
2840	DY617143	TC95306
2842	DY617145	TC107674
2843	DY617146	TC95167
2850	DY617147	TC96701
2851	DY617148	TC108167
2854	DY617150	singleton
2855	DY617151	TC110063
2857	DY617153	TC104358
2862	DY617156	TC94842
2863	DY617157	singleton
2864	DY617158	singleton
2867	DY617161	TC107096
2868	DY617162	TC101780
2869	DY617163	TC100723
2873	DY617165	singleton
2882	DY617167	TC97615

2883	DY617168	TC97930
2884	DY617169	singleton
2885	DY617170	TC107174
2889	DY617172	TC100247
2896	DY617175	TC97716
2897	DY617176	TC108958
2898	DY617177	TC107210
2899	DY617178	singleton
2901	DY617179	TC106315
2903	DY617181	TC108172
2904	DY617182	TC100309
2905	DY617183	TC107773
2910	DY617186	TC105136
2911	DY617187	TC108808
2916	DY617189	TC106214
2917	DY617190	TC106470
2919	DY617192	TC101257
2921	DY617193	TC103191
2922	DY617194	singleton
2924	DY617195	TC107194
2925	DY617196	TC102991
2926	DY617197	TC107969
2927	DY617198	TC100398
2928	DY617199	TC101851
2929	DY617200	singleton
2931	DY617201	TC108861
2932	DY617202	singleton
2934	DY617203	singleton
2937	DY617204	TC94797
2940	DY617206	TC103169
2943	DY617207	TC94214
2944	DY617208	TC100833
2952	DY617209	TC109056
2953	DY617210	singleton
2961	DY617211	singleton
2964	DY617213	TC96158
2965	DY617214	TC106438
2966	DY617215	singleton
2967	DY617216	TC103144
2970	DY617217	TC96899
2972	DY617218	singleton
2974	DY617220	TC96114
2981	DY617225	TC101201
2985	DY617226	TC98832
2997	DY617230	TC106918
2999	DY617232	TC94003
3007	DY617236	TC106810
3008	DY617237	TC106629
3009	DY617238	TC111230
3013	DY617239	TC112500
3014	DY617240	singleton
3018	DY617242	TC100633
3020	DY617243	TC109073
3021	DY617244	singleton
3023	DY617245	TC103568
3024	DY617246	TC100654

3025	DY617247	TC109724
3026	DY617248	TC106726
3027	DY617249	singleton
3028	DY617250	TC103997
3029	DY617251	singleton
3031	DY617252	TC106723
3032	DY617253	TC100447
3034	DY617255	TC104243
3039	DY617256	TC101919
3040	DY617257	TC100949
3050	DY617259	singleton
3056	DY617261	TC93937
3058	DY617262	TC100613
3068	DY617265	singleton
3070	DY617267	TC107478
3072	DY617268	TC107686
3074	DY617270	TC102389
3076	DY617271	TC100785
3077	DY617272	TC109820
3080	DY617273	TC100810
3093	DY617276	TC94731
3098	DY617278	TC95783
3099	DY617279	TC106356
3101	DY617280	TC105747
3103	DY617281	singleton
3104	DY617282	TC103328
3105	DY617283	TC94546
3109	DY617284	singleton
3111	DY617286	TC100893
3114	DY617288	TC103946
3116	DY617290	TC94337
3120	DY617292	TC108864
3122	DY617294	TC95550
3123	DY617295	TC101745
3124	DY617296	TC95184
3128	DY617298	TC96367
3133	DY617300	TC107212
3135	DY617301	singleton
3137	DY617302	TC96408
3148	DY617304	TC107637
3152	DY617306	singleton
3156	DY617308	singleton
3161	DY617310	TC108295
3165	DY617311	TC108316
3166	DY617312	TC110777
3175	DY617315	TC103296
3181	DY617317	singleton
3182	DY617318	TC104117
3183	DY617319	TC101991
3191	DY617323	TC107034
3194	DY617325	TC97070
3196	DY617326	TC107742
3199	DY617328	TC100122
3202	DY617330	TC102486
3205	DY617331	singleton
3206	DY617332	TC102562

3207	DY617333	TC94534
3208	DY617334	TC95178
3210	DY617335	TC95000
3212	DY617337	singleton
3218	DY617340	TC107391
3223	DY617342	TC93928
3226	DY617344	TC100768
3227	DY617345	singleton
3229	DY617346	TC94313
3230	DY617347	singleton
3233	DY617348	TC106987
3235	DY617349	TC100041
3236	DY617350	TC95044
3239	DY617352	TC106862
3245	DY617357	singleton
3246	DY617358	singleton
3252	DY617362	singleton
3256	DY617363	TC107419
3265	DY617367	TC107635
3281	DY617369	TC95150
3284	DY617370	TC107854
3290	DY617372	TC108280
3291	DY617373	singleton
3292	DY617374	singleton
3295	DY617376	TC94712
3296	DY617377	TC98077
3301	DY617378	TC98445
3305	DY617379	singleton
3308	DY617381	TC106321
3309	DY617382	singleton
3310	DY617383	TC96456
3311	DY617384	TC105106
3313	DY617386	singleton
3317	DY617388	TC109722
3318	DY617389	TC103116
3325	DY617391	TC109930
3331	DY617392	TC103438
3334	DY617393	TC108214
3335	DY617394	TC106332
3336	DY617395	TC99999
3338	DY617397	TC101595
3339	DY617398	TC111557
3340	DY617399	TC100631
3355	DY617401	singleton
3362	DY617403	TC106463
3367	DY617406	singleton
3368	DY617407	TC101202
3370	DY617408	TC107352
3381	DY617412	TC107132
3384	DY617413	TC94631
3385	DY617414	TC100262
3386	DY617415	TC101238
3387	DY617416	singleton
3390	DY617418	TC100728
3392	DY617419	singleton
3393	DY617420	TC101408

3400	DY617421	TC95075
3402	DY617422	TC108139
3405	DY617423	singleton
3406	DY617424	singleton
3411	DY617425	TC104645
3413	DY617426	TC93941
3417	DY617429	singleton
3418	DY617430	TC110195
3420	DY617432	TC98519
3421	DY617433	TC100601
3422	DY617434	TC106985
3424	DY617436	TC95040
3425	DY617437	TC107401
3430	DY617441	TC95340
3434	DY617443	TC106865
3436	DY617445	TC97701
3438	DY617447	TC110061
3439	DY617448	TC95215
3440	DY617449	TC106496
3442	DY617450	singleton
3445	DY617452	TC100498
3451	DY617455	TC101920
3455	DY617456	TC107024
3457	DY617458	TC106060
3459	DY617459	TC109945
3461	DY617460	TC107017
3462	DY617461	TC94685
3463	DY617462	singleton
3465	DY617463	TC99512
3466	DY617464	singleton
3467	DY617465	TC107275
3468	DY617466	TC106520
3469	DY617467	TC94583
3470	DY617468	TC94796
3471	DY617469	TC109490
3475	DY617470	singleton
3478	DY617472	TC109261
3480	DY617474	TC96501
3484	DY617475	TC94732
3485	DY617476	TC106988
3488	DY617479	TC96334
3490	DY617480	TC106719
3495	DY617483	singleton
3497	DY617484	TC96184
3505	DY617487	TC95255
3508	DY617489	TC99849
3510	DY617491	TC100240
3511	DY617492	TC106685
3515	DY617493	TC107124
3520	DY617494	TC102066
3522	DY617495	TC96003
3524	DY617497	TC100144
3533	DY617501	TC94395
3534	DY617502	TC107822
3536	DY617503	TC95315
3548	DY617506	TC95382

3549	DY617507	singleton
3550	DY617508	TC96176
3552	DY617509	TC106465
3557	DY617511	TC94154
3560	DY617513	singleton
3563	DY617514	TC100931
3564	DY617515	TC101167
3579	DY617517	TC106675
3595	DY617518	TC94673
3615	DY617519	singleton
3617	DY617520	TC109432
3620	DY617521	TC97347
3623	DY617523	TC108151
3628	DY617526	TC104335
3629	DY617527	TC94045
3635	DY617530	TC108905
3636	DY617531	TC94697
3638	DY617532	TC111076
3639	DY617533	TC107876
3640	DY617534	TC108455
3643	DY617537	singleton
3647	DY617539	TC100883
3651	DY617542	singleton
3652	DY617543	TC110362
3656	DY617547	TC100257
3658	DY617548	TC107270
3659	DY617549	singleton
3660	DY617550	TC108218
3663	DY617551	TC100890
3666	DY617554	singleton
3667	DY617555	TC111030
3671	DY617558	TC95796
3674	DY617560	TC100476
3676	DY617562	TC95836
3677	DY617563	TC94513
3678	DY617564	TC97895
3679	DY617565	TC96646
3682	DY617567	singleton
3684	DY617569	TC103517
3685	DY617570	TC101893
3687	DY617571	TC96729
3688	DY617572	singleton
3694	DY617577	TC106841
3695	DY617578	TC102310
3696	DY617579	TC106752
3697	DY617580	singleton
3699	DY617582	TC95305
3700	DY617583	TC95641
3703	DY617584	singleton
3705	DY617586	TC97702
3707	DY617588	TC106727
3708	DY617589	singleton
3710	DY617591	TC101330
3713	DY617593	singleton
3717	DY617596	TC98728
3719	DY617598	TC106161

3720	DY617599	TC108764
3721	DY617600	TC94965
3722	DY617601	TC94447
3724	DY617602	singleton
3725	DY617603	TC100694
3731	DY617606	TC101834
3732	DY617607	singleton
3735	DY617609	TC94760
3744	DY617614	TC107320
3746	DY617615	TC95470
3747	DY617616	singleton
3748	DY617617	TC96633
3751	DY617620	singleton
3755	DY617624	TC99896
3756	DY617625	TC95243
3757	DY617626	TC110867
3758	DY617627	TC106963
3761	DY617629	TC103087
3764	DY617631	TC105999
3765	DY617632	TC107407
3768	DY617634	TC100822
3771	DY617636	TC94497
3772	DY617637	TC96333
3774	DY617638	TC98265
3775	DY617639	TC104466
3780	DY617643	TC101134
3781	DY617644	TC107038
3782	DY617645	TC106902
3787	DY617648	singleton
3788	DY617649	singleton
3790	DY617651	singleton
3792	DY617652	TC106608
3794	DY617654	TC106600
3795	DY617655	TC106473
3797	DY617657	singleton
3799	DY617658	singleton
3800	DY617659	singleton
3801	DY617660	TC110747
3803	DY617662	TC106122
3804	DY617663	TC108020
3807	DY617665	TC108396
3809	DY617667	TC100589
3811	DY617669	TC97010
3812	DY617670	TC96071
3816	DY617672	singleton
3818	DY617674	singleton
3822	DY617677	TC107034
3823	DY617678	TC109018
3825	DY617680	singleton
3828	DY617683	TC94611
3829	DY617684	TC104893
3834	DY617689	TC102250
3835	DY617690	singleton
3836	DY617691	TC100994
3837	DY617692	TC106487
3838	DY617693	TC101134

3840	DY617694	singleton
3842	DY617695	singleton
3843	DY617696	TC100623
3854	DY617701	TC104788
3858	DY617703	TC101187
3861	DY617705	singleton
3863	DY617707	TC93976
3865	DY617709	TC110124
3866	DY617710	TC107190
3870	DY617711	TC95398
3874	DY617715	TC99725
3876	DY617717	singleton
3881	DY617720	TC108777
3883	DY617721	TC106745
3884	DY617722	TC104024
3887	DY617723	singleton
3889	DY617725	TC108897
3890	DY617726	TC95696
3893	DY617727	TC93936
3895	DY617729	TC94812
3898	DY617731	TC102622
3901	DY617732	TC96171
3902	DY617733	TC94662
3904	DY617735	TC106735
3906	DY617737	TC101493
3911	DY617742	TC108667
3916	DY617747	TC106840
3917	DY617748	TC107608
3918	DY617749	singleton
3919	DY617750	TC100166
3920	DY617751	TC109045
3921	DY617752	TC100432
3923	DY617754	TC108713
3924	DY617755	TC102451
3928	DY617758	singleton
3929	DY617759	TC103379
3931	DY617761	TC106542
3933	DY617763	singleton
3936	DY617765	TC101155
3937	DY617766	TC95710
3938	DY617767	TC102079
3940	DY617769	TC107046
3948	DY617772	TC109834
3950	DY617774	singleton
3951	DY617775	TC100745
3953	DY617777	TC102051
3954	DY617778	TC109112
3955	DY617779	TC105121
3957	DY617781	singleton
3958	DY617782	singleton
3961	DY617784	TC103707
3962	DY617785	TC108168
3963	DY617786	TC95684
3966	DY617788	singleton
3967	DY617789	TC111967
3969	DY617791	TC95240

3974	DY617793	singleton
3976	DY617795	singleton
3979	DY617797	TC101430
3982	DY617800	TC106852
3983	DY617801	TC100687
3985	DY617802	TC103386
3986	DY617803	TC110678
3987	DY617804	TC95672
3988	DY617805	TC101092
3990	DY617807	TC101021
3992	DY617809	TC107685
3998	DY617814	TC95177
4000	DY617815	TC94805
4002	DY617817	singleton
4004	DY617819	singleton
4005	DY617820	TC107880
4006	DY617821	singleton
4007	DY617822	TC106318
4009	DY617824	TC95486
4011	DY617826	TC94783
4012	DY617827	TC94731
4013	DY617828	TC107261
4014	DY617829	TC94827
4015	DY617830	TC100933
4016	DY617831	singleton
4017	DY617832	TC94061
4018	DY617833	TC98964
4019	DY617834	TC94605
4023	DY617836	TC108307
4024	DY617837	singleton
4026	DY617839	TC106655
4028	DY617840	singleton
4031	DY617841	TC94961
4033	DY617843	TC103381
4034	DY617844	TC109341
4035	DY617845	singleton
4037	DY617846	singleton
4038	DY617847	TC101712
4039	DY617848	TC97192
4042	DY617850	TC94257
4043	DY617851	TC95705
4046	DY617853	TC106381
4049	DY617854	TC103935
4051	DY617856	TC96632
4052	DY617857	TC110003
4056	DY617859	singleton
4057	DY617860	TC106676
4060	DY617863	TC108506
4064	DY617867	singleton
4065	DY617868	TC102358
4068	DY617870	TC95066
4071	DY617871	TC95606
4074	DY617874	TC95983
4075	DY617875	singleton
4076	DY617876	TC96023
4077	DY617877	TC95486

4078	DY617878	singleton
4081	DY617881	TC101320
4084	DY617884	TC95669
4085	DY617885	TC100434
4088	DY617887	TC95699
4092	DY617889	TC107200
4095	DY617891	TC94683
4096	DY617892	TC102234
4098	DY617893	singleton
4099	DY617894	TC95402
4100	DY617895	singleton
4103	DY617898	TC98370
4104	DY617899	TC101022
4105	DY617900	singleton
4106	DY617901	TC94526
4107	DY617902	TC102728
4108	DY617903	TC94839
4109	DY617904	TC94801
4111	DY617906	singleton
4112	DY617907	TC107695
4114	DY617908	TC112456
4115	DY617909	singleton
4117	DY617911	TC111989
4118	DY617912	TC94683
4121	DY617914	TC99937
4122	DY617915	TC103781
4124	DY617917	TC94521
4126	DY617919	singleton
4127	DY617920	singleton
4128	DY617921	TC101234
4130	DY617922	TC108143
4132	DY617924	TC94701
4139	DY617928	TC101403
4141	DY617930	singleton
4142	DY617931	singleton
4143	DY617932	TC108902
4147	DY617934	TC101273
4150	DY617936	TC106537
4151	DY617937	TC96630
4158	DY617942	TC112244
4159	DY617943	singleton
4161	DY617944	TC107457
4162	DY617945	TC99899
4164	DY617947	TC100577
4165	DY617948	TC109389
4169	DY617952	TC103015
4170	DY617953	TC93981
4171	DY617954	TC100215
4174	DY617956	TC95080
4175	DY617957	singleton
4187	DY617963	TC102810
4191	DY617965	TC106705
4193	DY617967	TC104412
4197	DY617971	TC101912
4198	DY617972	TC111551
4199	DY617973	singleton

4200	DY617974	TC107109
4201	DY617975	singleton
4202	DY617976	TC107198
4203	DY617977	TC100750
4208	DY617980	TC95778
4211	DY617983	TC104256
4212	DY617984	singleton
4214	DY617985	TC110269
4215	DY617986	TC93930
4217	DY617988	TC94136
4218	DY617989	TC94474
4219	DY617990	singleton
4222	DY617993	TC93969
4223	DY617994	TC94381
4224	DY617995	singleton
4225	DY617996	TC94814
4226	DY617997	TC106794
4227	DY617998	TC107104
4228	DY617999	TC109818
4231	DY618002	TC106673
4232	DY618003	TC106399
4234	DY618005	TC107511
4237	DY618008	singleton
4238	DY618009	TC94967
4239	DY618010	TC101490
4240	DY618011	TC100802
4243	DY618013	TC105358
4244	DY618014	TC94614
4245	DY618015	TC94676
4249	DY618018	TC102273
4251	DY618020	TC101091
4256	DY618022	TC107199
4262	DY618025	TC97063
4263	DY618026	TC99622
4264	DY618027	TC100666
4265	DY618028	TC109087
4267	DY618029	singleton
4268	DY618030	TC100638
4272	DY618034	TC95272
4276	DY618038	TC94844
4277	DY618039	singleton
4282	DY618043	singleton
4283	DY618044	TC107556
4284	DY618045	TC106484
4286	DY618046	singleton
4287	DY618047	TC101498
4289	DY618048	TC108042
4291	DY618050	TC95311
4292	DY618051	TC94347
4293	DY618052	TC107166
4294	DY618053	singleton
4296	DY618055	singleton
4297	DY618056	TC94483
4299	DY618058	TC109990
4300	DY618059	TC95049
4301	DY618060	TC100231

4302	DY618061	TC94064
4307	DY618064	TC93942
4312	DY618068	TC107503
4313	DY618069	TC101632
4316	DY618070	TC108785
4317	DY618071	TC100441
4319	DY618073	TC109480
4321	DY618075	TC109273
4322	DY618076	TC97668
4323	DY618077	TC107078
4324	DY618078	TC102375
4325	DY618079	TC107377
4328	DY618081	TC108419
4329	DY618082	TC109784
4331	DY618084	TC95737
4333	DY618086	TC100436
4337	DY618090	TC101843
4338	DY618091	TC101140
4339	DY618092	TC96162
4342	DY618094	TC94421
4345	DY618097	TC106888
4348	DY618099	singleton
4352	DY618102	TC94869
4353	DY618103	TC96056
4360	DY618106	TC103203
4363	DY618108	TC111177
4364	DY618109	TC101758
4365	DY618110	TC101215
4366	DY618111	singleton
4367	DY618112	TC101659
4372	DY618116	singleton
4374	DY618118	TC100680
4377	DY618121	TC102842
4378	DY618122	TC96493
4379	DY618123	TC97287
4381	DY618125	TC112058
4382	DY618126	TC94744
4384	DY618128	TC103385
4386	DY618129	TC102911
4387	DY618130	singleton
4388	DY618131	TC109549
4391	DY618132	singleton
4398	DY618136	TC110990
4399	DY618137	TC100798
4401	DY618138	TC100941
4403	DY618139	singleton
4406	DY618141	TC108014
4409	DY618143	TC101285
4414	DY618147	TC94641
4415	DY618148	singleton
4416	DY618149	TC105901
4417	DY618150	singleton
4419	DY618152	TC98260
4421	DY618153	singleton
4425	DY618154	TC96611
4428	DY618155	singleton

4431	DY618157	TC101620
4432	DY618158	TC110374
4433	DY618159	TC94878
4434	DY618160	TC107331
4435	DY618161	TC94228
4438	DY618163	singleton
4439	DY618164	TC103017
4440	DY618165	TC104302
4441	DY618166	TC108654
4444	DY618168	TC95889
4448	DY618172	TC100539
4450	DY618173	TC109802
4451	DY618174	TC102366
4452	DY618175	TC100552
4457	DY618177	TC101622
4461	DY618180	TC95840
4465	DY618183	TC106476
4467	DY618184	TC108432
4468	DY618185	TC100760
4469	DY618186	TC102413
4472	DY618189	singleton
4473	DY618190	TC110997
4474	DY618191	singleton
4475	DY618192	singleton
4476	DY618193	TC100987
4480	DY618194	TC95480
4481	DY618195	singleton
4482	DY618196	TC100662
4485	DY618198	singleton
4486	DY618199	TC98330
4488	DY618201	singleton
4491	DY618202	TC106379
4493	DY618203	singleton
4494	DY618204	TC100885
4496	DY618205	singleton
4497	DY618206	TC108560
4498	DY618207	singleton
4501	DY618210	TC102950
4505	DY618213	TC97526
4506	DY618214	singleton
4515	DY618219	singleton
4516	DY618220	TC98406
4517	DY618221	singleton
4519	DY618223	TC95996
4520	DY618224	singleton
4521	DY618225	TC100399
4523	DY618227	TC100485
4525	DY618229	TC96059
4529	DY618232	TC101652
4532	DY618233	TC106619
4536	DY618236	singleton
4537	DY618237	TC100752
4540	DY618240	singleton
4542	DY618242	TC94650
4545	DY618245	TC96994
4546	DY618246	TC94857

4547	DY618247	TC103660
4549	DY618248	singleton
4552	DY618249	singleton
4554	DY618250	singleton
4555	DY618251	TC93960
4556	DY618252	singleton
4557	DY618253	TC94479
4558	DY618254	TC95470
4559	DY618255	TC100232
4562	DY618257	TC110202
4563	DY618258	singleton
4565	DY618259	TC94606
4568	DY618261	TC100918
4573	DY618263	TC94357
4577	DY618266	TC103210
4582	DY618269	TC94758
4584	DY618271	singleton
4591	DY618275	TC97441
4594	DY618277	TC102045
4595	DY618278	TC108633
4596	DY618279	singleton
4600	DY618282	TC102084
4605	DY618287	TC111017
4609	DY618289	TC94062
4611	DY618290	TC102650
4613	DY618291	TC101525
4621	DY618296	TC97271
4622	DY618297	TC107222
4623	DY618298	singleton
4624	DY618299	singleton
4626	DY618300	TC108069
4628	DY618301	TC101773
4630	DY618303	TC109748
4635	DY618306	TC101097
4636	DY618307	TC95787
4638	DY618309	TC100216
4639	DY618310	TC100626
4641	DY618311	TC107139
4643	DY618313	TC97017
4646	DY618315	TC110328
4650	DY618318	TC101205
4651	DY618319	TC107043
4652	DY618320	TC105164
4653	DY618321	singleton
4656	DY618324	singleton
4661	DY618326	TC107582
4662	DY618327	singleton
4675	DY618337	singleton
4683	DY618343	TC94014
4685	DY618344	TC95489
4686	DY618345	TC107333
4688	DY618347	TC102859
4691	DY618350	TC96290
4692	DY618351	TC95234
4693	DY618352	TC107519
4695	DY618354	TC111361

4701	DY618359	singleton
4702	DY618360	TC106348
4703	DY618361	singleton
4704	DY618362	TC95529
4706	DY618364	TC95558
4709	DY618366	singleton
4713	DY618369	TC94575
4715	DY618371	TC94651
4716	DY618372	TC106540
4718	DY618374	TC95016
4722	DY618377	TC94757
4723	DY618378	TC102449
4724	DY618379	TC96444
4740	DY618389	TC100148
4742	DY618391	TC100673
4744	DY618393	TC95966
4753	DY618398	TC109404
4756	DY618401	TC106930
4758	DY618402	singleton
4763	DY618404	TC107264
4764	DY618405	TC109813
4766	DY618407	TC104473
4767	DY618408	TC102099
4771	DY618410	singleton
4774	DY618412	TC109854
4777	DY618413	singleton
4779	DY618414	TC102399
4780	DY618415	TC102163
4781	DY618416	TC101356
4782	DY618417	TC100846
4783	DY618418	TC106621
4787	DY618420	TC108454
4788	DY618421	TC99597
4791	DY618424	singleton
4794	DY618427	TC95125
4796	DY618428	TC107164
4798	DY618429	TC100648
4802	DY618433	TC94066
4806	DY618436	singleton
4807	DY618437	TC107956
4809	DY618439	TC112017
4811	DY618441	TC106798
4814	DY618444	TC109229
4817	DY618447	TC112175
4821	DY618450	TC97162
4823	DY618451	TC105029
4824	DY618452	TC106356
4825	DY618453	TC98461
4827	DY618455	TC94328
4830	DY618457	singleton
4832	DY618459	TC93978
4833	DY618460	TC93939
4834	DY618461	TC95376
4836	DY618462	TC100645
4837	DY618463	singleton
4839	DY618464	TC108117

4841	DY618465	TC94571
4842	DY618466	TC106858
4843	DY618467	TC96873
4844	DY618468	singleton
4847	DY618470	TC94664
4848	DY618471	singleton
4852	DY618472	singleton
4853	DY618473	TC94958
4854	DY618474	TC94259
4855	DY618475	TC100637
4857	DY618476	TC103302
4859	DY618477	TC106368
4862	DY618479	TC94788
4864	DY618481	TC94944
4868	DY618484	TC102531
4872	DY618488	TC93958
4879	DY618494	TC104549
4885	DY618499	TC93963
