# Supplementary material for: Differentiation of Symbiotic Cells and Endosymbionts in Medicago truncatula Nodulation Are Coupled to Two Transcriptome-Switches
Source: PLoS One. 2010 Mar 4;5(3):e9519. doi: 10.1371/journal.pone.0009519 (PMC2832008; doi:10.1371/journal.pone.0009519)
Supplement: Table S5 — Primer list used for RT-qPCR experiments shown in Figure 4E and Figure 6C. (0.08 MB PDF) [file pone.0009519.s009.pdf]

cluster	sequence	name	accession n°
**constitutive gene**			
	CTTTGCTTGGTGCTGTTTAGATGG	H3-like_5	TC106487
	ATTCCAAAGGCGGCTGCATA	H3-like_3	
**cluster 1 genes**			
	GGACCAGCCGTTATCTTCAA	AC4532_for	TC106619
	TTGCTCCTGAAGGATCCAAG	AC4532_rev	
	GCTGAAGGAGATTGCAGAGG	AC0589_for	TC100398
	TCAGTCAATGCCCAGTCAAA	AC0589_rev	
	CATCAACATGGACCCAACAA	AC1560_for	TC100746
	CCGTTAGTCGCGAAAGAGTT	AC1560_rev	
**cluster 2 genes**			
	TGGTTCGGTTCAGATTGTCA	AC2044_for	TC106656
	ACGAACGCCTACACACATGA	AC2044_rev	
	TGATTCTGCCTTGTTGACAAAT	AC4494_for	TC100885
	TCACACCTTGTGACCCTGTC	AC4494_rev	
	CTCCAAATGGTTGGATGGAC	AC3440_for	TC106496
	CCATAAAAGCCAACCCATTG	AC3440_rev	
**cluster 3 genes**			
	GACAAACCACTCCCCAACAC	AC3794_for	TC106600
	ATACAGACACGTGGCAGACG	AC3794_rev	
	AGCTCCAGCAGCAGAAGAAG	AC3792_for	TC106608
	TTTTCAACCTCGACATGCAA	AC3792_rev	
	AAAAACGCATTCCCAATGTC	AC2351_for	TC107254
	GTCCTTCAAGGTTCCCATCA	AC2351_rev	
**cluster 4 genes**			
	TGTCAGATTCAGCCCTAGCA	WD40_for	TC100539
	CCCACAGCAAAATAACACCA	WD40_rev	
	CAAAAGAGGTGTCCTTCATTCTG	rip1_for	TC107261
	GAGTCGAATCTCGCCTTGTC	rip1_rev	
	TCCAATGATATGTTGGCCTTT	UDPGT_for	TC104645
	CAGCATTGTGTGCCAATTTC	UDPGT_rev	
	TTGTTCAGCTCCATGCTTTG	MtN3_for	TC109032
	CAAAGGACCCCACATTCATT	MtN3_rev	
**cluster 5 genes**			

TGCTAACTGCGTTGTGCTTC	MtN1-like_for	TC102163
TGCACTCCTGATTTTTGCAG	MtN1-like_rev	
TTCAAAGGTCTGGGACTTGG	MtN9_for	TC95584
GCCAAGTTGATCACGGTCTT	MtN9_rev	
TCGATGATGGGATACATTTGGAC	MtN6_5	TC96169
GCAAGAAGATGTAGTCAACCAGAT	MtN6_3	
AAAGGGCAGCACTGTGAAAT	AC2126_5	TC111120
AACAGCACATGGCTTAACGA	AC2126_3	
TGTTGGAGTGCCAAACAAAG	AC4482_5	TC100662
TCCAGCTCCCCTTTCTTGTA	AC4482_3	
GCTTCCAATGGAGTGGCTTA	AC1810_5	TC102486
ATTCATTTGCCAGCTCAACC	AC1810_3	

cluster 6 genes

CCCTACACACTCTCCCTCCA	enod40_5	TC94515
AGGAATCCATTGCCTTTTTGTG	enod40_3	
AACTCGACATGCACTCACCCG	enod20_5	TC100731
GGAAGAAAGTACGGGAGCTACC	enod20_3	
GAAGACGATTGGTTTGAGCAG	NAP1_5	TC102128
CCGACTTGTTGGAGAAGGAG	NAP1_3	
CTTCACACATGCAACACCAA	lipase_for	TC101756
AGCCATCAGCATCTCCAAAT	lipase_rev	
CACGGGTATGCCATGTAAGA	Mt0102_5	TC94567
CACAGTAGATGAATGGAAACCGTA	Mt0102_3	
GCGGATGGTATGCTTTAAAG	SPP5	TC95076
CTTAAAATATTGGGGTTGCT	SPP6	

cluster 7 genes

TGGTGGGCACATATTTCTTG	nodulin26_for	TC100851
TGGAACCCTCCTTGTGGTAG	nodulin26_rev	
CAGCTCTTGGGAGCAACACT	nodulin26a_for	TC100851
ATTGCAATTCCAGCCAACTC	nodulin26a_rev	
TTTTGGCCATTATCCACCAT	enod2_for	TC94518
ATTTGCAGAAAGGGCACATC	enod2_rev	
GGCTGTGCAAAGCAATACAA	enod8_for	TC100432
ATAGCCACAACATGCCACAA	enod8_rev	
ATGCGTGCTGATCCCTCTAT	nodulin25_for	TC106696
CGGGATGAGTATGAGGTCCA	nodulin25_rev	

TCTCAACTTCGAGCAACAGG	Lb1_5	TC106592
TTCAGTTGCCAGTGCATCAT	Lb1_3	

cluster 8 genes

GGAAGGCATGATGTGTTTGA	NAP2_5	TC108316
GCCACGTTCACTTGTAAGCA	NAP2_3	
TTGCTGGTGATGTGCTTGAT	basicblue_for	TC93931
CTTCATCCCAGATTGGCAGT	basicblue_rev	
AACTGATTTCGGCTTGATGG	Mt0344_5	TC101807
TCATAGGCAGGAGCAAACCT	Mt0344_3	
TGGCTCAGTTTCTTCTCTTTGTT	NCR001_5	TC106577
ATGTGATGTCCCCTGGTTTC	Mt0068_3	
